# Isolation, Structural Elucidation, and *α*-Glucosidase Inhibitory Activities of Triterpenoid Lactones and Their Relevant Biogenetic Constituents from *Ganoderma resinaceum*

**DOI:** 10.3390/molecules23061391

**Published:** 2018-06-08

**Authors:** Xian-Qiang Chen, Li-Gen Lin, Jing Zhao, Ling-Xiao Chen, Yu-Ping Tang, De-Lun Luo, Shao-Ping Li

**Affiliations:** 1 State Key Laboratory of Quality Research in Chinese Medicine, Institute of Chinese Medical Sciences, University of Macau, Macau 999078, China; yb27528@umac.mo (X.-Q.C.); LigenL@umac.mo (L.-G.L.); yb47517@umac.mo (L.-X.C.); 2 Innovative Institute of Chinese Medicine and Pharmacy, Chengdu University of Traditional Chinese Medicine, Chengdu 611730, China; ldl@mirbay.com; 3 Jiangsu Collaborative Innovation Center of Chinese Medicinal Resources Industrialization, Jiangsu Key Laboratory for High Technology Research of TCM Formulae, National and Local Collaborative Engineering Center of Chinese Medicinal Resources Industrialization and Formulae Innovative Medicine, Nanjing University of Chinese Medicine, Nanjing 210023, China; yupingtang@njutcm.edu.cn

**Keywords:** *Ganoderma resinaceum*, triterpenoid, triterpenoid lactone, oxaspirolactone, *α*-glucosidase inhibitory activity

## Abstract

*Ganoderma resinaceum* has been used as an ethnomedicine for lowering blood sugar. To clarify the bioactive chemical constituents contributing to lower blood sugar, chemical investigation on the fruiting bodies of *Ganoderma resinaceum* was conducted by chromatographic techniques, and led to the isolation of 14 compounds. Their structures were elucidated as triterpenoid lactones (**1**–**4** and **8**) and ganoderma acids (**5**–**7** and **9**–**14**) based on the analysis of extensive spectroscopy (mass spectrometry (MS), nuclear magnetic resonance (NMR), infrared (IR), and ultraviolet (UV)) and comparison with literature data. Compounds **3**, **5**, **6**, and **9**–**14** were evaluated for *α*-glucosidase inhibitory activity. Compounds **1**–**7** are new compounds. Compounds **1**–**4** and **8** were characteristic of an oxaspirolactone moiety, consisting of a five-membered ether ring, a five-membered lactone ring, and a characteristic C-23 spiro carbon. It is rare for natural products that such an oxaspirolactone moiety occurred in the lanostane-type triterpenoids. Compounds **5**–**7** and **9**–**14** may be important intermediates of the biosynthetic pathways of **1**–**4** and **8**. Compounds **1** and **2** showed more potent inhibitory activity against *α*-glucosidase compared with the positive control drug acarbose with IC_50_ value of 0.75 ± 0.018 mM and 1.64 ± 0.022 mM, respectively.

## 1. Introduction

*Ganoderma resinaceum* has long been used as an ethnomedicine to treat hyperglycemia, liver diseases, and immunoregulation [1,2]. Lanostane-type triterpenoids [2,3], nortriterpenoids [4], meroterpenoids [5], steroids [6], phenolic compounds [7], and polysaccharides [8] had been reported from the fruiting bodies and cultured mycelia of *G. resinaceum*. These metabolites and crude extracts of *G. resinaceum* showed various potential biological activities, such as anticancer [9,10], hepatoprotection [2], antimicrobial activity [11], antioxidant activity [7], and inhibitory activities against acetyl cholinesterase, tyrosinase, *α*-amylase, and *α*-glucosidase [3,7].

**Figure 1:**
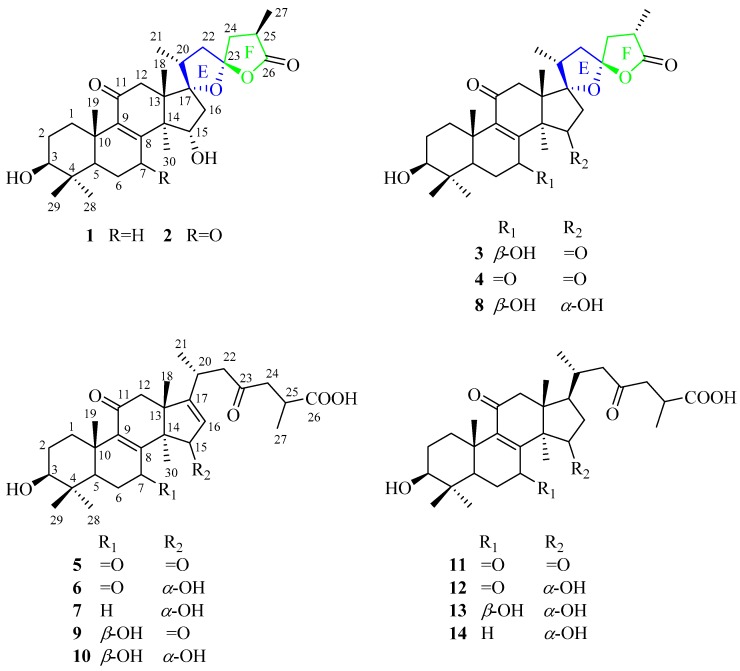
Structure of compounds **1**–**14**.

Our team has reported 48 triterpenoids, 16 nortriterpenoids, 9 meroterpenoids, and 8 steroids isolated from the fruiting bodies of *G. resinaceum* in the previous studies [3,4,5,6]. As a continuous research to investigate biological secondary metabolites of *G. resinaceum*, phytochemical investigation of *G. resinaceum* led to the isolation and identification of seven new triterpenoids (**1**–**7**) and seven known compounds, **8**–**14** (Figure 1). Compounds **1**–**4** and **8**, triterpenoid lactones, contain an oxaspirolactone moiety consisting of a five-membered ether ring, a five-membered lactone ring, and a characteristic C-23 spiro carbon, which is rare for lanostane-type triterpenoids from natural products. Herein, we report the isolation and structural elucidation of triterpenoid lactones (**1**–**4** and **8**) and their relevant biogenetic chemical constituents (**5**–**7** and **9**–**14**), and their *α*-glucosidase inhibitory activities.

## 2. Results

Fourteen compounds were isolated from the ethanol extract of *G. resinaceum* by various column chromatography, including silica gel, ODS gel, MCI gel, Sephadex LH-20, and semipreparative high performance liquid chromatography (HPLC). Their structures were identified as triterpenoid lactones (**1**–**4** and **8**) and ganoderma acids (**5**–**7** and **9**–**14**). Compounds **1** and **2** are strong *α*-glucosidase inhibitors. Structural elucidation of new compounds (**1**–**7**) and bioassay are as follows.

### 2.1. Structural Elucidation

**Figure 2:**
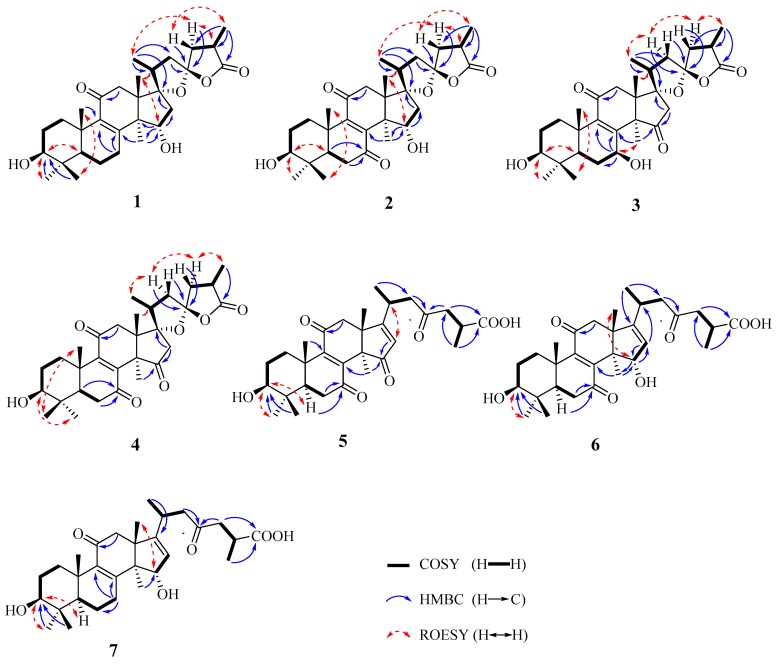
The key COSY, HMBC, and ROESY correlations of **1**–**7**. Bold line (**–**): hydrogen-hydrogen correlation in the correlation spectroscopy (COSY) spectrum. One-way arrows (→): heteronuclear multiple bond correlation from hydrogen to carbon in the heteronuclear multiple bond correlation (HMBC) spectrum. Double sided arrows (↔): the correlations between hydrogen and hydrogen in the rotating-frame nuclear overhauser effect correlation spectroscopy (ROESY) spectrum.

Compound **1** was obtained as a white powder. Its molecular formula was defined as C_30_H_44_O_6_ by high resolution electrospray ionization mass spectrometry (HRESIMS) at *m/z* 499.3033 [M−H]^−^ (calcd for C_30_H_43_O_6_, 499.3065) and 1D NMR data (Table 1). Its IR spectrum showed the presence of hydroxy (3471 cm^−1^), carbonyl (1767 cm^−1^), and *α*,*β*-unsaturated carbonyl (1635 cm^−1^) groups. The UV spectrum showed an absorption band to 258 nm due to the presence of *α*,*β*-unsaturated carbonyl group. The ^1^H NMR spectrum displayed seven methyl signals [*δ*_H_ 1.15 (3H, s), 1.42 (3H, s), 0.91 (3H, d), 1.23 (3H, d), 1.27 (3H, s), 1.13 (3H, s), and 1.68 (3H, s)] and two oxygenated methine signals (*δ*_H_ 3.52 and 4.72). The ^13^C NMR and HSQC spectra revealed the presence of 30 carbon signals consisting of seven methyls, eight methylenes, five methines including two oxygenated methines at *δ*_C_ 77.8 and 72.9, six sp^3^ quaternary carbons (two oxygenated carbon signals at *δ*_C_ 95.6 and 113.3), one ketone group (*δ*_C_ 198.6), one carboxylic group (*δ*_C_ 178.7), and two olefinic carbons (*δ*_C_ 165.2 and 139.9). The above spectroscopic data suggested **1** to be lanostane-type triterpenoid. The rings A–D were established by the heteronuclear multiple bond correlation (HMBC) and ^1^H–^1^H correlation spectroscopy (COSY) experiment (Figure 2). The key HMBC correlations from H-28 (*δ*_H_ 1.27) and H-29 (*δ*_H_ 1.13) to C-3 (*δ*_C_ 77.8), combined with ^1^H–^1^H COSY correlations of H-3 (*δ*_H_ 3.52) with H-2 (*δ*_H_ 1.99 and 2.06), confirmed the presence of 3-OH. The *α*,*β*-unsaturated ketone system at C-8/C-9/C-11 was assigned by the HMBC correlations from H-7 (*δ*_H_ 2.96 and 2.76) to C-8 (*δ*_C_ 165.2) and C-9 (*δ*_C_ 139.9), H-19 (*δ*_H_ 1.42) and H-12 (*δ*_H_ 2.46) to C-9 (*δ*_C_ 139.9), and H-12 (*δ*_H_ 3.38 and 2.46) to C-11 (*δ*_C_ 198.6). The 15-OH was established on the basis of the HMBC correlations of H-15 (*δ*_H_ 4.72) with C-8 (*δ*_C_ 165.2), C-14 (*δ*_C_ 54.4), C-16 (*δ*_C_ 47.3), and C-30 (*δ*_C_ 21.8), and H-30 (*δ*_H_ 1.68) with C-15 (*δ*_C_ 72.9), as well as the COSY cross peaks of H-15 (*δ*_H_ 4.72) with H-16 (*δ*_H_ 2.52 and 2.82). Apart from seven degrees of unsaturation ascribing to the rings A–D, two carbonyls, and one double bond, the remaining two degrees of unsaturation indicated that **1** should have two rings in the side chain. According to a carboxylic carbon (*δ*_C_ 178.7), an acetal secondary carbon (*δ*_C_ 113.3), and a downfield oxygenated sp^3^ tertiary carbon (*δ*_C_ 95.6), it was assumed that the structure of **1** possessed a novel oxaspirolactone moiety consisting of a five-membered ether ring, a five-membered lactone ring, and a characteristic C-23 spiro carbon. The hypothesis of oxaspirolactone moiety was supported by the HMBC correlations from H-21 (*δ*_H_ 0.91) to C-17 (*δ*_C_ 95.6), C-20 (*δ*_C_ 43.7), and C-22 (*δ*_C_ 44.62), H-27 (*δ*_H_ 1.23) to C-24 (*δ*_C_ 44.61), C-25 (*δ*_C_ 35.7), and C-26 (*δ*_C_ 178.7), H-22 (*δ*_H_ 2.64 and 1.76) and H-24 (*δ*_H_ 2.36 and 1.96) to C-23 (*δ*_C_ 113.3), together with the COSY correlations of H-21 (*δ*_H_ 0.91) and H-22 (*δ*_H_ 2.64) with H-20 (*δ*_H_ 2.14), and H-24 (*δ*_H_ 2.36 and 1.96) and H-27 (*δ*_H_ 1.23) with H-25 (*δ*_H_ 3.02). The planar structure of **1** was highly similar to that of ganotropic acid (**8**), except for the disappearance of hydroxy group attached to C-7 in **1**.

The absolute configuration of C-17 and C-23 has an important bearing on chemical shifts of C-20, C-22, and C-24 due to the anisotropic effect of the lactone oxygen atom. The ^13^C NMR chemical shifts of C-20, C-22, and C-24 for (17*S*,23*S*)-isomer were almost equal, however, those of (17*S*,23*R*), (17*R*,23*S*), and (17*R*,23*R*)-isomers showed dramatical differences larger than 5 ppm [12,13]. Therefore, the absolute configurations of C-17 and C-23 for **1** were highly close to that of (17*S*,23*S*)-isomer reported in the literature [12], and both determined to be *S*-configuration based on comparison of NMR spectroscopic data (C-20 (*δ*_C_ 43.4), C-22 (*δ*_C_ 44.6), and C-24 (*δ*_C_ 44.9)) with those of similar structures. The Me-21 and Me-27 were assigned as *α*- and *β*-orientation, respectively, based on the ROESY correlations of H-18 (*δ*_H_ 1.15) with H-20 (*δ*_H_ 2.14), H-24*α* (*δ*_H_ 2.36) with H-21 (*δ*_H_ 0.91) and H-25 (*δ*_H_ 3.02), and H-24*β* (*δ*_H_ 1.96) with H-27 (*δ*_H_ 1.23). The key ROESY correlations of H-19 (*δ*_H_ 1.42) with H-29 (*δ*_H_ 1.13), H-3 (*δ*_H_ 3.52) with H-5 (*δ*_H_ 1.18) and H-28 (*δ*_H_ 1.27), and H-18 (*δ*_H_ 1.15) with H-15 (*δ*_H_ 4.72) assigned 3-OH and 15-OH as *β*- and *α*-orientation, respectively. Accordingly, compound **1** was identified as (17*S*,23*S*)-17,23-epoxy-3*β*,15*α*-dihydroxy-11-oxo-5*α*-lanosta-8-en-26,23-olide.

Compound **2** was isolated as a white powder. Its molecular formula was determined to be C_30_H_42_O_7_ due to the HRESIMS ion peak at *m/z* 513.2852 [M−H]^−^ (calcd for C_30_H_41_O_7_, 513.2852), implying ten degrees of unsaturation. The IR and UV spectra showed the presence of hydroxy (3442 cm^−1^), carbonyl (1767 cm^−1^), and *α*,*β*-unsaturated carbonyl (1665 cm^−1^ and 272 nm) groups. The ^1^H NMR, ^13^C NMR (Table 1), and HSQC spectra indicated the presence of seven methyls (*δ*_H_ 0.89 (3H, s), 1.01 (3H, d), 1.03 (3H, s), 1.05 (3H, s), 1.24 (3H, s), 1.25 (3H, d), and 1.29 (3H, s)), seven methylenes, five methines including two oxygenated methine at *δ*_C_ 77.3 and 72.7, six sp^3^ quaternary carbons including two oxygenated quaternary carbons at *δ*_C_ 94.7 and 112.7, one tetrasubstituted olefinic group (*δ*_C_ 150.3 and 154.5), and three carbonyl groups (*δ*_C_ 205.0, 201.8, and 178.5). The abovementioned data suggested **2** to be similar to **1** except for the presence of an additional ketone in **2**. The HMBC correlations from H-5 (*δ*_H_ 1.55) and H-6 (*δ*_H_ 2.56 and 2.57) to C-7 (*δ*_C_ 205.0) confirmed that a ketone was located at C-7. The relative configuration of **2** was established based on the rotating-frame nuclear overhauser effect correlation spectroscopy (ROESY) experiment, coupling constant, and comparison of its NMR spectroscopic data with that of **1**. The 3-OH was determined to be *β*-oriented by the ROESY correlations (Figure 2) and the larger coupling constant (*δ*_H_ 3.29, dd, *J* = 11.4, 4.8 Hz). The ROESY correlation of H-18 (*δ*_H_ 1.05) with H-15 (*δ*_H_ 4.45) assigned 15-OH as *α*-orientation. the Me-21 and Me-27 was established as *α*-orientation and *β*-orientation, respectively, based on the ROESY correlations of H-20 (*δ*_H_ 2.26) with H-18 (*δ*_H_ 1.05), H-21 (*δ*_H_ 1.01) with H-24*α* (*δ*_H_ 2.47), and H-24*β* (*δ*_H_ 2.03) with H-27 (*δ*_H_ 1.25). Therefore, compound **2** was determined to be (17*S*,23*S*)-17,23-epoxy-3*β*,15*α*-dihydroxy-7,11-dioxo-5*α*-lanosta-8-en-26,23-olide.

Compound **3** was isolated as a white powder. Its molecular formula was defined as C_30_H_42_O_7_ by the HRESIMS ion peak at *m/z* 513.2855 [M−H]^−^ (calcd. for C_30_H_41_O_7_, 513.2852). The presence of hydroxy (3474 cm^−1^), carbonyl (1774 and 1750 cm^−1^), and *α*,*β*-unsaturated carbonyl (1656 cm^−1^ and 255 nm) groups was confirmed by the IR and UV spectra. The ^1^H NMR, ^13^C NMR (Table 1), and HSQC spectra showed the presence of seven methyls [*δ*_H_ 0.87 (3H, s), 1.04 (3H, s), 1.12 (3H, d), 1.18 (3H, s), 1.21 (3H, s), 1.29 (3H, d), and 1.41 (3H, s)], seven methylenes, five methines including two oxygenated methine groups *δ*_C_ 66.7 and 72.7, six sp^3^ quaternary carbons including two oxygenated quaternary carbons at *δ*_C_ 91.8 and 113.0, one tetrasubstituted olefinic group (*δ*_C_ 142.4 and 157.5), and three carbonyl groups (*δ*_C_ 198.1, 216.1, and 178.3). The 1D NMR spectroscopic data indicated the gross structure of **3** was same as that of **2**. The differences were that an oxygenated methine carbon signal (*δ*_C_ 66.7) resonated at higher field and a ketone carbon signal (*δ*_C_ 216.1) at lower field in **3** compared with those of **2**. The HMBC correlations from H-7 (*δ*_H_ 4.81) to C-8 (*δ*_C_ 157.5) and C-9 (*δ*_C_ 142.4), combined with the ^1^H–^1^H COSY correlations of H-7 (*δ*_H_ 4.81) with H-6 (*δ*_H_ 2.18 and 1.59), confirmed the presence of 7-OH. The ketone group at C-15 was supported by the HMBC correlations from H-30 (*δ*_H_ 1.41) and H-16 (*δ*_H_ 3.35 and 2.47) to C-15 (*δ*_C_ 216.1). The ROESY correlation of H-7 (*δ*_H_ 4.81) with H-30 (*δ*_H_ 1.41) assigned 7-OH as having *β*-orientation. The relative configurations of Me-21 and Me-27 were all determined to be *α*-orientation on the basis of the ROESY correlations of H-18 (*δ*_H_ 1.18) with H-20 (*δ*_H_ 2.38), H-22*α* (*δ*_H_ 1.93) with H-21 (*δ*_H_ 1.12) and H-24*α* (*δ*_H_ 1.93), and H-24*α* (*δ*_H_ 1.93) with H-27 (*δ*_H_ 1.29). Therefore, compound **3** was assigned as (17*S*,23*S*)-17,23-epoxy-3*β*,7*β*-dihydroxy-11,15-dioxo-5*α*-lanosta-8-en-26,23-olide.

Compound **4** was obtained as a white powder. Its molecular formula was established as C_30_H_40_O_7_ by the HRESIMS ion peak at *m/z* 511.2700 [M−H]^−^ (calcd. for C_30_H_39_O_7_, 511.2696). The IR and UV spectra displayed the presence of hydroxy (3490 cm^−1^), carbonyl (1774 and 1750 cm^−1^), and *α*,*β*-unsaturated carbonyl (1672 cm^−1^ and 265 nm) groups. A comparison of its NMR spectroscopic data with those of **3** showed their structural similarities. The only difference was the presence of ketone at C-7 in **4** instead of the hydroxy group. The ketone group was located at C-7 due to the key HMBC correlations of H-6 (*δ*_H_ 2.62 and 2.58) and H-5 (*δ*_H_ 1.63) with C-7 (*δ*_C_ 198.5). The relative configuration for **4** was determined based on the ROESY experiment and comparison of NMR spectroscopic data with those of **3**. The ROESY correlations of H-3 (*δ*_H_ 3.30) with H-28 (*δ*_H_ 1.03), H-20 (*δ*_H_ 2.38) with H-18 (*δ*_H_ 1.03), H-22*α* (*δ*_H_ 1.91) with H-21 (*δ*_H_ 1.10) and H-24*α* (*δ*_H_ 2.09), and H-24*α* (*δ*_H_ 2.09) with H-27 (*δ*_H_ 1.28) assigned 3-OH, Me-21, and Me-27, were *β*-, *α*-, and *α*-orientation, respectively. Therefore, compound **4** was established as (17*S*,23*S*)-17,23-epoxy-3*β*-hydroxy-7,11,15-trioxo-5*α*-lanosta-8-en-26,23-olide.

Compound **5** was obtained as a white powder. Its molecular formula was determined to be C_30_H_40_O_7_ based on the HRESIMS peak ion at *m/z* 511.2684 [M−H]^−^ (calcd. for C_30_H_39_O_7_, 511.2696). The ^1^H NMR, ^13^C NMR (Table 2), and HSQC spectra indicated that **5** was lanostane ganoderic acid closely similar to ganoderesin C [14]. The only difference was that **5** possessed a tetrasubstituted olefinic group between C-8 and C-9 whereas ganoderesin C did not. The tetrasubstituted olefinic group was established using HMBC correlations of H-30 (*δ*_H_ 1.39) with C-8 (*δ*_C_ 151.5), and H-19 (*δ*_H_ 1.08) with C-9 (*δ*_C_ 154.0). The planar structure of **5** was confirmed by the HMBC and ^1^H–^1^H COSY correlations (Figure 2). The ROESY correlations of H-3 (*δ*_H_ 3.18) with H-28 (*δ*_H_ 0.96) and H-5 (*δ*_H_ 1.71) assigned 3-OH as *β*-orientation. Therefore, compound **5** was elucidated as 3*β*-hydroxy-7,11,15,23-tetraoxo-5*α*-lanosta-8,16-dien-26-oic acid.

Compound **6** was isolated as a white powder. Its molecular formula was assigned as C_30_H_42_O_7_ based on the HRESIMS ion peak at *m/z* 513.2843 [M−H]^−^ (calcd. for C_30_H_41_O_7_, 513.2852). The ^1^H NMR, ^13^C NMR (Table 2), and HSQC spectra showed that **6** was lanostane-type triterpenoid closely similar to **5**. The only difference was an oxygenated methine group at C-15 in **6** instead of carbonyl group in **5**. The key HMBC correlations from H-30 (*δ*_H_ 1.17) to C-15 (*δ*_C_ 78.4), and H-15 (*δ*_H_ 4.95) to C-16 (*δ*_C_ 124.5) and C-30 (*δ*_C_ 23.2), combined with a ^1^H–^1^H COSY cross peak of H-15 (*δ*_H_ 4.95) with H-16 (*δ*_H_ 5.26), confirmed the presence of 15-OH. The 15-OH was assigned as *α*-orientation on the basis of the ROESY correlation of H-15 (*δ*_H_ 4.95) with H-30 (*δ*_H_ 1.17). Based on detail examination of HMBC, ^1^H-^1^H COSY, and ROESY experiments, compound **6** was identified as 3*β*,15*α*-dihydroxy-7,11,23-trioxo-5*α*-lanosta-8,16-dien-26-oic acid.

Compound **7** was obtained as a white powder. Its molecular formula was determined to be C_30_H_44_O_6_ by the HRESIMS ion peak at *m/z* 499.3048 [M−H]^−^ (calcd. for C_30_H_43_O_6_, 499.3060). A comparison of NMR spectroscopic data (Table 2) with those of **6** indicated their structural similarities, except for the disappearance of one ketone group at C-7 in **7**. The methylene at C-7 was confirmed by the key HMBC correlations of H-7 (*δ*_H_ 2.55) with C-6 (*δ*_C_ 18.7), C-8(*δ*_H_ 166.5), and C-9 (*δ*_H_ 140.9), as well as COSY correlations of H-6 (*δ*_H_ 1.81 and 1.51) with H-7 (*δ*_H_ 2.55). Therefore, compound **7** was identified as 3*β*,15*α*-dihydroxy-11,23-dioxo-5*α*-lanosta-8,16-dien-26-oic acid.

In addition, seven known compounds **8**–**14** were also isolated from *G*. *resinaceum*. By comparison of their NMR spectroscopic data and MS data with those reported in the literature, their structures were identified as ganotropic acid (**8**) [15], 3*β*,7*β*-dihydroxy-11,15,23-trioxo-lanost-8,16-dien-26-oic acid (**9**) [16], 3*β*,7*β*,15*β*-trihydroxy-11,23-dioxo-lanost-8,16-dien-26-oic acid (**10**) [17], ganoderic acid AM_1_ (**11**) [18], ganoderic acid K (**12**) [19], ganoderic acid C_2_ (**13**) [19], and ganolucidic acid B (**14**) [20], respectively.

### 2.2. Bioassay

In an in vitro *α*-glucosidase inhibitory assay, compounds **1**–**3**, **5**, **6**, and **9**–**14** were evaluated for *α*-glucosidase inhibitory activity. Compounds **1** and **2** exhibited more potent inhibitory activity against *α*-glucosidase compared with the positive control drug acarbose (IC_50_ value 2.76 mM) with IC_50_ value of 0.75 ± 0.018 mM and 1.64 ± 0.022 mM, respectively. The inhibition rate of other compounds was less than 50% at the concentration of 3 mM (Table S1 in Supplementary Material), suggesting that they showed no significant inhibitory activity against *α*-glucosidase compared with acarbose; therefore, their IC_50_ value were not measured. The bioassay results are in line with the structure–activity relationships of *α*-glucosidase inhibitory activity of triterpenoids, which was summarized in our previous report [3].

## 3. Discussion

**Figure 3:**
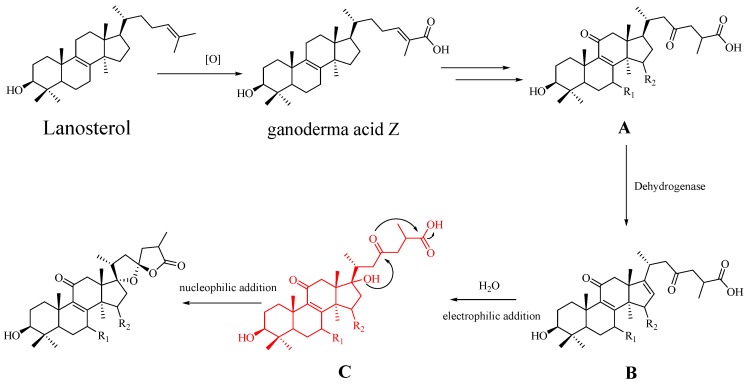
Plausible biogenetic pathway for **1**–**4** and **8**. **A**, **B**, and **C** are supposed intermediates in the biosynthetic pathway of compounds **1**–**4** and **8**. Intermediate **A** included compounds **11**–**14**. Intermediate **B** included compounds **5**–**7**, **9**, and **10**. At present, the representative compounds of intermediate **C** are not obtained from *Ganoderma resinaceum*.

Fourteen triterpenoids including seven new triterpenoids (**1**–**7**) and seven known analogues (**8**–**14**) were isolated from the fruiting bodies of *G*. *resinaceum*. The IC_50_ values of **1** and **2** are lower than that of the positive control drug acarbose, suggesting that **1** and **2** are strong *α*-glucosidase inhibitors. Compounds **1**–**4** and **8** were determined to be triterpenoid lactones which possessed an oxaspirolactone moiety in the side chain, consisting of a five-membered ether ring, a five-membered lactone ring, and a characteristic C-23 spiro carbon. It is rare that such an oxaspirolactone moiety ring occurred in the lanostane-type triterpenoids. At present, no more than 40 lanostane-type triterpenoids isolated from natural products possessed such an oxaspirolactone moiety [12,13,21]. We proposed that compounds **1**–**4** and **8** were biogenetically derived from lanosterol derived by the mevalonic acid pathway. In the biosynthetic pathway (Figure 3), lanosterol undergoing multistep oxidation and double bond addition reactions would give key intermediate A, such as **11**–**14**. Intermediate A would be further transformed into intermediate B (**5**–**7**, **9**, and **10**) via a reaction catalyzed by dehydrogenase. Intermediate B is oxidized via electrophilic addition reaction to generate intermediate C. The final and interesting phase of the biosynthesis involves the formation of the oxaspiro-E/F ring moiety through tandem cyclization. The hydroxy at C-17 attacks ketone via nucleophilic addition reaction, inducing tandem cyclization reaction to generate **1**–**4** and **8**. Of note, Me-27 is *α*- or *β*-orientation in **1**–**4** and **8**, this phenomenon allows us to tentatively hypothesize that the carboxylic group at C-26 was randomly attacked via nucleophilic addition reaction in the process of free rotation of side chain. Notably, compounds **1**–**4** and **8** provide unique insights into the biosynthetic pathway of lanostane-type triterpenoids in *Ganoderma* genus, for the formation of oxaspirolactone moiety involved the tandem cyclization reaction.

## 4. Materials and Methods

### 4.1. General Experimental Procedures

NMR spectra data were recorded on a Bruker ascend 600 spectrometer (Bruker, Karlsruhe, Germany), with TMS used as a reference. All NMR samples were thermally equilibrated at 298 K. The mixing time of ROESY spectrum is 300 ms. The long-range coupling constant in HMBC experiment is 8.0 Hz. Optical rotations were measured on PerkinElmer Model 341 polarimeter (PerkinElmer, Waltham, MA, USA). UV spectra data were acquired using HACH DR6000 UV–visible spectrophotometer (Hach, Loveland, CO, USA). IR spectra were recorded as KBr disks on PerkinElmer Spectrum 100 Series FT-IR spectrometers (PerkinElmer, Waltham, MA, USA). HRESIMS data were obtained on a LTQ Orbitrap XL™ Hybrid Ion Trap-Orbitrap FT-MS spectrometer (Thermo, Waltham, MA, USA). TLC was carried out on silica gel GF_254_ plates (Yantai Institute of Chemical Industry, Yantai, China) and spots were visualized by UV light (254 and/or 365 nm) and spraying with 10% H_2_SO_4_, followed by heating. Column chromatography was carried out using silica gel (Qingdao Marine Chemical Co. Ltd., Qingdao, China), MCI gel (CHP-20P, 75–150 μm, Mitsubishi Chemical Corporation, Tokyo, Japan), ODS (35–70 μm, Grace, Maryland, MD, USA), and Sephadex LH-20 (GE Healthcare Bio-Science AB, Uppsala, Sweden) as packing materials. Semipreparative HPLC was performed on a Shimadzu instrument (Shimadzu, Tokyo, Japan) coupled to CBM-20A system controller, LC-20AP pump, SPD-M20A Photodiode Array Detector, and SIL-10AP autosampler, and equipped with a Shimadzu PRC-ODS column (250 mm × 20 mm i.d., 15 μm). *α*-Glucosidase type I (EC 3.2.1.20) from *Saccharomyces cerevisiae* and *p*-nitrophenyl *α*-d-glucopyranoside (PNPG) were purchased from Sigma (St. Louis, MO, USA). Acarbose was purchased from Target Molecule Corp. (Boston, MA, USA). Absorbance was measured at 405 nm using a flexstation 3 multi-mode microplate reader (Molecular Devices, San Jose, CA, USA). Water bath heating was carried out in water bath (Memmert GmbH + Co. KG, Schwabach, Germany).

### 4.2. Fungal Materials

Fruiting bodies of *Ganoderma resinaceum* were purchased in December 2014 from Haikou Ruizhitang Wild Lingzhi Co., Ltd. (Hainan, China), and the sample has been collected in Changjiang (N 19.0066°, E 109.1811°), Hainan province, China. The mushroom was identified by Shaoping Li, a corresponding author of the paper, based on the micro- and macro-morphology. A voucher specimen (No. ICMS-SQC-20141201) has been deposited at Institute of Chinese Medical Sciences, University of Macau.

### 4.3. Extraction and Isolation

The dried fruiting bodies of *G. resinaceum* (48 kg) were powdered and then extracted with 95% EtOH (600 L × 2 h × 2) under reflux. The extract solution was concentrated under vacuum to afford the crude extract (2.6 kg). After removal of solvent, the extract was dispersed in water and partitioned with petroleum ether, EtOAc and *n*-BuOH, successively. The HPLC and TLC profiling of EtOAc and *n*-BuOH extract showed their similarities in constituents, hence, the EtOAc extract was merged with the *n*-BuOH extract. The mixture of EtOAc and *n*-BuOH extract was subjected to silica gel column chromatography (CC) eluted with a gradient of CHCl_3_–MeOH solvent system (100:0–0:100, *v*/*v*) to obtain three fractions (WE1–WE3).

WE1 (358 g) was separated on silica gel CC eluted with petroleum ether–acetone (100:0–0:100, *v*/*v*) to obtain four subfractions (WE11–WE14). WE12 (135 g) was chromatographed over silica gel CC eluted with petroleum ether–EtOAc (10:1–0:1, *v*/*v*) to yield fractions WE12A and WE12B. WE12B (55 g) was subjected to MCI gel CC eluted with MeOH–H_2_O (70:30–100:0, *v*/*v*) to yield seven fractions (WE12B1–WB12B7). WE12B4 (4.3 g) was separated on silica gel CC eluted with isocratic CHCl_3_–MeOH (30:70, *v*/*v*) to obtain WE12B41 and WE12B42. WE12B42 (1.4 g) was purified over silica gel CC eluted with CHCl_3_–EtOAc (4:1–2:1, *v*/*v*) and then further separated on semipreparative HPLC eluted with MeCN–H_2_O (55:45, *v*/*v*) to afford **1** (15.1 mg). WE12B5 (8.5 g) was subjected to ODS CC eluted with MeOH–H_2_O (55:50–100:0, *v*/*v*) to obtain four fractions (WE12B51–WE12B54). WE12B51 (4.0 g) was purified over silica gel CC eluted with petroleum ether–EtOAc (4:1–2:1, *v*/*v*) to yield **3** (100.8 mg). WE12B52 (2.3 g) was subjected to Sephadex LH-20 CC eluted with MeOH and then further purified over silica gel CC eluted with petroleum ether–EtOAc (2:1–1:1, *v*/*v*) to yield **2** (20.3 mg).

WE13 (93 g) was separated over silica gel CC eluted with CHCl_3_–acetone (10:1–0:1, *v*/*v*) to afford three fractions (WE13A–WE13C). WE13A (30.0 g) was subjected to MCI gel CC to yield three fractions (WE13A1–WE13A3). WE13A1 (6.1 g) was fractionated on silica gel CC eluted with CHCl_3_–acetone (10:1–8:1, *v*/*v*) to obtained three fractions (WE13A11–WE13A13). WE13A12 (680.2 mg) was subjected to Sephadex LH-20 CC eluted with MeOH and then further purified by semipreparative HPLC using MeCN-H_2_O (40:60, *v*/*v*) as mobile phase to yield **8** (201.1 mg). WE13A2 (4.1 g) was chromatographed on silica gel CC eluted with CHCl_3_–acetone (10:1–8:1, *v*/*v*) to obtained three fractions (WE13A21–WE13A23). WE13A21 (401.3 mg) was separated on Sephadex LH-20 CC eluted with CHCl_3_–MeOH (1:1, *v*/*v*) and then further purified over semipreparative HPLC eluted with MeCN–H_2_O (49:51, *v*/*v*) to yield **4** (7.1 mg). WE13A23 was further purified over semipreparative HPLC eluted with MeCN–H_2_O (40:60, *v*/*v*) to afford **6** (250.4 mg). WE13B was separated on ODS C18 CC eluted with MeOH–H_2_O (50:50–80:20, *v*/*v*) to afford four fractions (WE13B1–WE12B4). WE13B2 was subjected to silica gel CC eluted with petroleum ether–EtOAc (1:2–1:4, *v*/*v*) to yield WE13B21–WE13B23, all of which were purified over Sephadex LH-20 gel CC and then semipreparative HPLC. Consequently, compounds **9** (2.1 g), **11** (231.1 mg), and **5** (1.2 g) were isolated from WE13B21, WE13B22, and WE13B23, respectively. WE13B3 was also purified over Sephadex LH-20 gel CC and then semipreparative HPLC to afford **12** (150.4 mg).

WE2 (390.0 g) was separated on silica gel CC eluted with CHCl_3_–acetone (6:1–0:1, *v*/*v*) to obtain WE21 and WE22. WE21 was firstly subjected to ODS CC eluted with MeOH–H_2_O (40:60–70:20, *v*/*v*) to obtain WE21A–WE21D. WE21B was separated over silica gel CC eluted with CHCl_3_–acetone (6:1–0:1, *v*/*v*) to obtain four fractions (WE21B1–WE21B4). WE21B3 was chromatographed on ODS CC eluted with MeOH–H_2_O (50:50, *v*/*v*) to afford WE21B31 and WE21B32. Compounds **10** (420.7 mg) and **13** (420.4 mg) were isolated from WE21B31 and WE21B32, respectively, by semipreparative HPLC. WE21D was separated on silica gel CC eluted with CHCl_3_–acetone (6:1–0:1, *v*/*v*) to obtain WE21D1 and WE21D2. WE21D1 was purified over Sephadex LH-20 gel CC and then semipreparative HPLC to yield **14** (100.8 mg) and **7** (12.1 mg). 

#### 4.3.1. (17*S*,23*S*)-17,23-Epoxy-3*β*,15*α*-dihydroxy-11-oxo-5*α*-lanosta-8-en-26,23-olide (**1**)

White powder; [α]20D +75.7 (*c* 0.12, MeOH); UV (MeOH) λ_max_ (log *ε*) 258 (3.84) nm; IR (KBr) *v*_max_ 3471, 2975, 2933, 2869, 1767, 1635, 1578, 1454, 1413, 1378, 1326, 1287, 1226, 1186, 1151, 1119, 1083, 1042, 1006, 957, 921, 881 cm^−1^; For ^1^H NMR and ^13^C NMR spectroscopic data see Table 1; HRESIMS *m/z* 499.3033 [M−H]^−^ (calcd. for C_30_H_43_O_6_, 499.3065).

#### 4.3.2. (17*S*,23*S*)-17,23-Epoxy-3*β*,15*α*-dihydroxy-7,11-dioxo-5*α*-lanosta-8-en-26,23-olide (**2**)

White powder; [α]20D +85.2 (*c* 0.13, MeOH); UV (MeOH) λ_max_ (log *ε*) 202 (3.55), 272 (3.85) nm; IR (KBr) *v*_max_ 3442, 2967, 2932, 2866, 1767, 1665, 1459, 1384, 1344, 1263, 1182, 1158, 1119, 1095, 1057, 1027, 961, 922, 882 cm^−1^; For ^1^H NMR and ^13^C NMR spectroscopic data, see Table 1; HRESIMS *m/z* 513.2852 [M−H]^−^ (calcd. for C_30_H_41_O_7_, 513.2852).

#### 4.3.3. (17*S*,23*S*)-17,23-Epoxy-3*β*,7*β*-dihydroxy-11,15-dioxo-5*α*-lanosta-8-en-26,23-olide (**3**)

White powder; [α]20D +104.1 (*c* 0.12, MeOH); UV (MeOH) λ_max_ (log *ε*) 201 (3.79), 255 (4.17) nm; IR (KBr) *v*_max_ 3474, 2974, 2933, 2870, 1769, 1729, 1656, 1455, 1381, 1329, 1301, 1265, 1185, 1139, 1114, 1073, 1037, 959, 916, 882 cm^−1^; For ^1^H NMR and ^13^C NMR spectroscopic data, see Table 1; HRESIMS *m/z* 513.2855 [M−H]^−^ (calcd. for C_30_H_41_O_7_, 513.2852).

#### 4.3.4. (17*S*,23*S*)-17,23-Epoxy-3*β*,7*β*,15*α*-trihydroxy-11-oxo-5*α*-lanosta-8-en-26,23-olide (**4**)

White powder; [α]20D +102.8 (*c* 0.11, MeOH); UV (MeOH) λ_max_ (log *ε*) 265 (3.79) nm; IR (KBr) *v*_max_ 3490, 3040, 2976, 2932, 2867, 1774, 1750, 1672, 1455, 1382, 1328, 1258, 1188, 1141, 1075, 1031, 960, 919, 883 cm^−1^; For ^1^H NMR and ^13^C NMR spectroscopic data, see Table 1; HRESIMS *m/z* 511.2700 [M−H]^−1^ (calcd. for C_30_H_39_O_7_, 511.2696).

#### 4.3.5. 3*β*-Hydroxy-7,11,15,23-tetraoxo-5*α*-lanosta-8,16-dien-26-oic acid (**5**)

White powder; [α]20D +111.0 (*c* 0.23, MeOH); UV (MeOH) λ_max_ (log *ε*) 234 (4.02), 271 (3.80) nm; IR (KBr) *v*_max_ 3432, 2968, 2933, 2872, 1718, 1677, 1601, 1459, 1381, 1189 cm^−1^; For ^1^H and ^13^C NMR data, see Table 2; HRESIMS *m/z* 511.2684 [M−H]^−^ (calcd. for C_30_H_39_O_7_, 511.2696), 1023.5451 [2M−H]^−^ (calcd. for C_60_H_79_O_14_, 1023.5470).

#### 4.3.6. 3*β*,15*α*-Dihydroxy-7,11,23-trioxo-5*α*-lanosta-8,16-dien-26-oic acid (**6**)

White powder; [α]20D +68.6 (*c* 0.23, MeOH); UV (MeOH) λ_max_ (log *ε*) 234 (4.05), 272 (3.82) nm; IR (KBr) *v*_max_ 3424, 2970, 2938, 2875, 1709, 1686, 1664, 1461, 1406 cm^−1^; For ^1^H and ^13^C NMR data, see Table 2; HRESIMS *m/z* 513.2843 [M−H]^−^ (calcd. for C_30_H_41_O_7_, 513.2852), 1027.5767 [2M−H]^−^ (calcd. for C_60_H_83_O_14_, 1027.5783).

#### 4.3.7. 3*β*,15*α*-Dihydroxy-11,23-dioxo-5*α*-lanosta-8,16-dien-26-oic acid (**7**)

White powder; [α]20D +97.7 (*c* 0.18, MeOH); UV (MeOH) λ_max_ (log *ε*) 203 (3.77), 259 (3.77) nm; IR (KBr) *v*_max_ 3430, 2963, 2932, 2878, 1715, 1653, 1577, 1460, 1375 cm^−1^; For ^1^H and ^13^C NMR data, see Table 2; HRESIMS *m/z* 499.3048 [M−H]^−^ (calcd. for C_30_H_43_O_6_, 499.3060), 999.6182 [2M−H]^−^ (calcd. for C_60_H_87_O_12_, 999.6191).

#### 4.3.8. Bioassay

*α*-Glucosidase inhibitory activity was examined by the method described by Li et al. [22]. Acarbose, a definite *α*-glucosidase inhibitor, was used as the positive control drug.

## 5. Conclusions

Phytochemical investigation of *G. resinaceum* led to the isolation of 14 triterpenoids, including seven new triterpenoids (**1**–**7**) and seven known analogues (**8**–**14)** from the fruiting bodies of *G*. *resinaceum*. Compounds **1**–**4** and **8** were determined to be triterpenoid lactones which possessed an oxaspirolactone moiety in the side chain, consisting of a five-membered ether ring, a five-membered lactone ring, and a characteristic C-23 spiro carbon. It is rare that such an oxaspirolactone moiety ring occurred in the lanostane-type triterpenoids. Based on the analysis of plausible biogenetic pathway, compounds **5**–**7** and **9**–**14** may be important intermediates in the biosynthesis of **1**–**4** and **8**. Compounds **1** and **2** showed stronger *α*-glucosidase inhibitory activity than the positive control drug acarbose, which are strong *α*-glucosidase inhibitors with IC_50_ value of 0.75 ± 0.018 mM and 1.64 ± 0.022 mM, respectively.

## Figures and Tables

**Table 1:** ^1^H (600 MHz, CDCl_3_) and ^13^C NMR (150 MHz, CDCl_3_) spectroscopic data for **1**–**4** and **8** (*δ* in ppm, *J* in Hz).

No	1 ^a^		2		3		4		8	
	*δ*_C_	*δ*_H_	*δ*_C_	*δ*_H_	*δ*_C_	*δ*_H_	*δ*_C_	*δ*_H_	*δ*_C_	*δ*_H_
**1**	35.21, CH_2_	1.36, dt (13.2, 3.0); 3.51 m	34.1, CH_2_	1.24, m; 2.83, dt (13.2, 3.0)	34.7, CH_2_	0.97 dd (13.2, 4.2); 2.83, dt (13.2, 3.6)	33.7, CH_2_	1.34, dt (13.8, 3.6); 2.84, dt (13.8, 3.6)	34.5, CH_2_	0.92, m; 2.75, dt (13.8, 3.6)
**2**	29.0, CH_2_	1.99, m; 2.06, dt (13.8, 3.0)	27.5, CH_2_	1.70, dd (12.0, 3.6); 1.76, m	27.7, CH_2_	1.67, m	27.3, CH_2_	1.68, dt (12.0, 3.6); 1.76, m	27.5, CH_2_	1.65, m; 2.11, dd (12.6, 7.2)
**3**	77.8, CH	3.52, dd overlapped	77.3, CH	3.29, dd (11.4, 4.8)	78.2, CH	3.22, dd (11.4, 4.8)	77.5, CH	3.30, dd (11.4, 4.2)	78.2, CH	3.23, dd (11.4, 4.8)
**4**	39.7, C		38.7, C		38.9, C		39.0, C		38.6, C	
**5**	52.4, CH	1.18, br d (12.0)	49.3, CH	1.55, dd (9.6, 7.8)	49.0, CH	0.88, br d (12.0)	49.9, CH	1.63, dd overlapped	48.9, CH	0.92, d (12.0)
**6**	17.9, CH_2_	1.51, m; 1.84, m	36.2, CH_2_	2.56, m; 2.57, m	26.6, CH_2_	1.59, dt (13.2, 3.6); 2.19, dd (13.2, 8.4)	35.9, CH_2_	2.58, m; 2.62, m	27.7, CH_2_	1.61, m; 1.65, m
**7**	30.7, CH_2_	2.76, dd (21.0, 5.4); 2.96, m	205.0, C		66.7, CH	4.81, m	198.5, C		69.0, CH	4.60, br t (6.6)
**8**	165.2, C		150.3, C		157.5, C		148.9, C		158.7, C	
**9**	139.9, C		154.5, C		142.4, C		151.5, C		141.6, C	
**10**	38.4, C		40.0, C		38.6, C		40.4, C		38.5, C	
**11**	198.6, C		201.8, C		198.1, C		200.2, C		200.2, C	
**12**	47.4, CH_2_	2.46, d (16.8); 3.38, d (16.8)	47.2, CH_2_	2.35, d (16.8); 3.25, d (16.8)	44.1, CH_2_	2.48, d (16.8); 3.17, d (16.8)	44.7, CH_2_	2.48, d (16.2); 3.20, d (16.2)	47.0, CH_2_	2.27, d (15.0); 3.12, d (15.0)
**13**	50.2, C		50.7 C		48.1, C		47.3, C		49.5, C	
**14**	54.4, C		53.5, C		59.1, C		56.5, C		54.3, C	
**15**	72.9, CH	4.72, dd (9.0, 7.2)	72.7, CH	4.45, dd (9.0, 7.2)	216.1, C		207.1, C		73.0, CH	4.80, t (8.4)
**16**	47.3, CH_2_	2.52, dd (15.0, 9.0); 2.80, dd (15.0, 7.2)	44.3, CH_2_	2.32, dd (15.6, 9.0); 2.53, dd (15.6, 7.2)	48.0, CH_2_	2.47, d (20.4); 3.35, d (20.4)	48.1, CH_2_	2.45, d (16.2); 3.33, d (16.2)	44.7, CH_2_	2.29, m; 2.41, dd (15.0, 8.4)
**17**	95.6, C		94.7, C		91.8, C		92.2, C		94.7, C	
**18**	19.8, CH_3_	1.15, s	20.0, CH_3_	1.05, s	20.9, CH_3_	1.18, s	20.0, CH_3_	1.03, s	19.7, CH_3_	1.16, s
**19**	19.4, CH_3_	1.42, s	17.4, CH_3_	1.29, s	18.3, CH_3_	1.21, s	17.7, CH_3_	1.20, s	19.4, CH_3_	1.26, s
**20**	43.7, CH	2.14, m	43.6, CH	2.26, m	43.1, CH	2.38, m	43.3, CH	2.38, m	48.3, CH	2.29, m
**21**	17.9, CH_3_	0.91, d (6.6)	18.1, CH_3_	1.01, d (6.6)	18.2, CH_3_	1.12, d (6.6)	18.3, CH_3_	1.10, d (7.2)	18.1, CH_3_	1.02, d (7.2)
**22**	44.61, CH_2_	1.76 d (13.8); 2.64, dd (13.8, 6.6)	44.6, CH_2_	1.81, d (13.8); 2.73, dd (13.8, 6.6)	44.4, CH_2_	1.93, d (14.4); 2.73, dd (14.4, 7.2)	44.3, CH_2_	1.91, d (14.4); 2.73, dd (14.4, 6.6)	44.6, CH_2_	1.84, d (14.4); 2.72, dd (14.4, 6.6)
**23**	113.3, C		112.7, C		113.0, C		112.9, C		113.3, C	
**24**	44.62, CH_2_	1.96, d (12.6); 2.36, dd (12.6, 8.4)	44.9, CH_2_	2.03, dd (12.6, 11.4); 2.47, dd (12.6, 8.4)	44.5, CH_2_	2.10, dd (13.2, 11.4); 2.57, dd (13.2, 8.4)	44.6, CH_2_	2.09, dd (12.6, 11.4); 2.56, dd overlapped	44.7, CH_2_	2.05, dd (12.6, 11.4); 2.49, dd (12.6, 8.4)
**25**	35.7, CH	3.02, m	35.4, CH	2.92, m	35.4, CH	2.92, m	35.3, CH	2.94, m	35.5, CH	2.96, m
**26**	178.7, C		178.5, C		178.3, C		178.2, C		179.2, C	
**27**	15.1, CH_3_	1.23, d (7.2)	14.9, CH_3_	1.25, d (6.6)	14.9, CH_3_	1.29, d (7.2)	14.9, CH_3_	1.28, d (7.2)	14.9, CH_3_	1.28, d (7.2)
**28**	28.9, CH_3_	1.27, s	27.7, CH_3_	1.03, s	28.2, CH_3_	1.04, s	27.7, CH_3_	1.03, s	28.1, CH_3_	1.03, s
**29**	16.8, CH_3_	1.13, s	15.4, CH_3_	0.89, s	15.5, CH_3_	0.87, s	15.4, CH_3_	0.89, s	15.7, CH_3_	0.85, s
**30**	21.8, CH_3_	1.68, s	22.6, CH_3_	1.24, s	26.9, CH3	1.41, s	25.8, CH_3_	1.54, s	21.2, CH_3_	1.33, s

^a^ Recorded in pyridine-*d*_5_.

**Table 2:** ^1^H (600 MHz, CD_3_OD) and ^13^C (150 MHz, CD_3_OD) NMR spectroscopic data for **5**–**7** (*δ* in ppm, *J* in Hz).

No	5		6		7	
	*δ*_C_	*δ*_H_	*δ*_C_	*δ*_H_	*δ*_C_	*δ*_H_
**1**	35.2, CH_2_	1.37, dd (13.2, 4.2); 2.85, m	35.4, CH_2_	1.29, m; 2.92, dt (13.8, 3.6)	35.8, CH_2_	1.08, dd (13.2, 3.6); 2.99, dt (13.2, 3.6)
**2**	28.1, CH_2_	1.62, m	28.6, CH_2_	1.69, m; 1.74, m	28.6, CH_2_	1.62, m; 1.68, m
**3**	78.4, CH	3.18, dd (11.4, 4.8)	78.2, CH	3.22, dd (11.4, 4.8)	79.5, CH	3.17, dd (11.4, 4.8)
**4**	40.1, C		40.1, C		40.3, C	
**5**	50.5, CH	1.71, dd (13.8, 4.2)	51.5, CH	1.58, dd (15.0, 1.8)	53.7, CH	0.95, d (13.2)
**6**	36.0, CH_2_	2.49, d (13.8); 2.55, dd (13.8, 4.2)	36.8, CH_2_	2.55, m; 2.70, t (15.0)	18.7, CH_2_	1.51, m; 1.81, m
**7**	200.3, C		206.3, C		32.9, CH_2_	2.55, m
**8**	151.5, C		150.9, C		166.5, C	
**9**	154.0, C		155.94, C		140.9, C	
**10**	42.0, C		41.8, C		39.4, C	
**11**	200.8, C		202.6, C		201.2, C	
**12**	45.6, CH_2_	2.54, d (16.8); 3.09, d (16.8)	48.9, CH_2_	2.42, d (16.2); 3.08, d (16.2)	48.7, CH_2_	2.24, d (16.8); 2.94, d (16.8)
**13**	53.2, C		53.8, C		53.4, C	
**14**	56.6, C		55.3, C		58.0, C	
**15**	206.7, C		78.4, CH	4.95, br s	77.8, CH	4.90, br s
**16**	124.0, CH	5.65, s	124.5, CH	5.26, s	125.7, CH	5.18, s
**17**	186.7, C		155.98, C		156.3, C	
**18**	30.1, CH_3_	1.02, s	23.3, CH_3_	0.95, s	23.0, CH_3_	0.96, s
**19**	17.7, CH_3_	1.08, s	17.7, CH_3_	1.30, s	19.4, CH_3_	1.12, s
**20**	30.7, CH	2.93, m	28.5, CH	2.63, m	28.9, CH	2.62, m
**21**	20.1, CH_3_	1.05, d (6.6)	21.0, CH_3_	1.04, d (6.6)	21.0, CH_3_	1.04, d (7.2)
**22**	48.5, CH_2_	2.70, dd (17.4, 5.4); 2.88, d (17.4)	49.4, CH_2_	2.57, d (16.8); 2.77, dd (16.8, 7.2)	49.5, CH_2_	2.54, d (16.8); 2.73, dd (16.8, 7.2)
**23**	209.0, C		209.9, C		210.0, C	
**24**	47.3, CH_2_	2.49, m; 2.81, m	47.5, CH_2_	2.53, m; 2.85, m	47.5, CH_2_	2.52, dd (13.2, 8.4); 2.85, dd (13.2, 9.0)
**25**	36.2, CH	2.78, m	36.0, CH	2.82, m	36.1, CH	2.83, m
**26**	179.6, C		179.9, C		179.8, C	
**27**	17.7, CH_3_	1.09, d (7.2)	17.7, CH_3_	1.14, d (7.2)	17.7, CH_3_	1.15, d (6.6)
**28**	28.3, CH_3_	0.96, s	28.3, CH_3_	1.00, s	29.1, CH_3_	1.02, s
**29**	16.0, CH_3_	0.83, s	16.2, CH_3_	0.89, s	16.6, CH_3_	0.82, s
**30**	33.6, CH_3_	1.39, s	23.2, CH_3_	1.17, s	22.7, CH_3_	1.21, s

## Supplementary Materials

The following are available online, Spectrum copies of Mass, NMR, and IR, Table S1: Inhibition rate of compounds **3**, **5**, **6**, **9**, and **11**–**13** at the concentration of 3 mM.
