# Supplementary material for: Isolation, Structural Elucidation, and α-Glucosidase Inhibitory Activities of Triterpenoid Lactones and Their Relevant Biogenetic Constituents from Ganoderma resinaceum
Source: Molecules. 2018 Jun 8;23(6):1391. doi: 10.3390/molecules23061391 (PMC6099967; doi:10.3390/molecules23061391)
Supplement: Supplementary file 1 [file molecules-23-01391-s001.pdf]

Supplementary Materials

Isolation, Structural Elucidation, and α-Glucosidase Inhibitory Activities of Triterpenoid Lactones and Their Relevant Biogenetic Constituents from *Ganoderma resinaceum*

Xian-Qiang Chen [1], Li-Gen Lin [1], Jing Zhao [1,2,*], Ling-Xiao Chen [1], Yu-Ping Tang [3], De-Lun Luo [2] and Shao-Ping Li [1,*]

1 State Key Laboratory of Quality Research in Chinese Medicine, Institute of Chinese Medical Sciences, University of Macau, 999078 Macau, China; yb27528@umac.mo (X.-Q.C.); LigenL@umac.mo (L.-G.L.); yb47517@umac.mo (L.-X.C.)
2 Innovative Institute of Chinese Medicine and Pharmacy, Chengdu University of Traditional Chinese Medicine, 611730 Chengdu, China; ldl@mirbay.com
3 Jiangsu Collaborative Innovation Center of Chinese Medicinal Resources Industrialization, Jiangsu Key Laboratory for High Technology Research of TCM Formulae, National and Local Collaborative Engineering Center of Chinese Medicinal Resources Industrialization and Formulae Innovative Medicine, Nanjing University of Chinese Medicine, 210023 Nanjing, China; yupingtang@njutcm.edu.cn
* Correspondence: zhaojing.cpu@163.com or jingzhao@umac.mo (J.Z.); spli@umac.mo or spli@hotmail.com (S.-P.L.); Tel.: +853-8822-4692 (S.-P.L.); Fax: +853-2884-1358 (S.-P.L.)

Table of Contents

Table S1 Inhibition rate of compounds 3, 5, 6, and 9–14 at the concentration of 3 mM.................... 5

Figure S1. HRESIMS spectrum of 1.. 5

Figure S2. ^{1}H NMR (600 MHz, C_5D_5N) Spectrum of 1 6

Figure S3. ^{13}C NMR (150 MHz, C_5D_5N) spectrum of 1... 6

Figure S4. HSQC (600 MHz, C_5D_5N) spectrum of 1 7

Figure S5. HMBC (600 MHz, C_5D_5N) spectrum of 1 7

Figure S6. ^{1}H-^{1}H COSY (600 MHz, C_5D_5N) spectrum of 1 8

Figure S7. ROESY (600 MHz, C_5D_5N) spectrum of 1 8

Figure S8. IR spectrum of 1 9

Figure S9. HRESIMS spectrum of 2.. 9

Figure S10. ^{1}H NMR (600 MHz, $CDCl_3$) Spectrum of 2.. 10

Figure S11. ^{13}C NMR (150 MHz, $CDCl_3$) spectrum of 2 10

Figure S12. HSQC (600 MHz, $CDCl_3$) spectrum of 2.. 11

Figure S13. HMBC (600 MHz, $CDCl_3$) spectrum of 2... 11

Figure S14. ^{1}H-^{1}H COSY (600 MHz, $CDCl_3$) spectrum of 2.. 12

Figure S15. ROESY (600 MHz, $CDCl_3$) spectrum of 2... 12

Figure S16. IR spectrum of 2.. 13

Figure S17. HRESIMS spectrum of 3.. 13

Figure S18. ^{1}H NMR (600 MHz, $CDCl_3$) Spectrum of 3.. 14

Figure S19. ^{13}C NMR (150 MHz, $CDCl_3$) spectrum of 3 14

Figure S20. HSQC (600 MHz, $CDCl_3$) spectrum of 3.. 15

Figure S21. HMBC (600 MHz, $CDCl_3$) spectrum of 3... 15

Figure S22. ^{1}H-^{1}H COSY (600 MHz, $CDCl_3$) spectrum of 3 16

Figure S23. ROESY (600 MHz, $CDCl_3$) spectrum of 3 16

Figure S24. IR spectrum of 3 17

Figure S25. HRESIMS spectrum of 4 17

Figure S26. ^{1}H NMR (600 MHz, $CDCl_3$) Spectrum of 4 18

Figure S27. ^{13}C NMR (150 MHz, $CDCl_3$) spectrum of 4 18

Figure S28. HSQC (600 MHz, $CDCl_3$) spectrum of 4 19

Figure S29. HMBC (600 MHz, $CDCl_3$) spectrum of 4 19

Figure S30. ^{1}H-^{1}H COSY (600 MHz, $CDCl_3$) spectrum of 4 20

Figure S31. ROESY (600 MHz, $CDCl_3$) spectrum of 4 20

Figure S32. IR spectrum of 4 21

Figure S33. HRESIMS spectrum of 5 21

Figure S34. ^{1}H NMR (600 MHz, CD_3OD) spectrum of 5 22

Figure S35. ^{13}C NMR (150 MHz, CD_3OD) spectrum of 5 22

Figure S36. HSQC (600 MHz, CD_3OD) spectrum of 5 23

Figure S37. HMBC (600 MHz, CD_3OD) spectrum of 5 23

Figure S38. ^{1}H-^{1}H COSY (600 MHz, CD_3OD) spectrum of 5 24

Figure S39. ROESY (600 MHz, CD_3OD) spectrum of 5 24

Figure S40. IR spectrum of 5 25

Figure S41. HRESIMS spectrum of 6 25

Figure S42. ^{1}H NMR (600 MHz, CD_3OD) spectrum of 6 26

Figure S43. ^{13}C NMR (150 MHz, CD_3OD) spectrum of 6 26

Figure S44. HSQC (600 MHz, CD_3OD) spectrum of 6 27

Figure S45. HMBC (600 MHz, CD_3OD) spectrum of 6 27

Figure S46. ^1H-^1H COSY (600 MHz, CD_3OD) spectrum of 6 28

Figure S47. ROESY (600 MHz, CD_3OD) spectrum of 6 28

Figure S48. IR spectrum of 6 29

Figure S49. HRESIMS spectrum of 7 29

Figure S50. ^1H NMR (600 MHz, CD_3OD) spectrum of 7 30

Figure S51. ^{13}C NMR (150 MHz, CD_3OD) spectrum of 7 30

Figure S52. HSQC (600 MHz, CD_3OD) spectrum of 7 31

Figure S53. HMBC (600 MHz, CD_3OD) spectrum of 7 31

Figure S54. ^1H-^1H COSY (600 MHz, CD_3OD) spectrum of 7 32

Figure S55. ROESY (600 MHz, CD_3OD) spectrum 7 32

Figure S56. IR spectrum of 7 33

Figure S57. HRESIMS spectrum of 8 33

Figure S58. ^1H NMR (600 MHz, $CDCl_3$) Spectrum of 8 34

Figure S59. ^{13}C NMR (150 MHz, $CDCl_3$) spectrum of 8 34

Figure S60. HSQC (600 MHz, $CDCl_3$) spectrum of 8 35

Figure S61. HMBC (600 MHz, $CDCl_3$) spectrum of 8 35

Figure S62. ^1H-^1H COSY (600 MHz, $CDCl_3$) spectrum of 8 36

Figure S63. ROESY (600 MHz, $CDCl_3$) spectrum of 8 36

Figure S64. IR spectrum of 8 37

Table S1 Inhibition rate of compounds **3**, **5**, **6**, and **9–14** at the concentration of 3 mM

Compounds	Inhibition rate (%)	Compounds	Inhibition rate (%)
**3**	47.6	**11**	25.2
**5**	8.5	**12**	23.7
**6**	18.5	**13**	11.2
**9**	18.8	**14**	24.8
**10**	15.5		

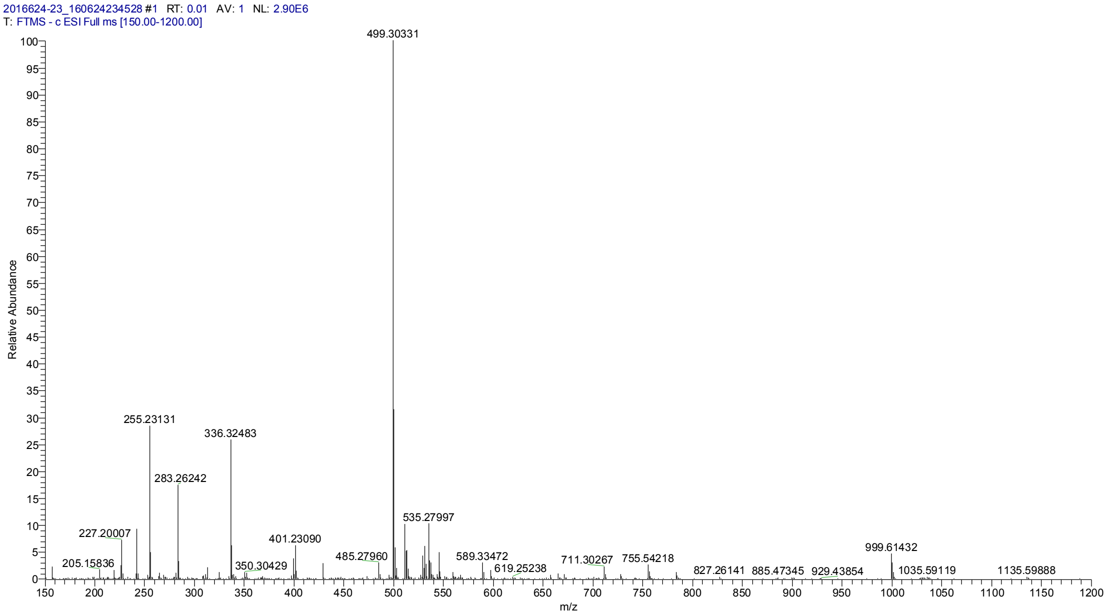

Figure S1. HRESIMS spectrum of **1**

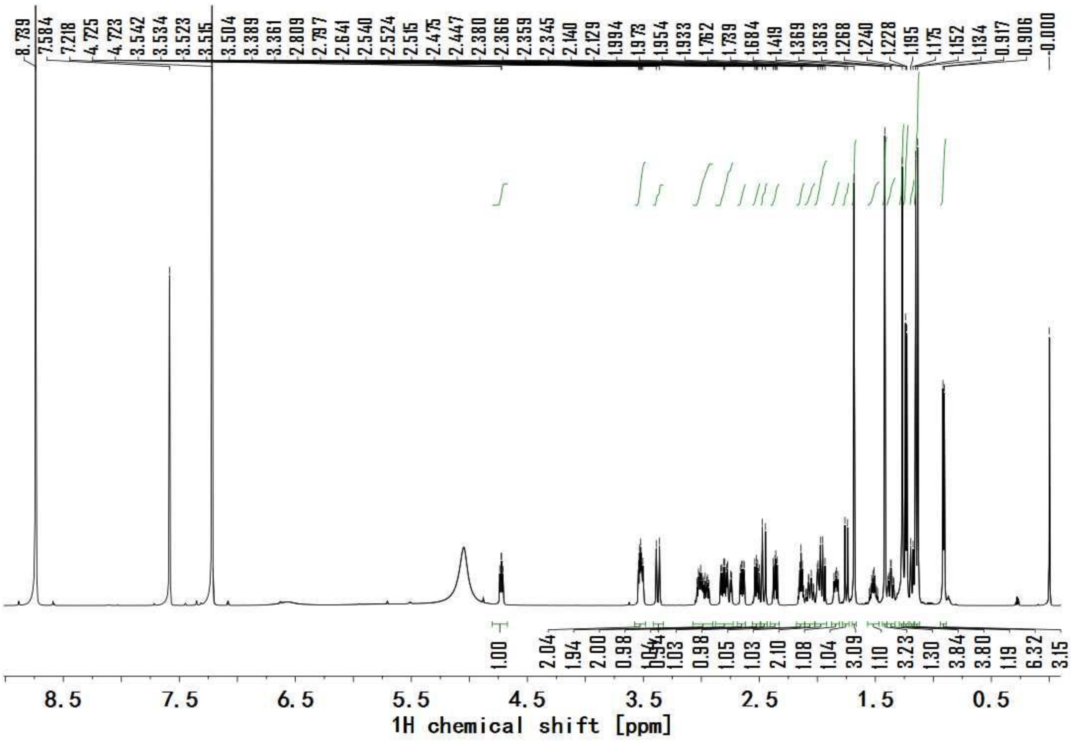

Figure S2. ^{1}H NMR (600 MHz, C_5D_5N) Spectrum of **1**

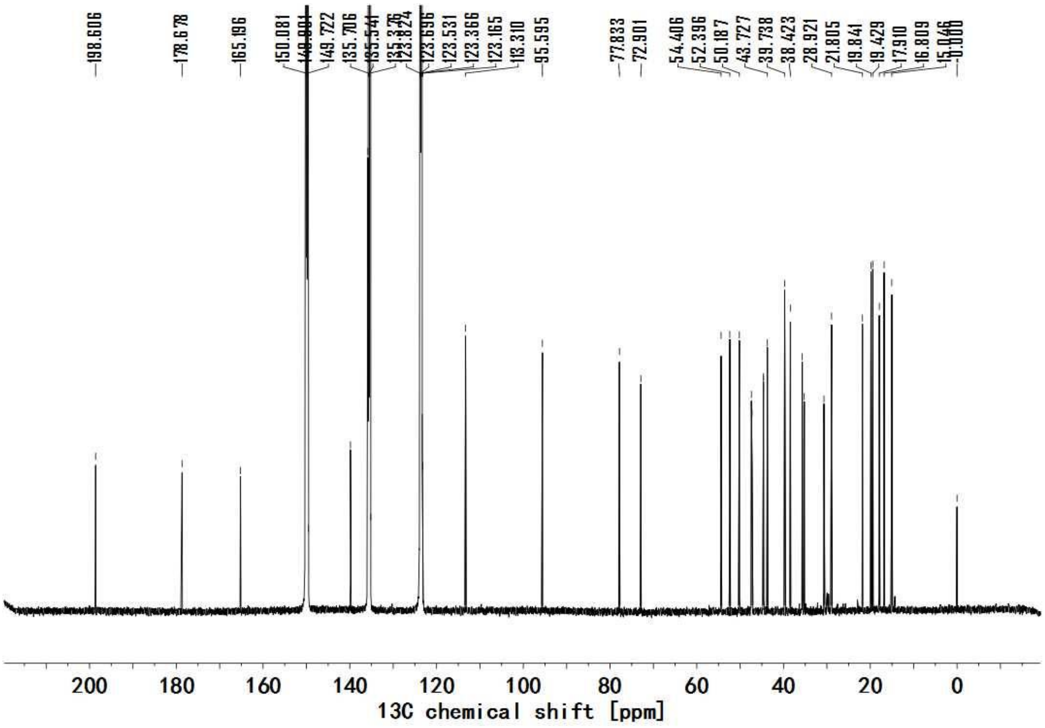

Figure S3. ^{13}C NMR (150 MHz, C_5D_5N) spectrum of **1**

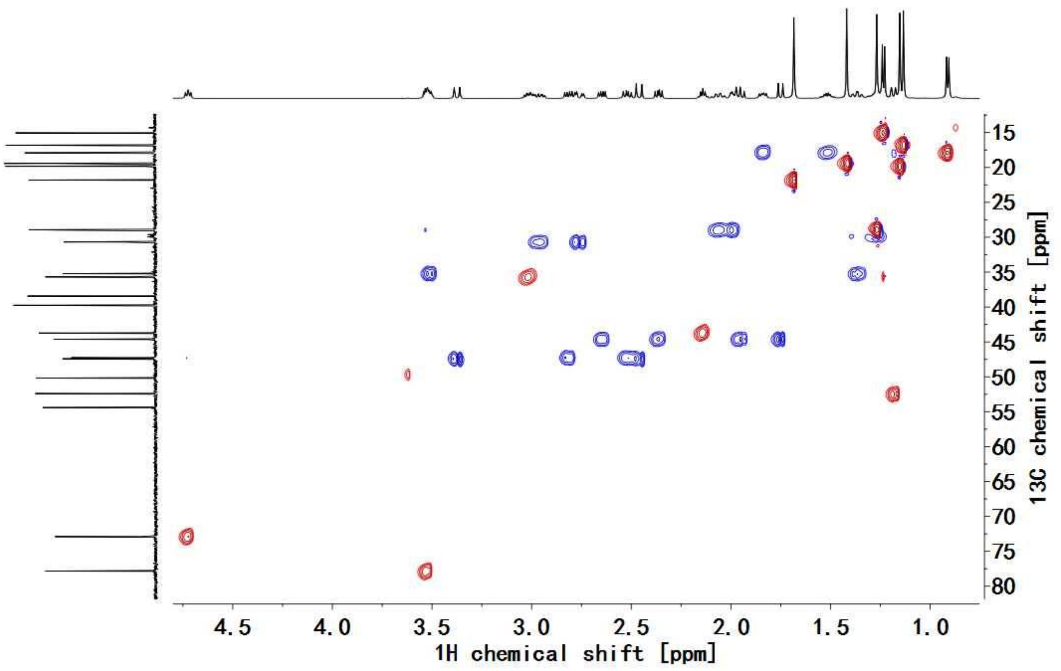

Figure S4. HSQC (600 MHz, C_5D_5N) spectrum of **1**

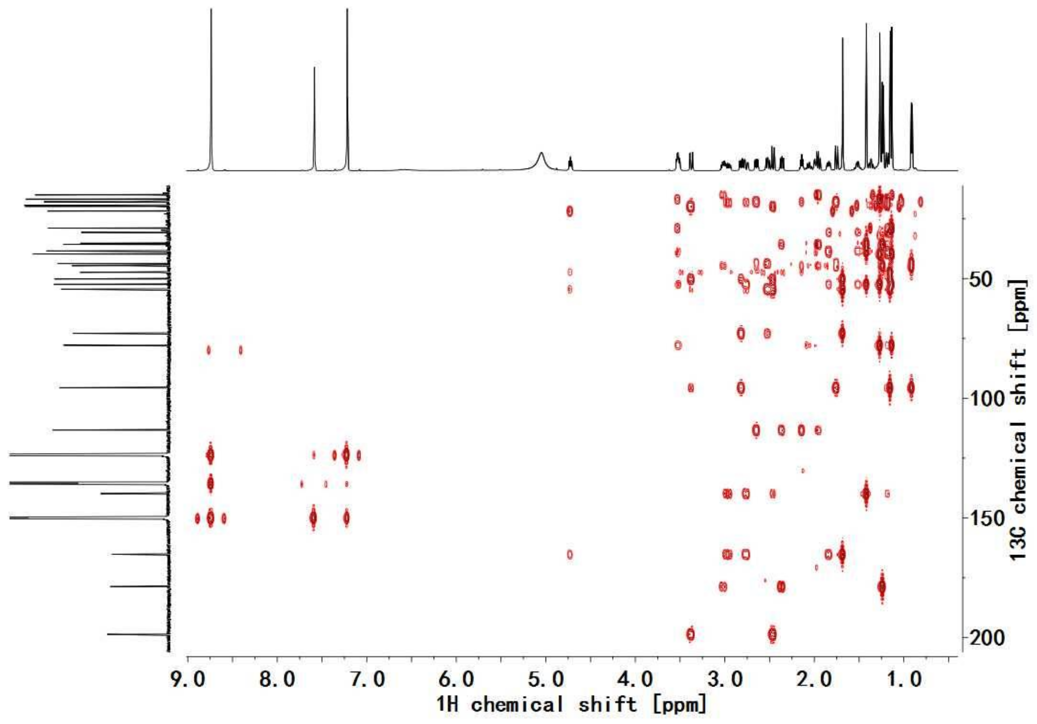

Figure S5. HMBC (600 MHz, C_5D_5N) spectrum of **1**

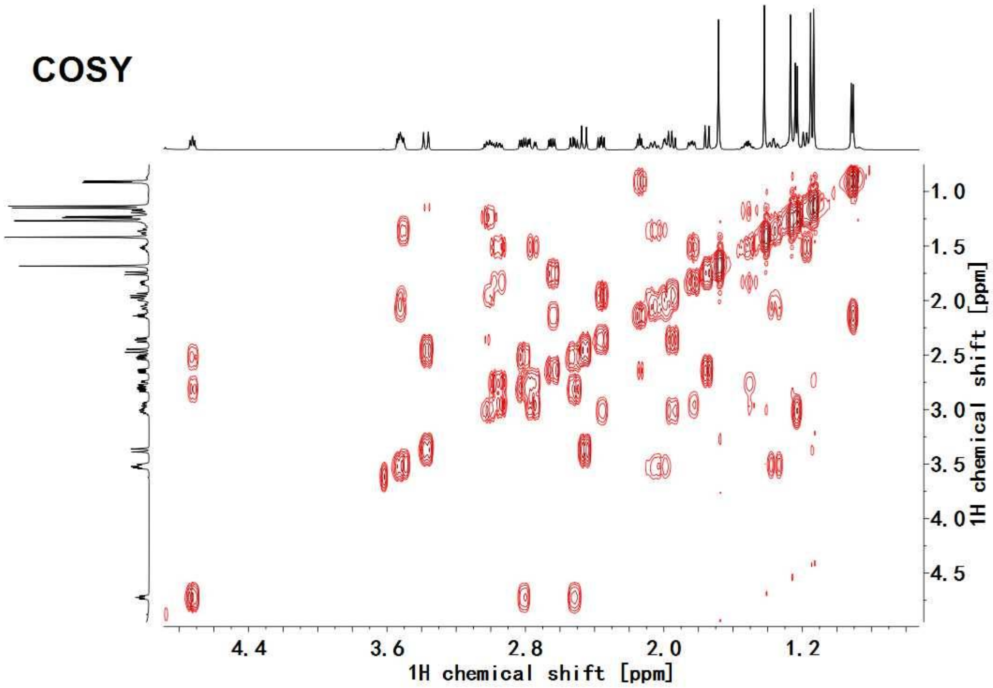

Figure S6. ^{1}H-^{1}H COSY (600 MHz, C_5D_5N) spectrum of **1**

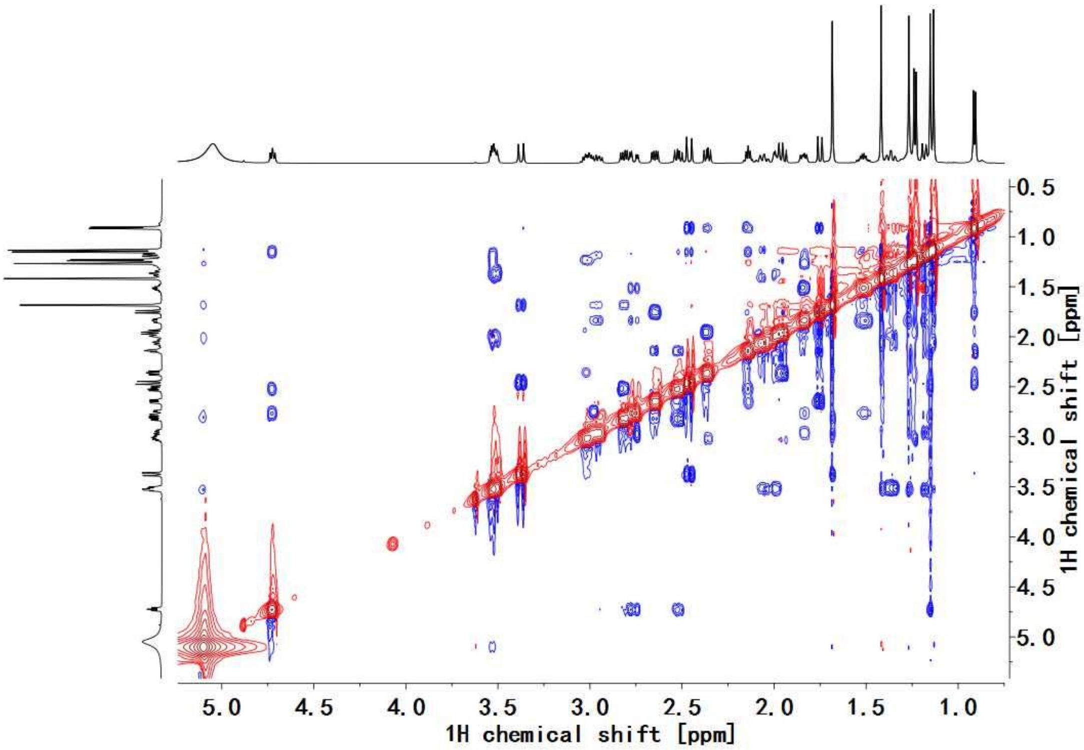

Figure S7. ROESY (600 MHz, C_5D_5N) spectrum of **1**

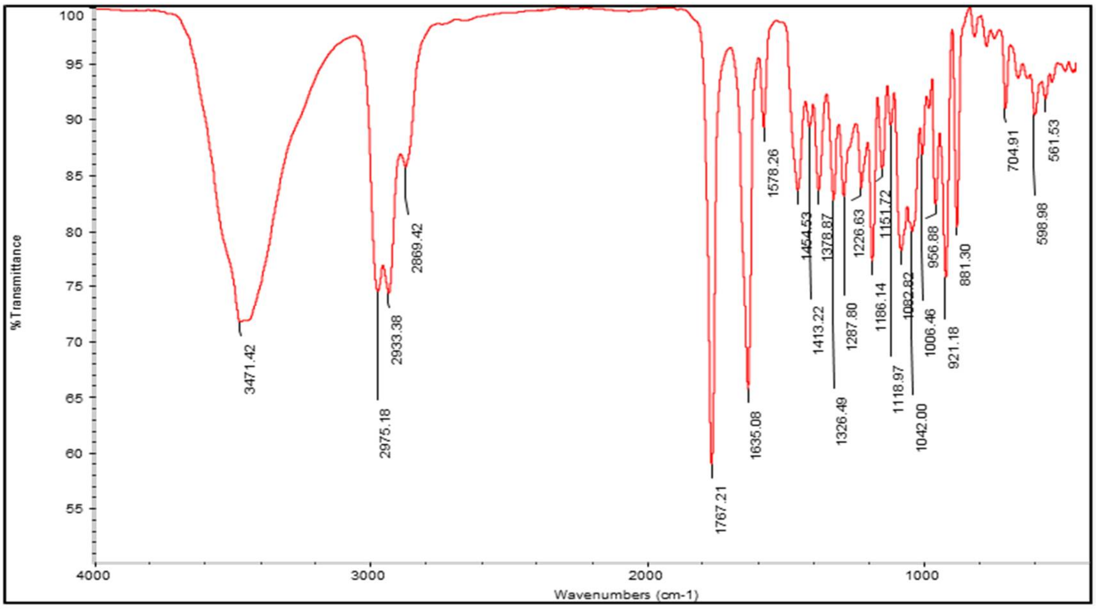

Figure S8. IR spectrum of **1**

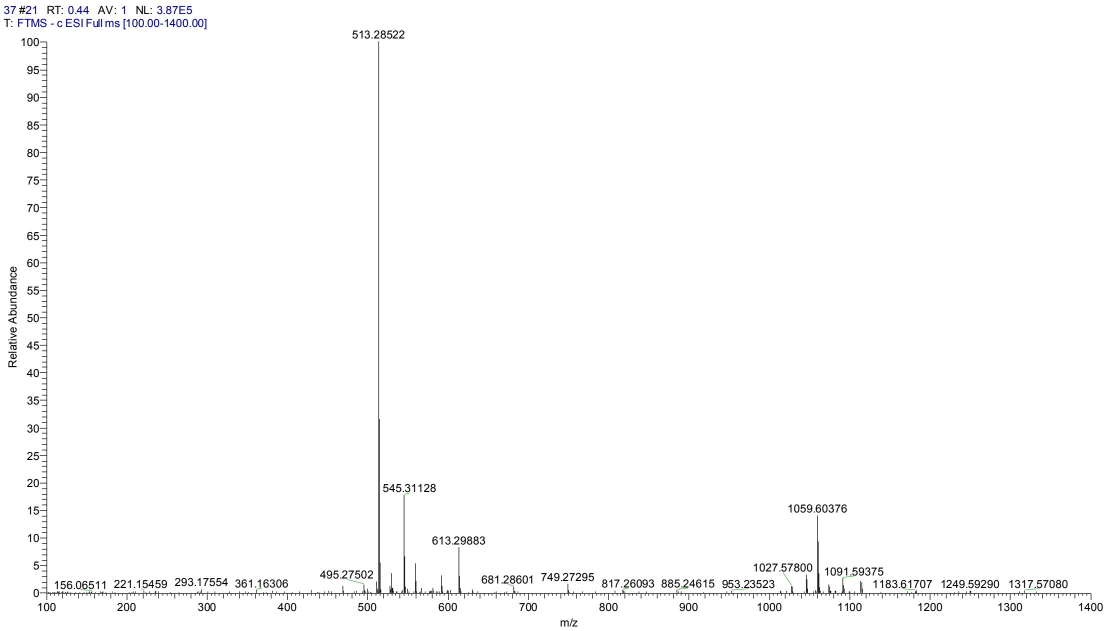

Figure S9. HRESIMS spectrum of **2**

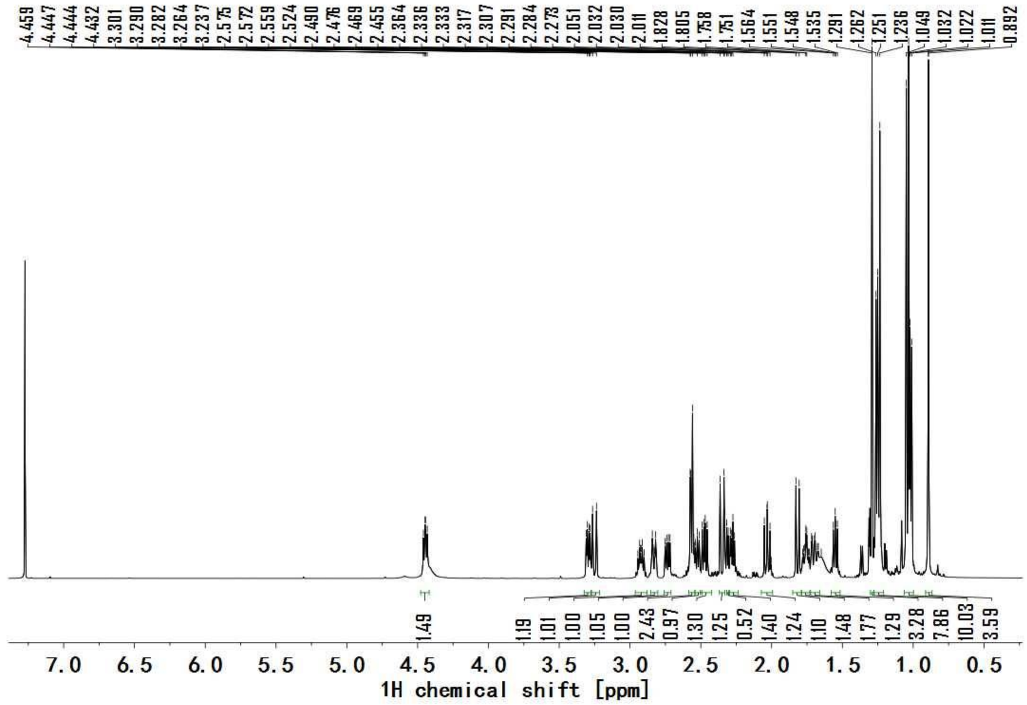

Figure S10. ^{1}H NMR (600 MHz, $CDCl_3$) Spectrum of **2**

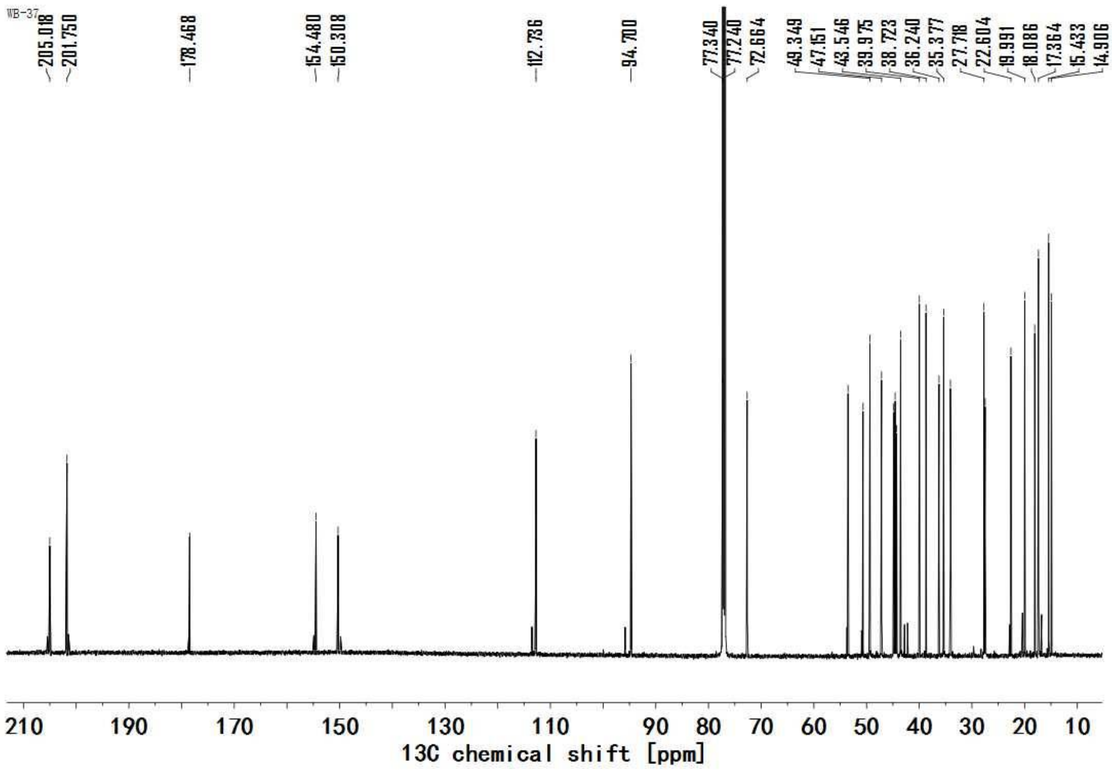

Figure S11. ^{13}C NMR (150 MHz, $CDCl_3$) spectrum of **2**

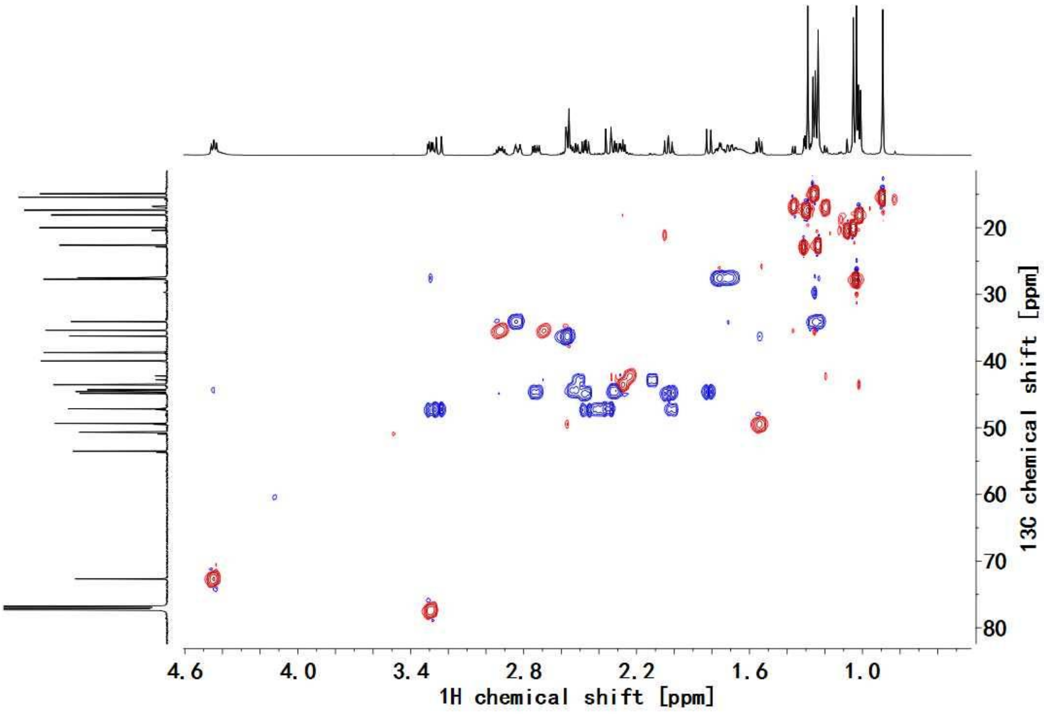

Figure S12. HSQC (600 MHz, $CDCl_3$) spectrum of **2**

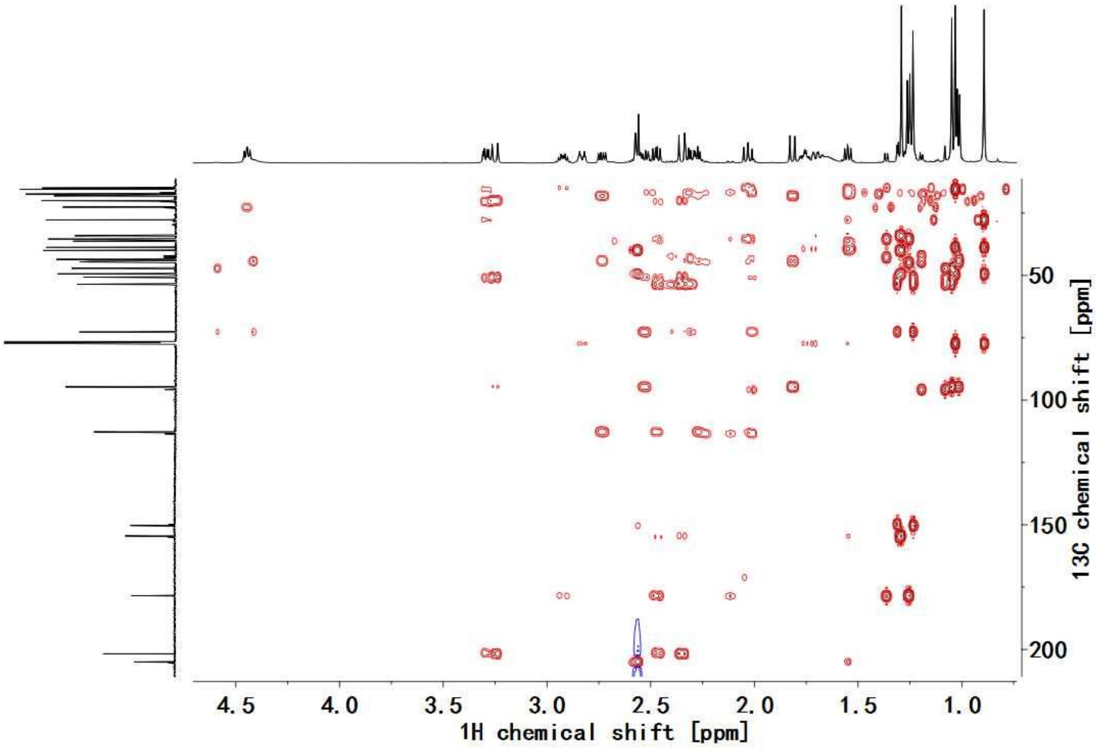

Figure S13. HMBC (600 MHz, $CDCl_3$) spectrum of **2**

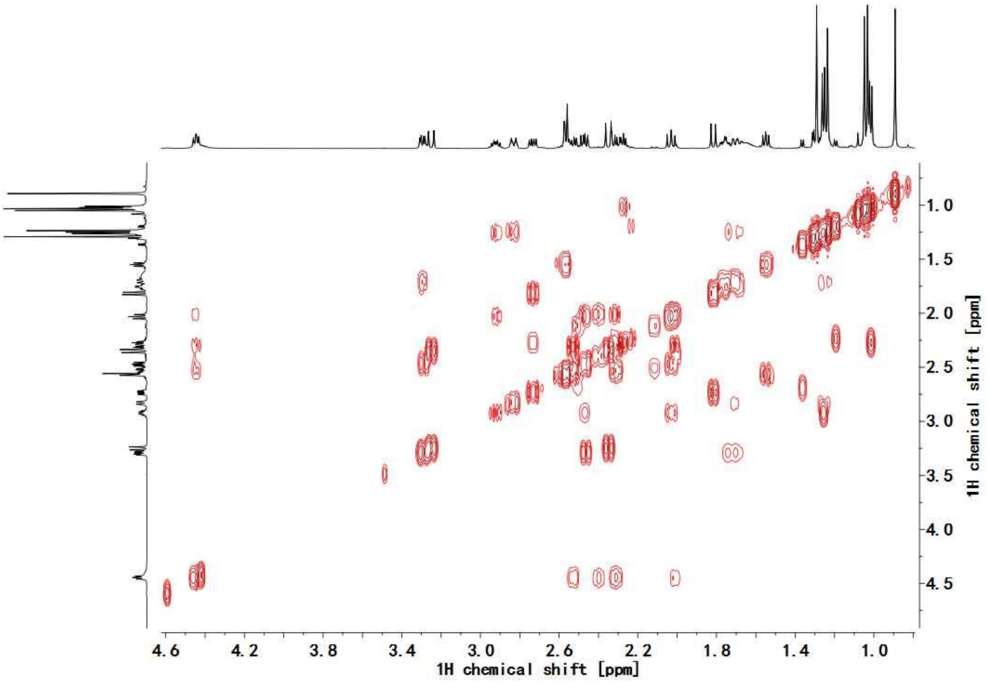

Figure S14. ^{1}H-^{1}H COSY (600 MHz, $CDCl_3$) spectrum of **2**

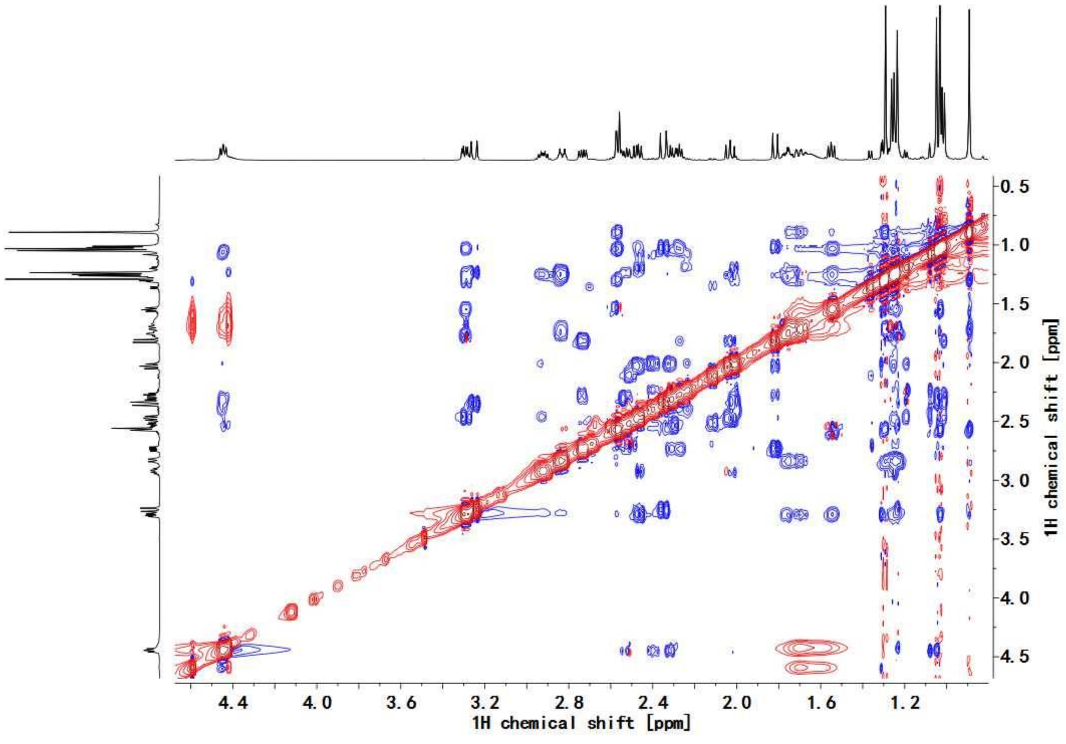

Figure S15. ROESY (600 MHz, $CDCl_3$) spectrum of **2**

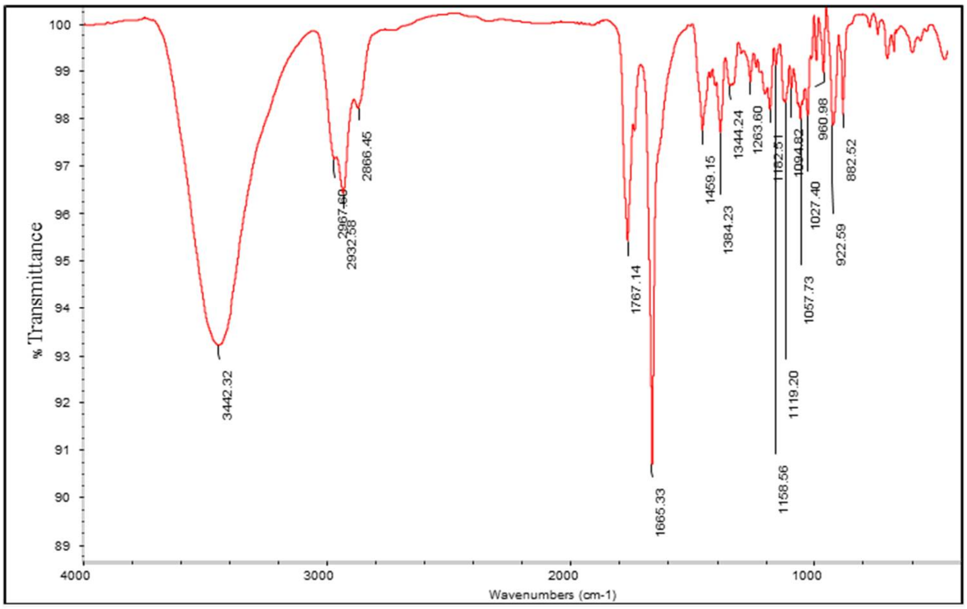

Figure S16. IR spectrum of **2**

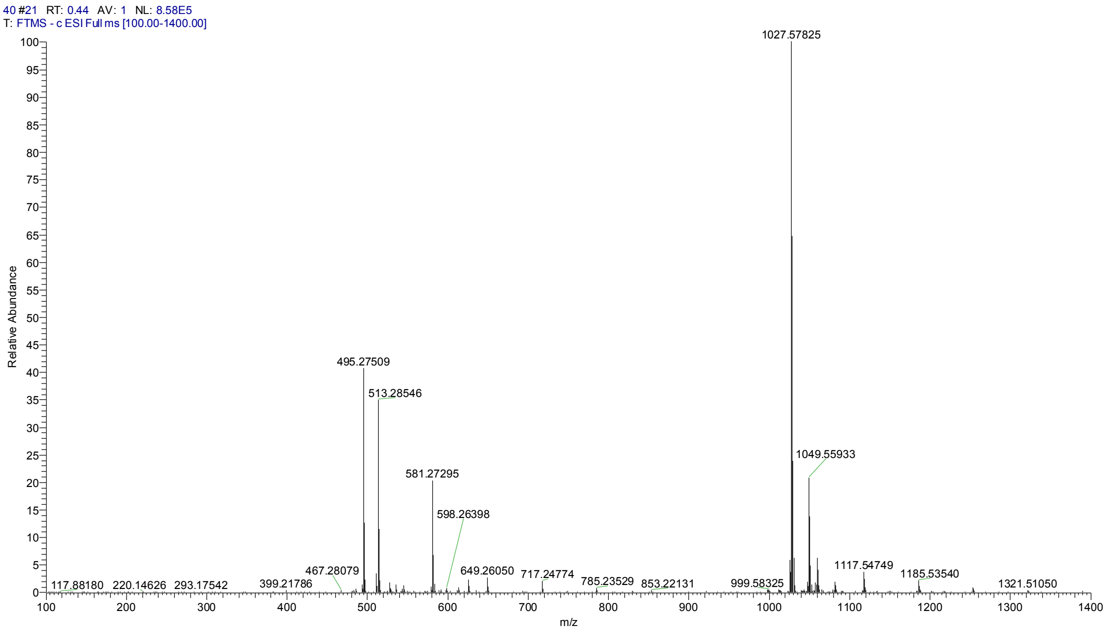

Figure S17. HRESIMS spectrum of **3**

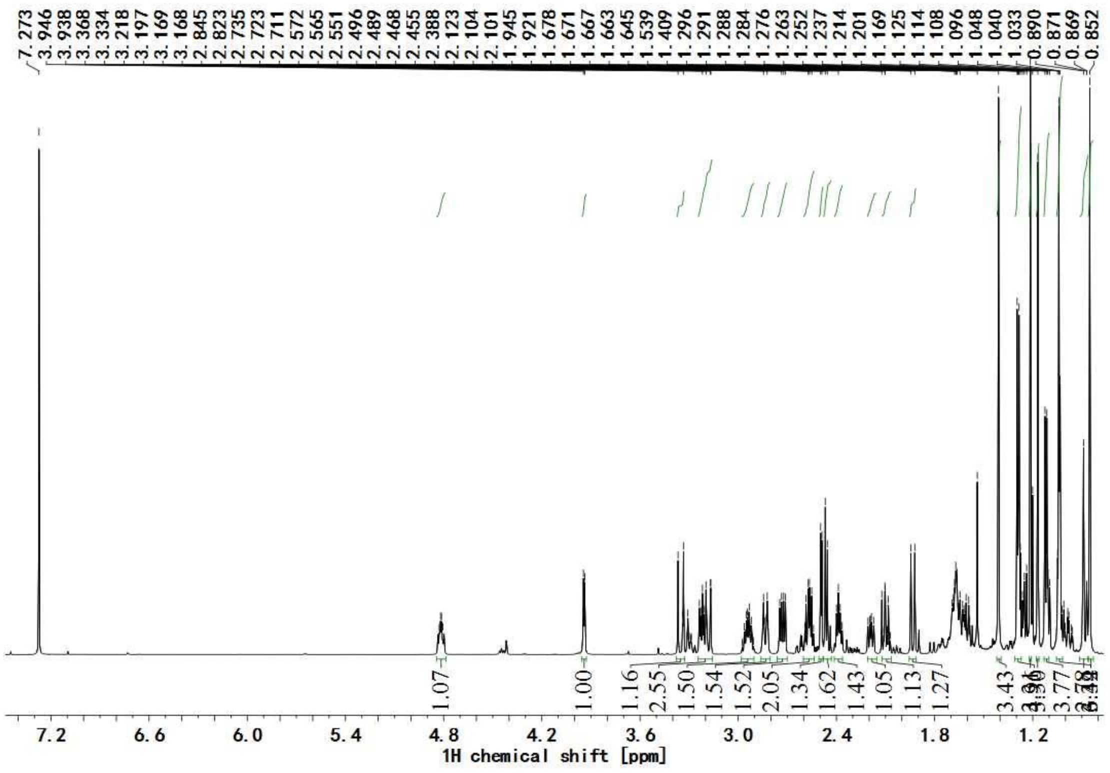

Figure S18. ^{1}H NMR (600 MHz, $CDCl_3$) Spectrum of **3**

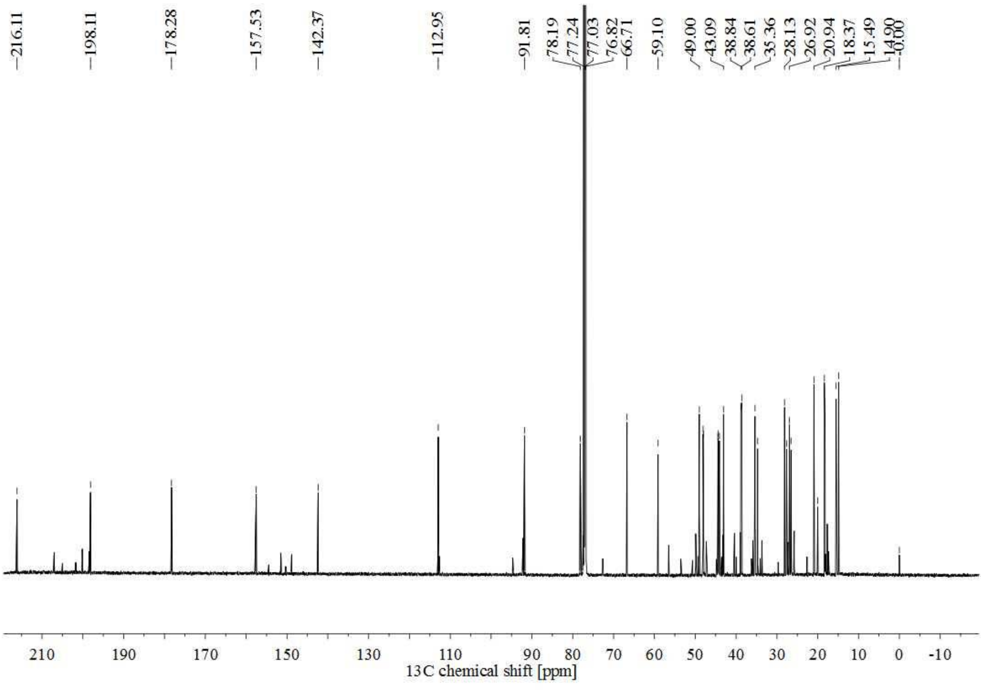

Figure S19. ^{13}C NMR (150 MHz, $CDCl_3$) spectrum of **3**

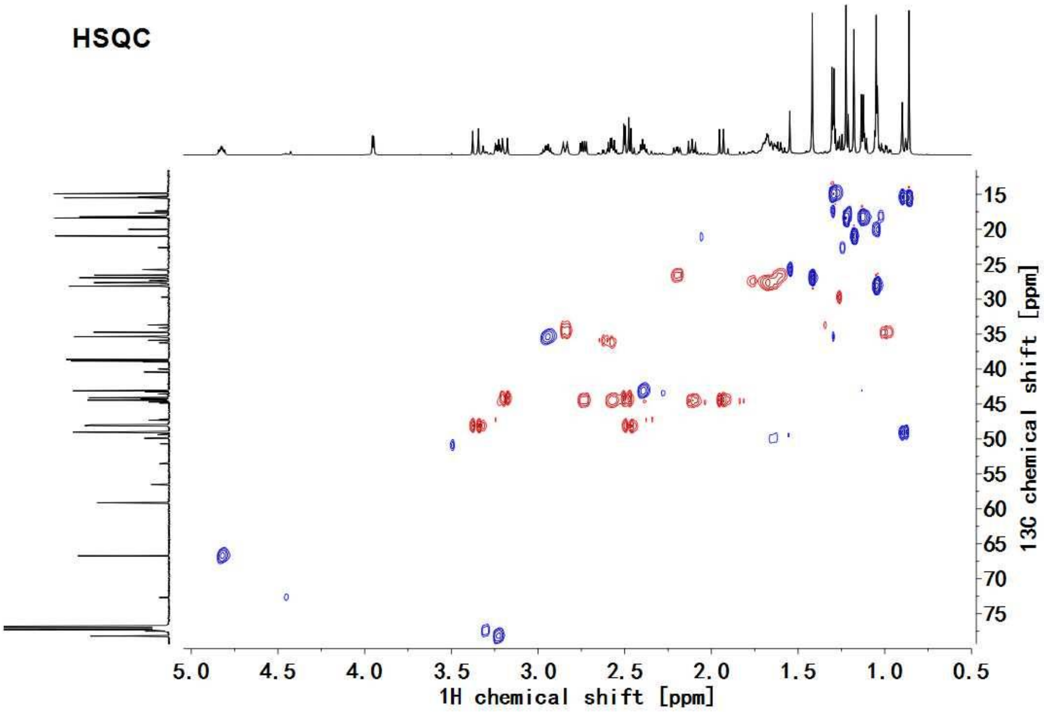

Figure S20. HSQC (600 MHz, $CDCl_3$) spectrum of **3**

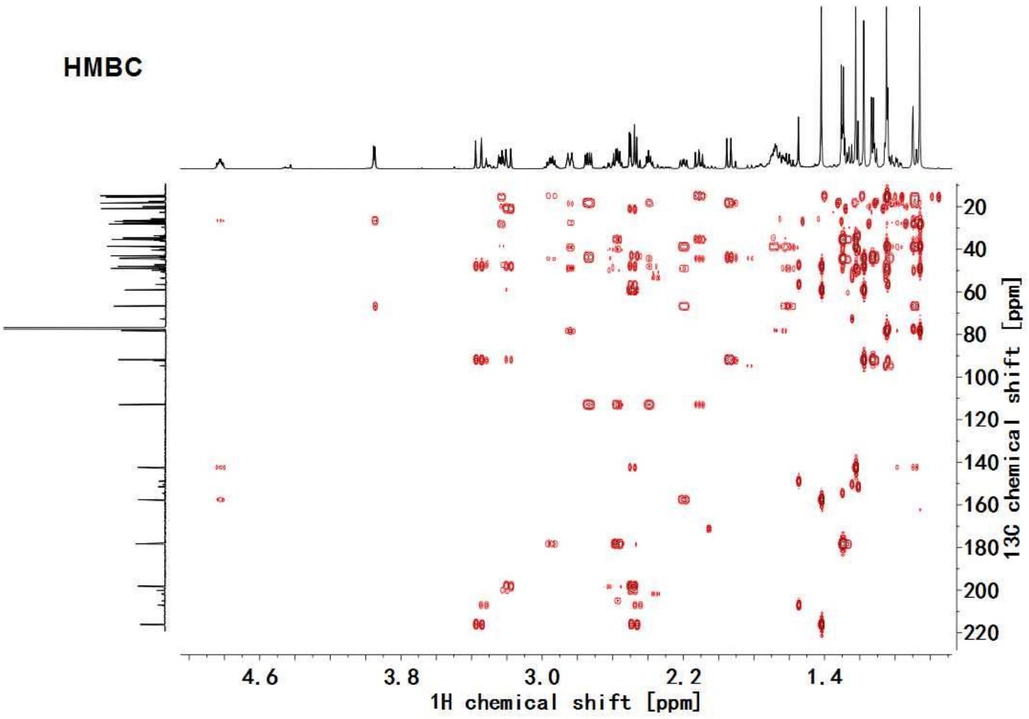

Figure S21. HMBC (600 MHz, $CDCl_3$) spectrum of **3**

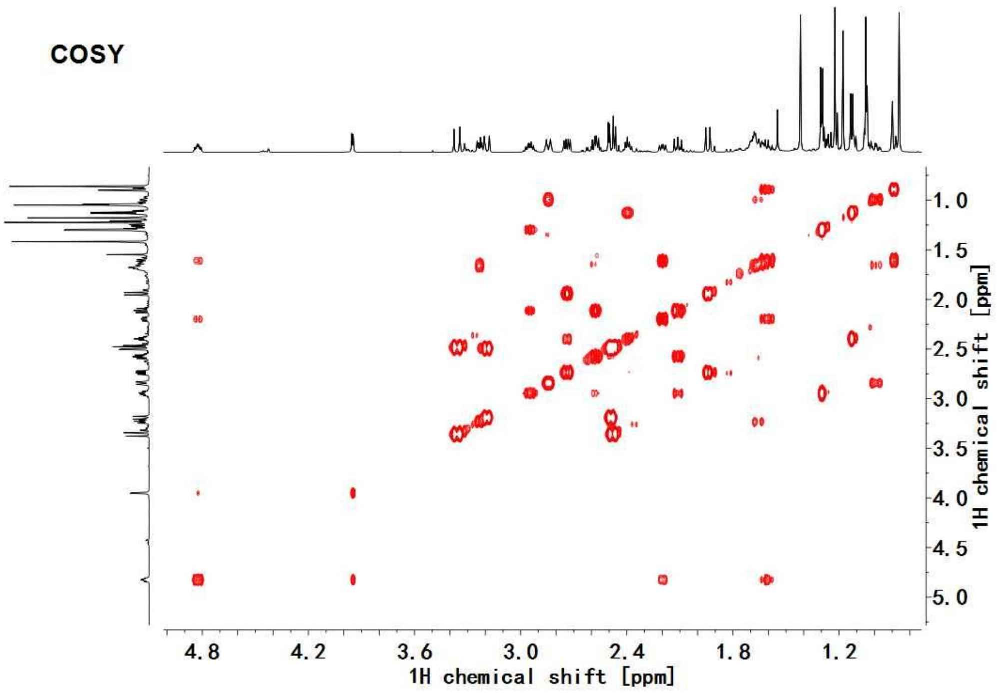

Figure S22. ^{1}H-^{1}H COSY (600 MHz, $CDCl_3$) spectrum of **3**

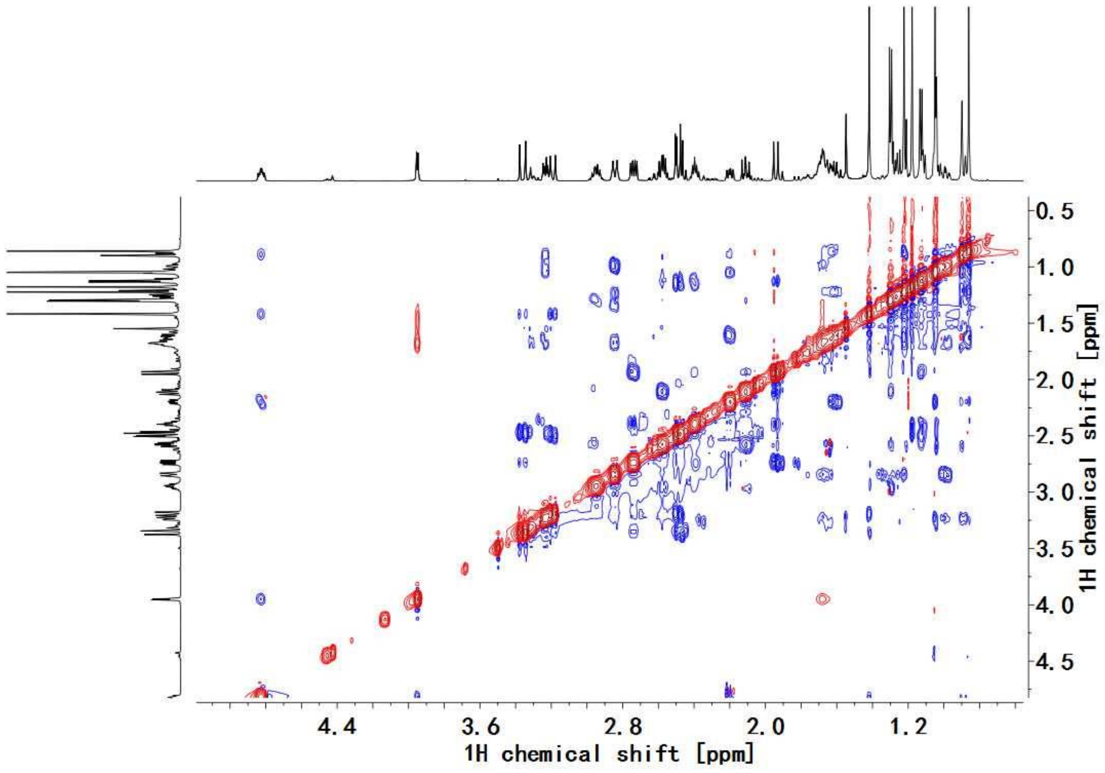

Figure S23. ROESY (600 MHz, $CDCl_3$) spectrum of **3**

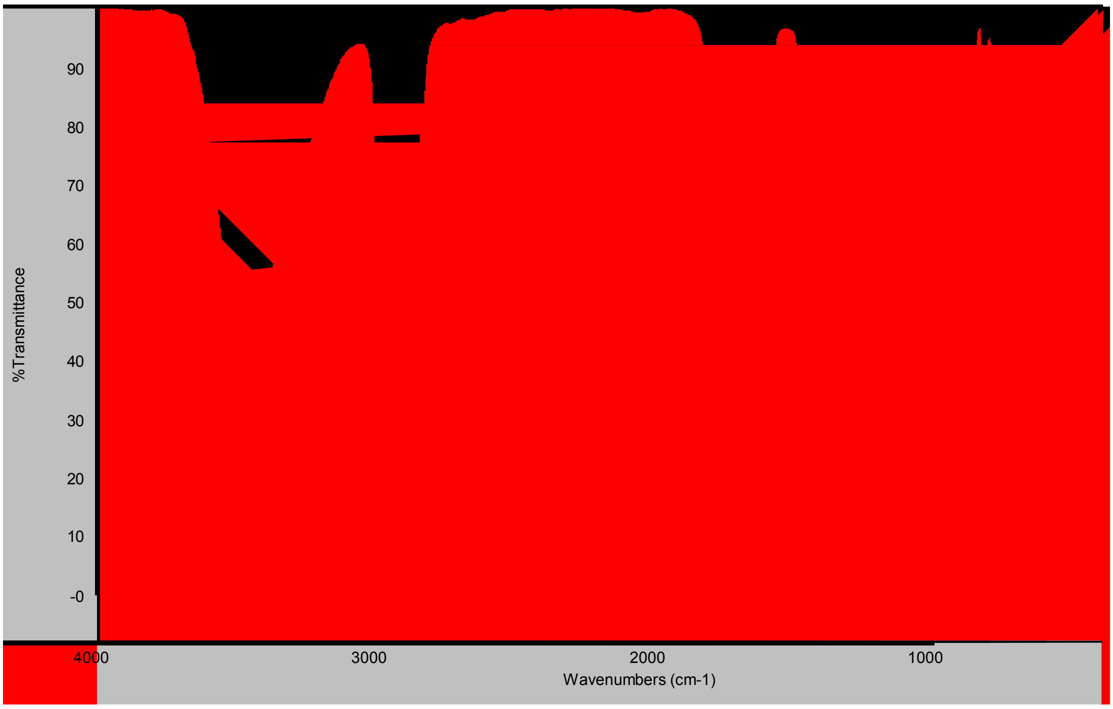

Figure S24. IR spectrum of 3

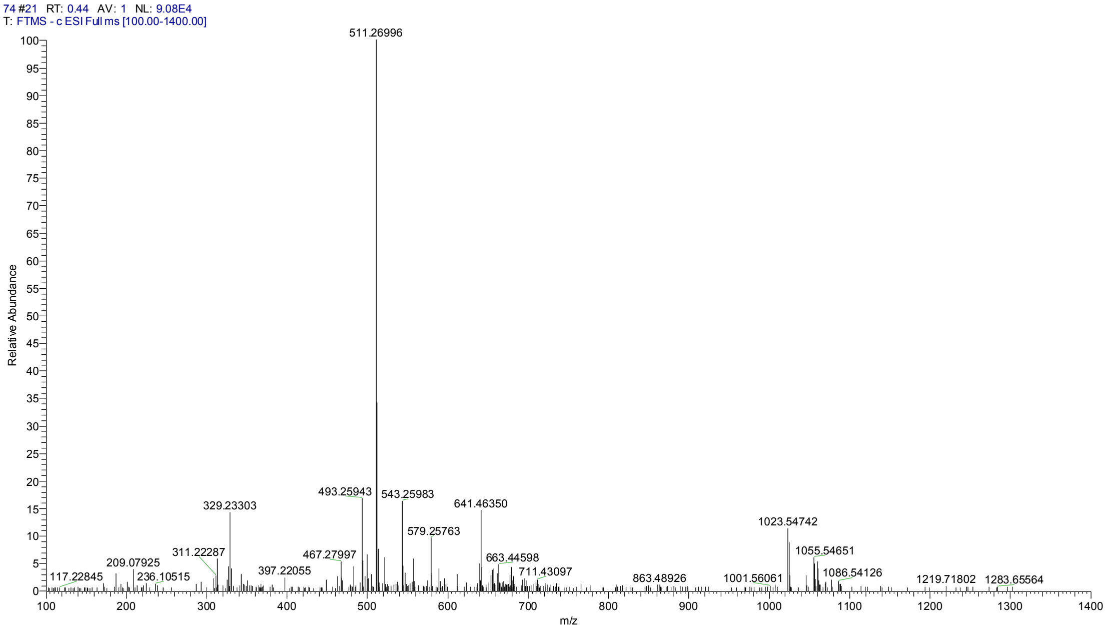

Figure S25. HRESIMS spectrum of **4**

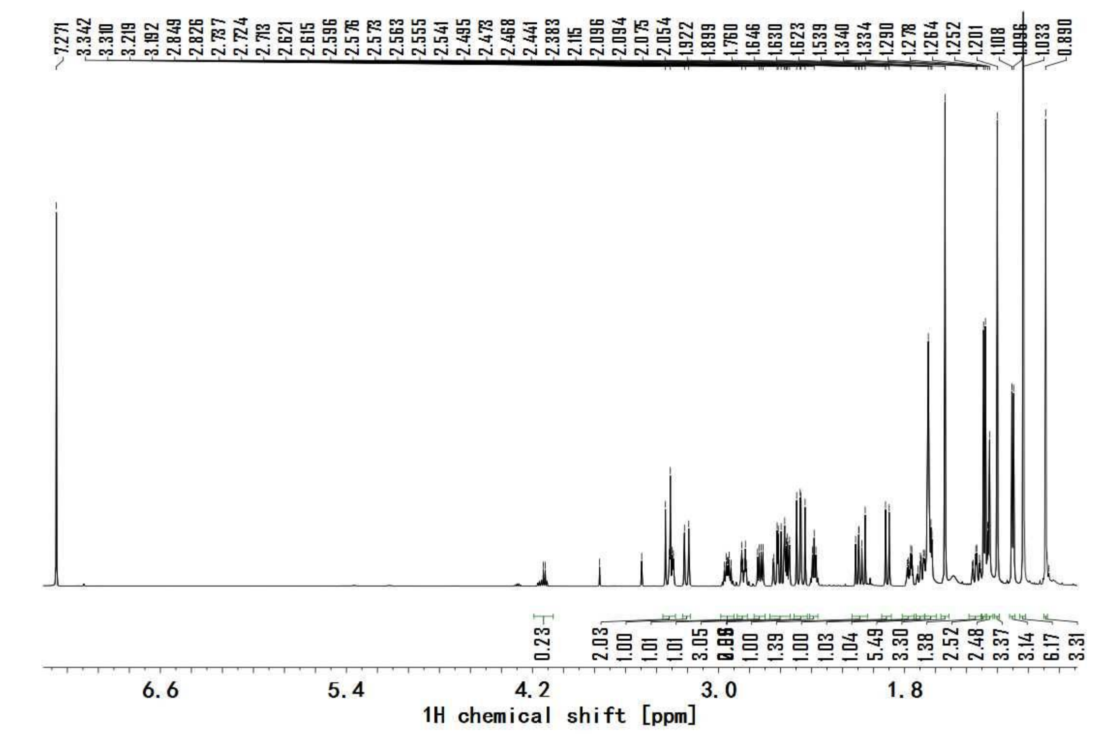

Figure S26. ^{1}H NMR (600 MHz, $CDCl_3$) Spectrum of **4**

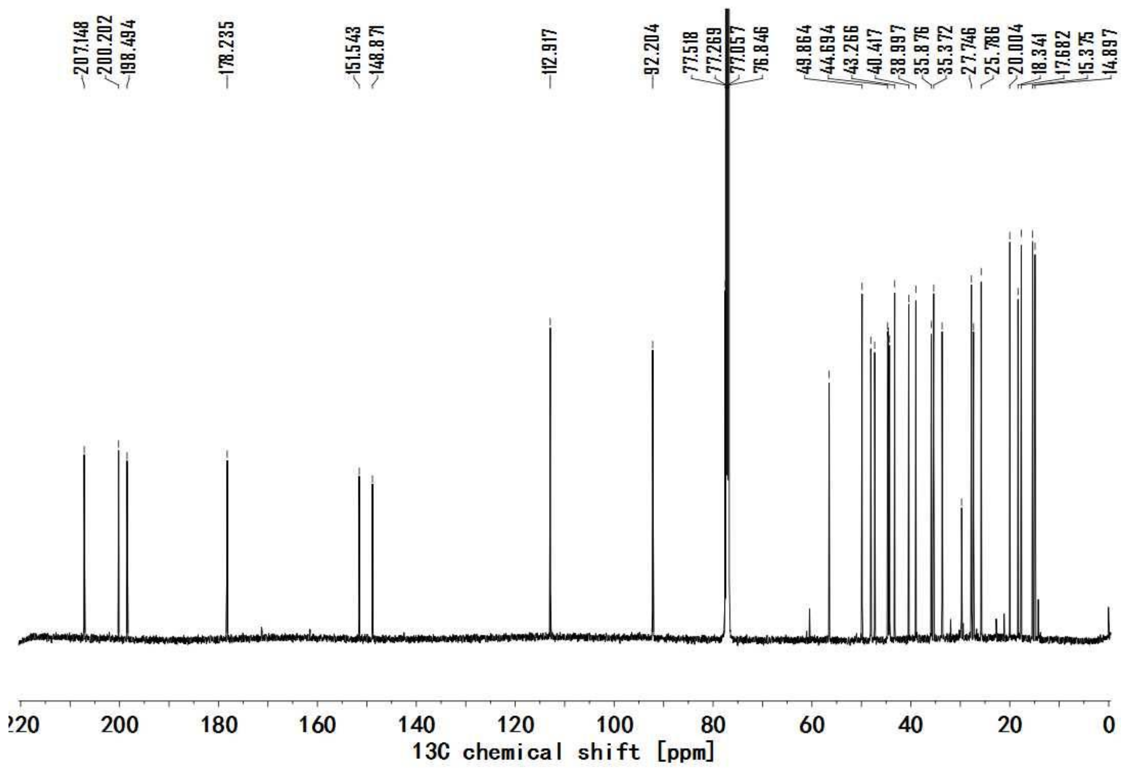

Figure S27. ^{13}C NMR (150 MHz, $CDCl_3$) spectrum of **4**

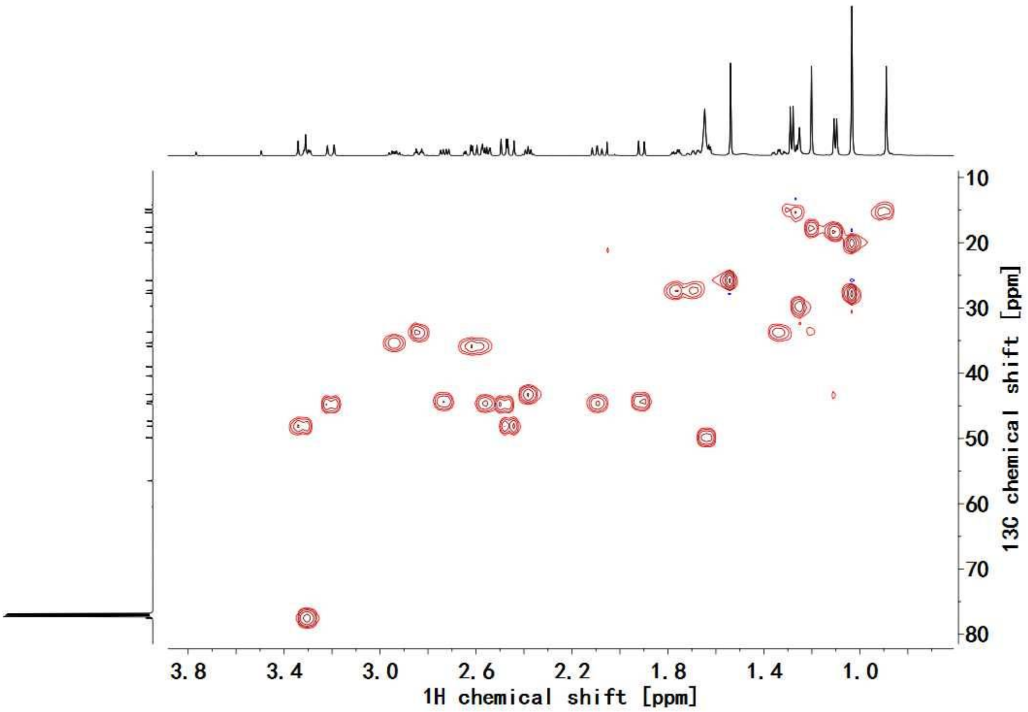

Figure S28. HSQC (600 MHz, $CDCl_3$) spectrum of **4**

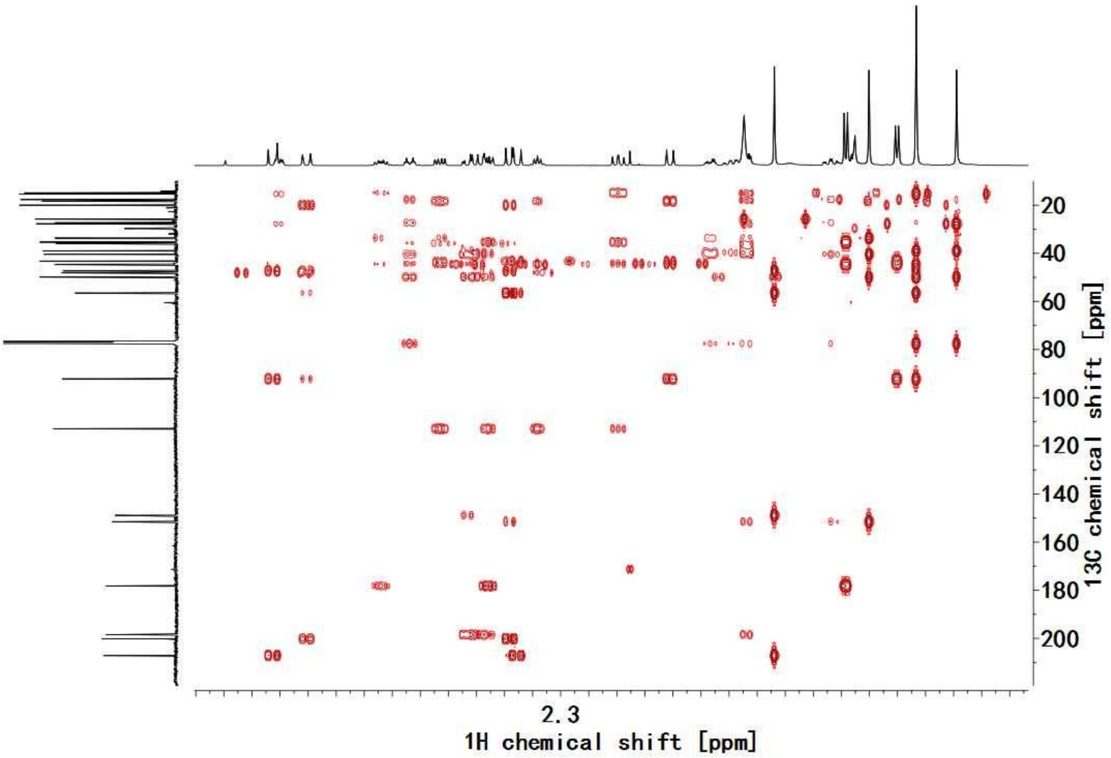

Figure S29. HMBC (600 MHz, $CDCl_3$) spectrum of **4**

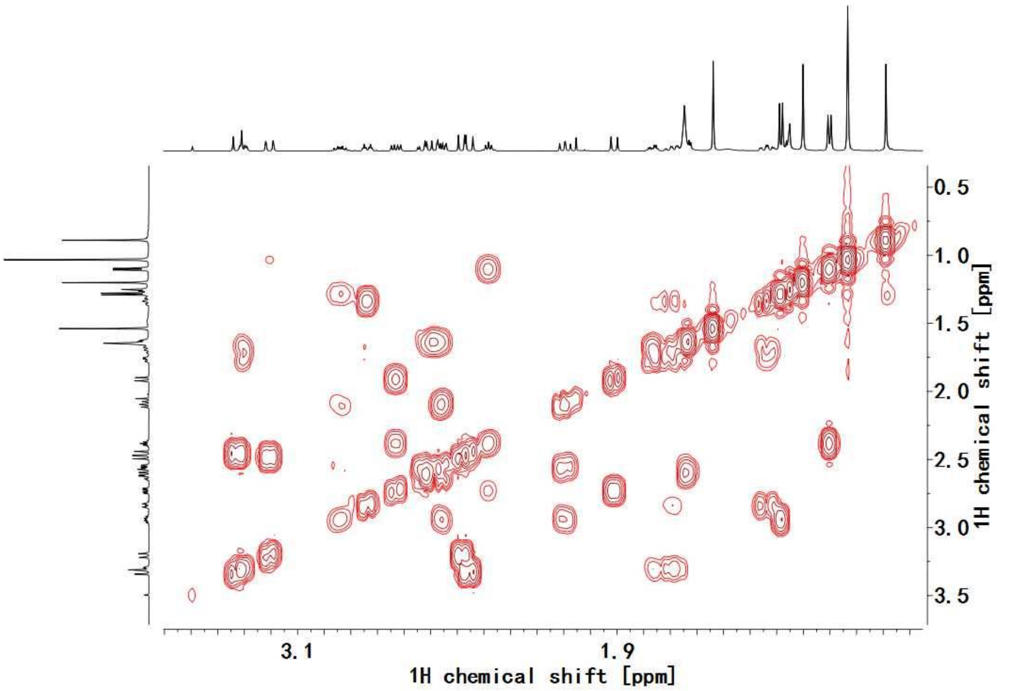

Figure S30. ^{1}H-^{1}H COSY (600 MHz, $CDCl_3$) spectrum of **4**

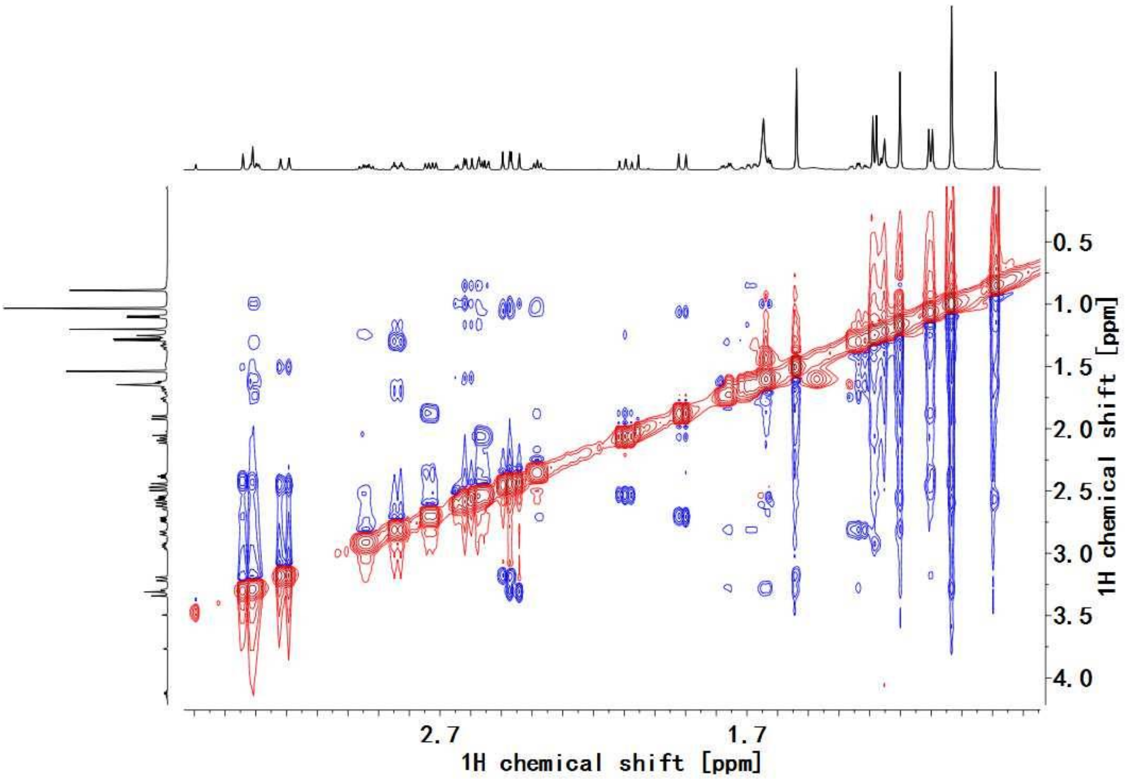

Figure S31. ROESY (600 MHz, $CDCl_3$) spectrum of **4**

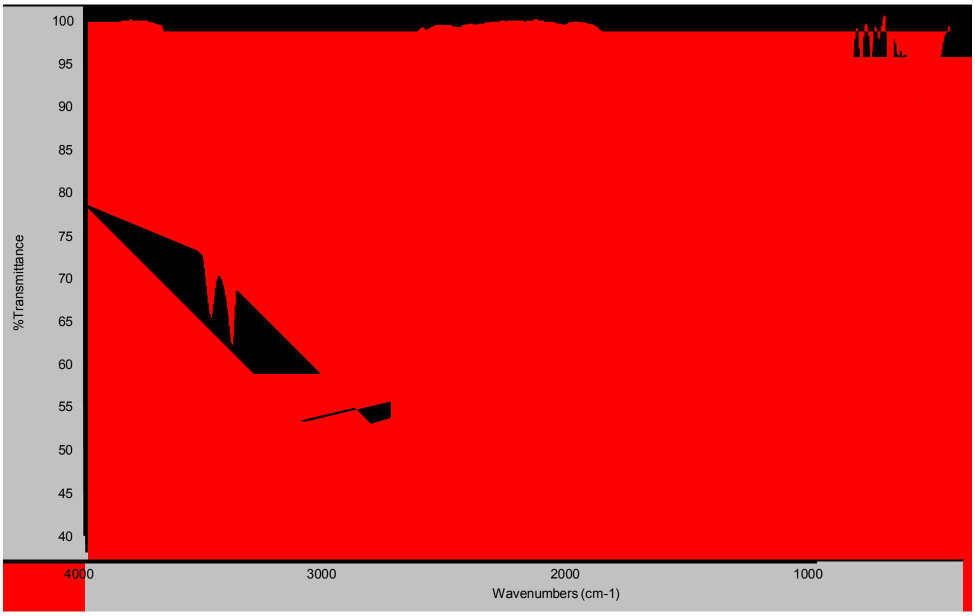

Figure S32. IR spectrum of **4**

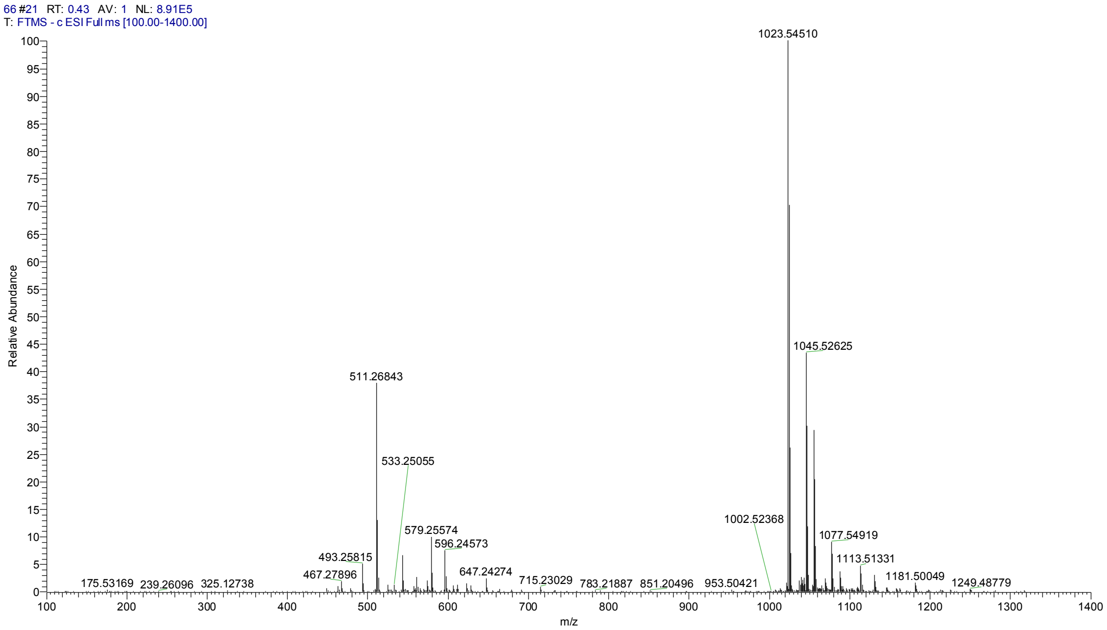

Figure S33. HRESIMS spectrum of **5**

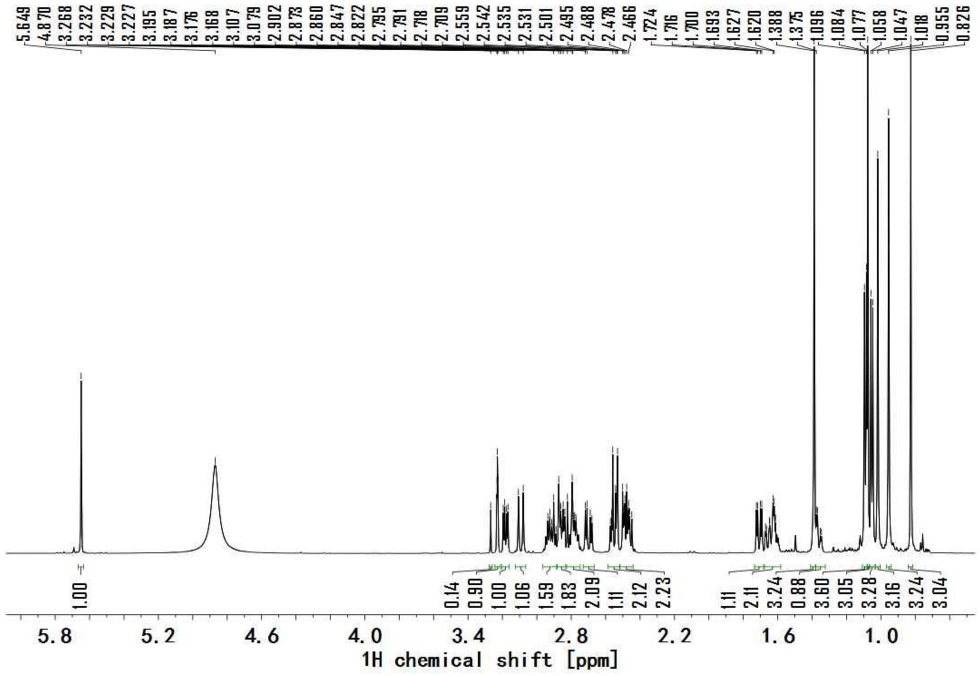

Figure S34. ^{1}H NMR (600 MHz, CD_3OD) spectrum of **5**

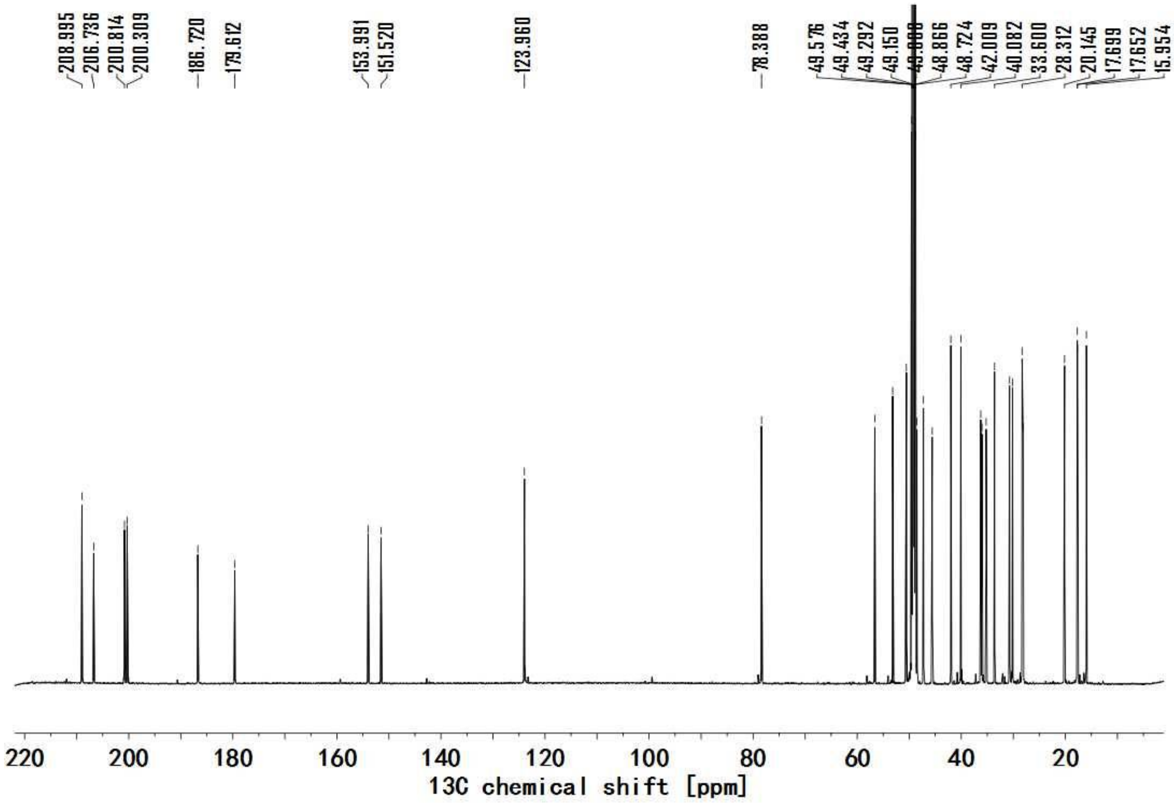

Figure S35. ^{13}C NMR (150 MHz, CD_3OD) spectrum of **5**

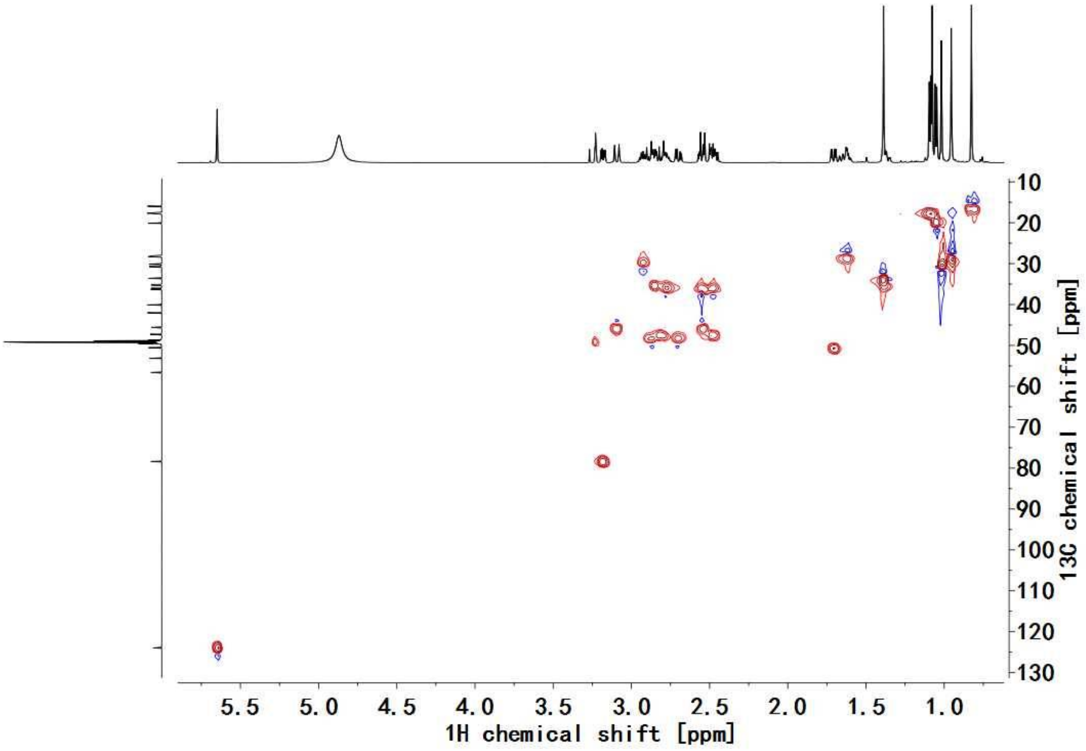

Figure S36. HSQC (600 MHz, CD_3OD) spectrum of **5**

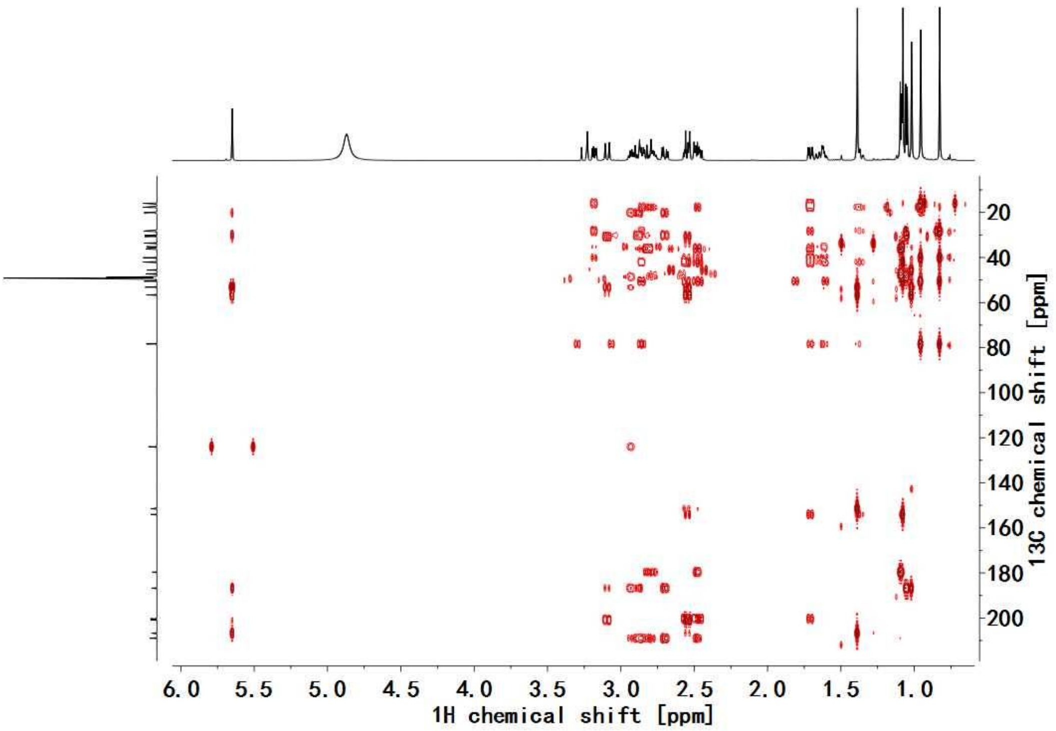

Figure S37. HMBC (600 MHz, CD_3OD) spectrum of **5**

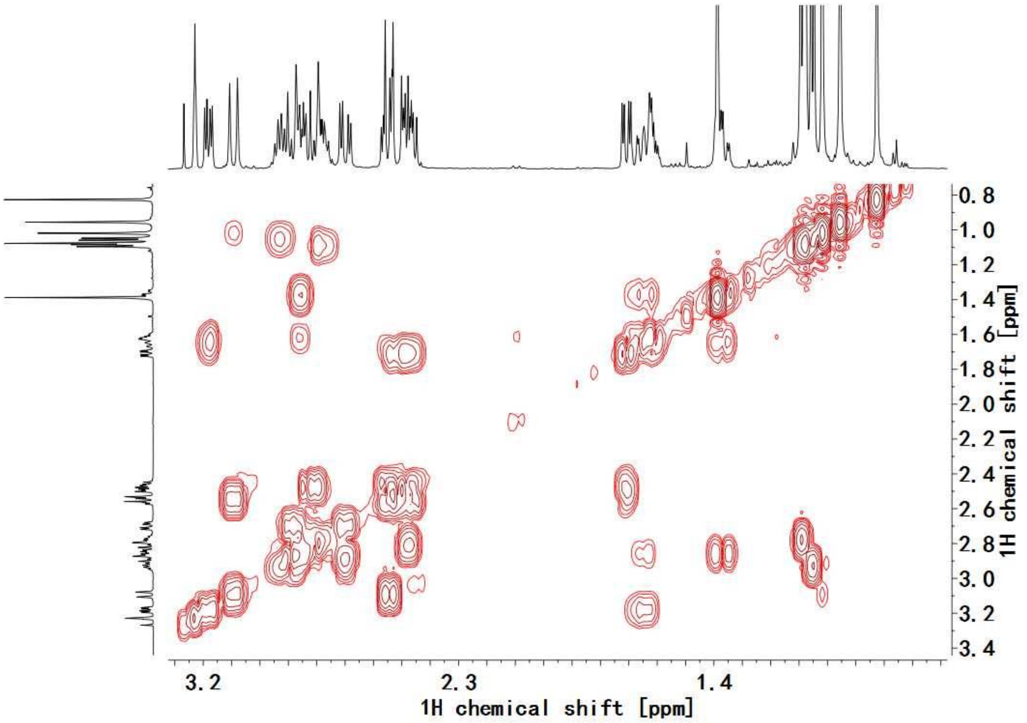

Figure S38. ^{1}H-^{1}H COSY (600 MHz, CD_3OD) spectrum of **5**

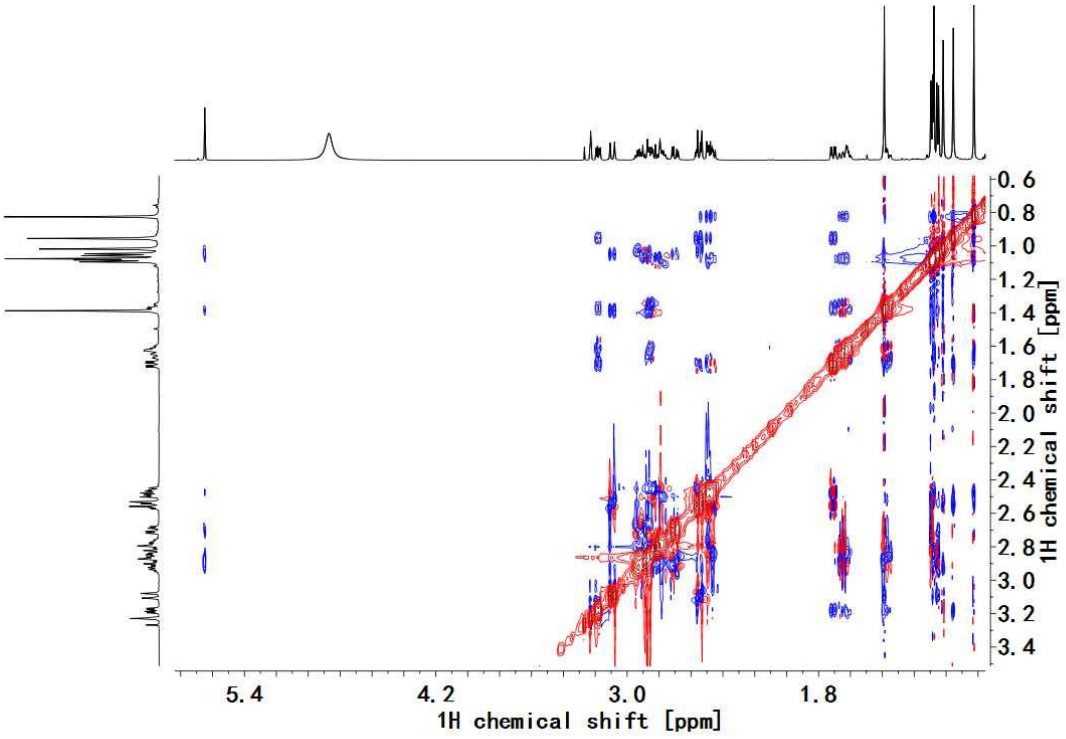

Figure S39. ROESY (600 MHz, CD_3OD) spectrum of **5**

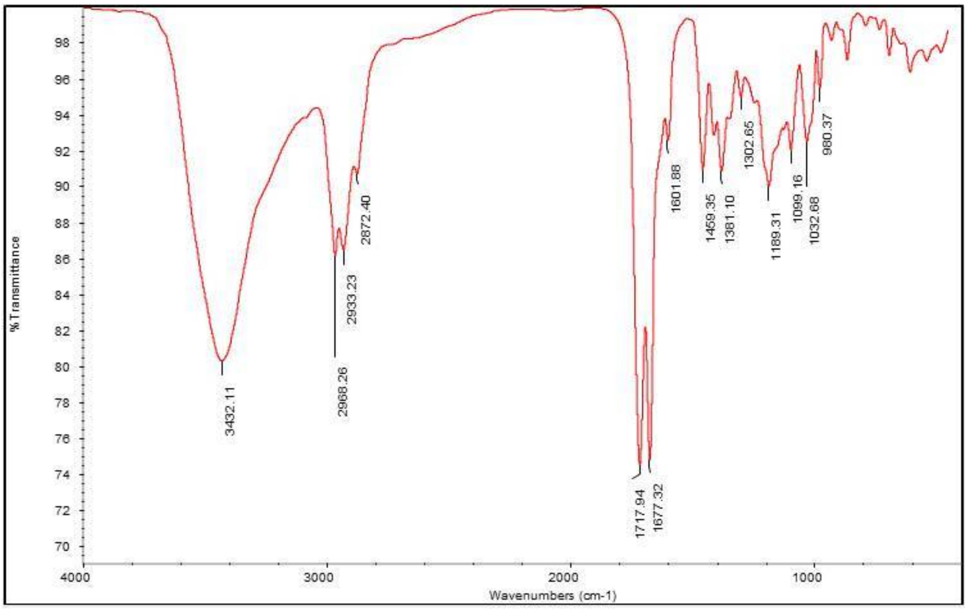

Figure S40. IR spectrum of **5**

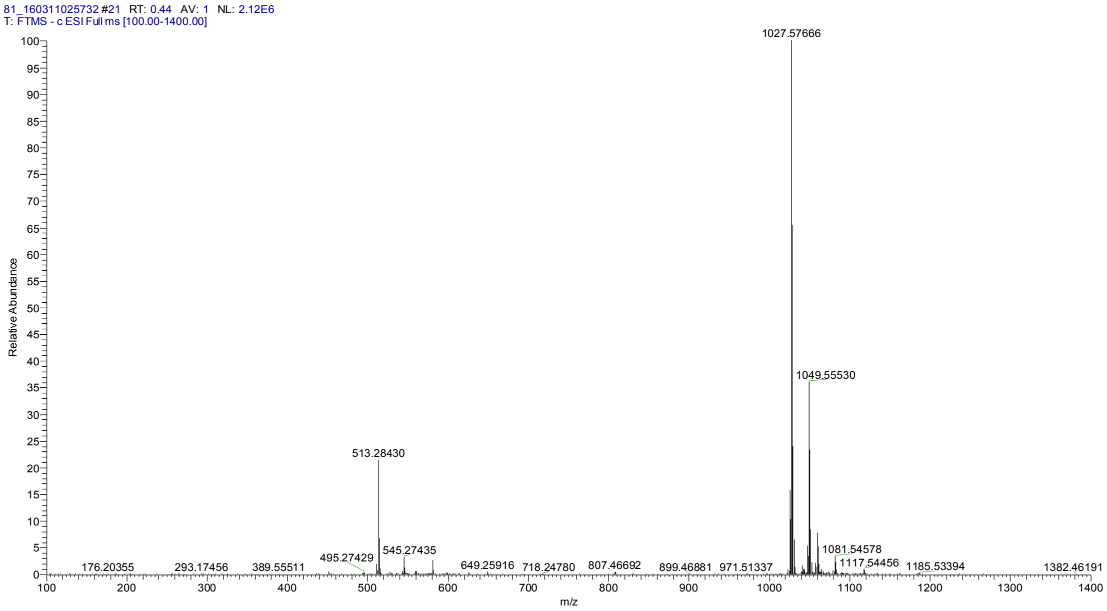

Figure S41. HRESIMS spectrum of **6**

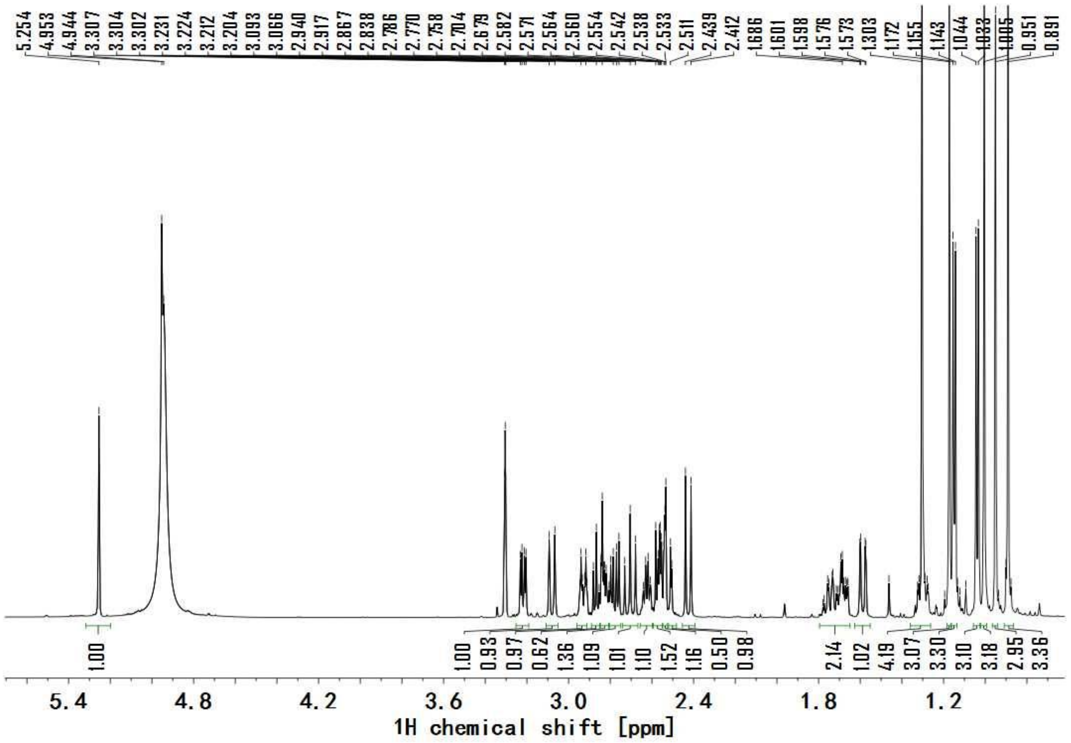

Figure S42. ^{1}H NMR (600 MHz, CD_3OD) spectrum of **6**

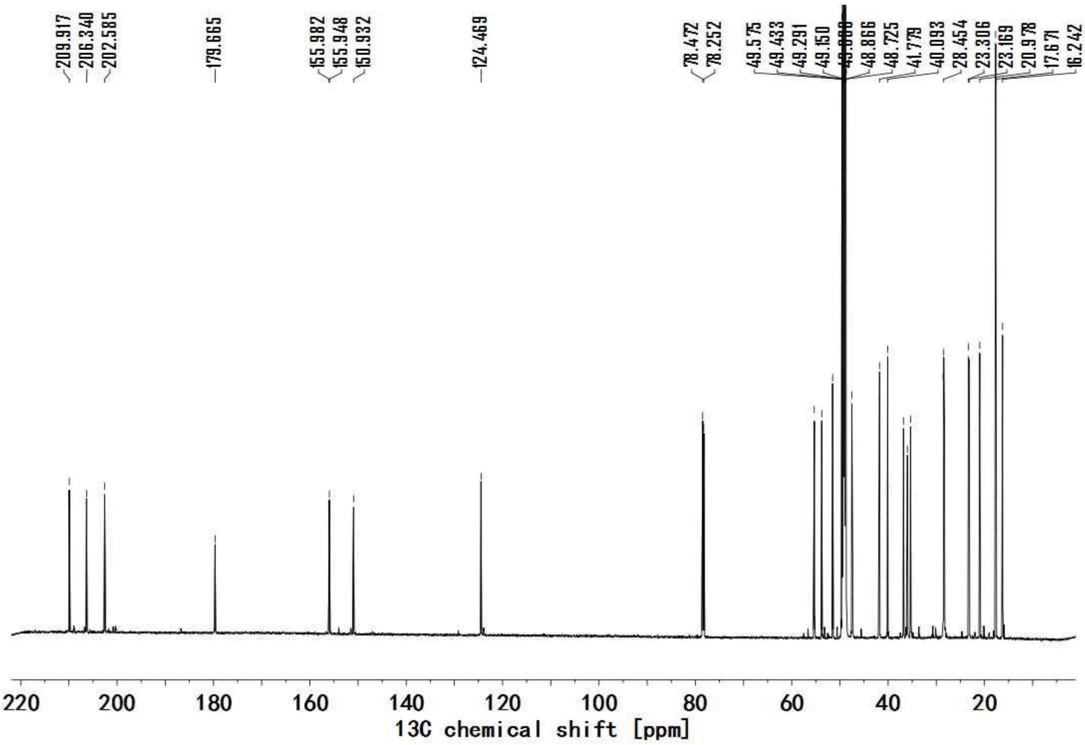

Figure S43. ^{13}C NMR (150 MHz, CD_3OD) spectrum of **6**

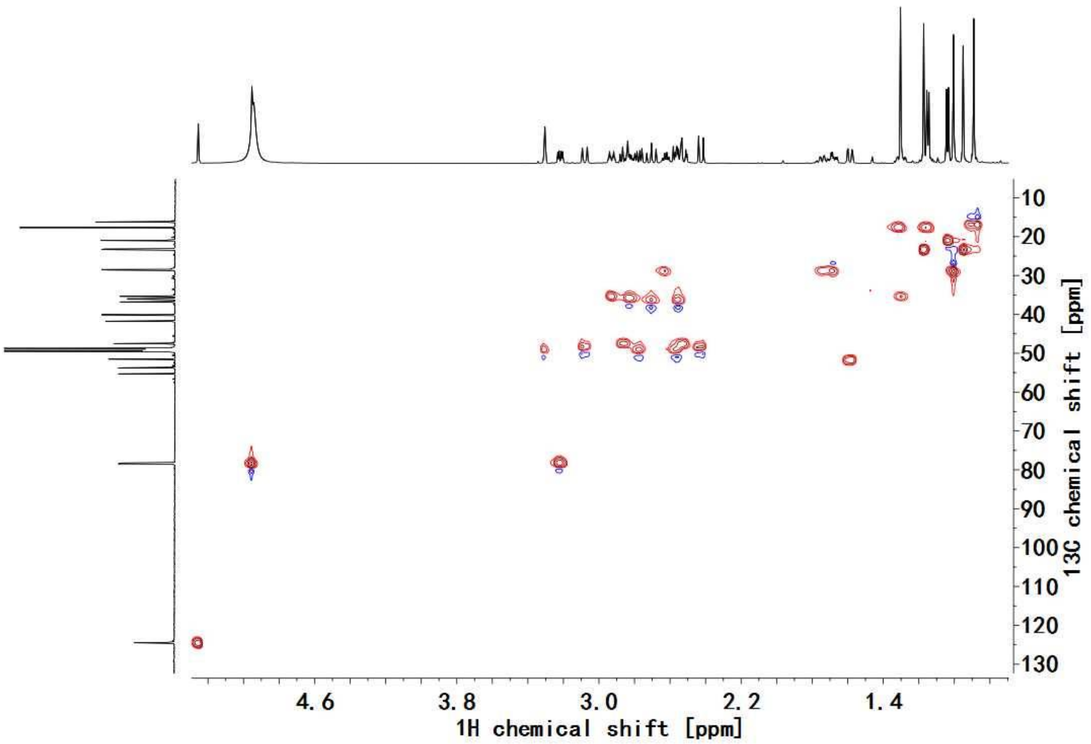

Figure S44. HSQC (600 MHz, CD_3OD) spectrum of **6**

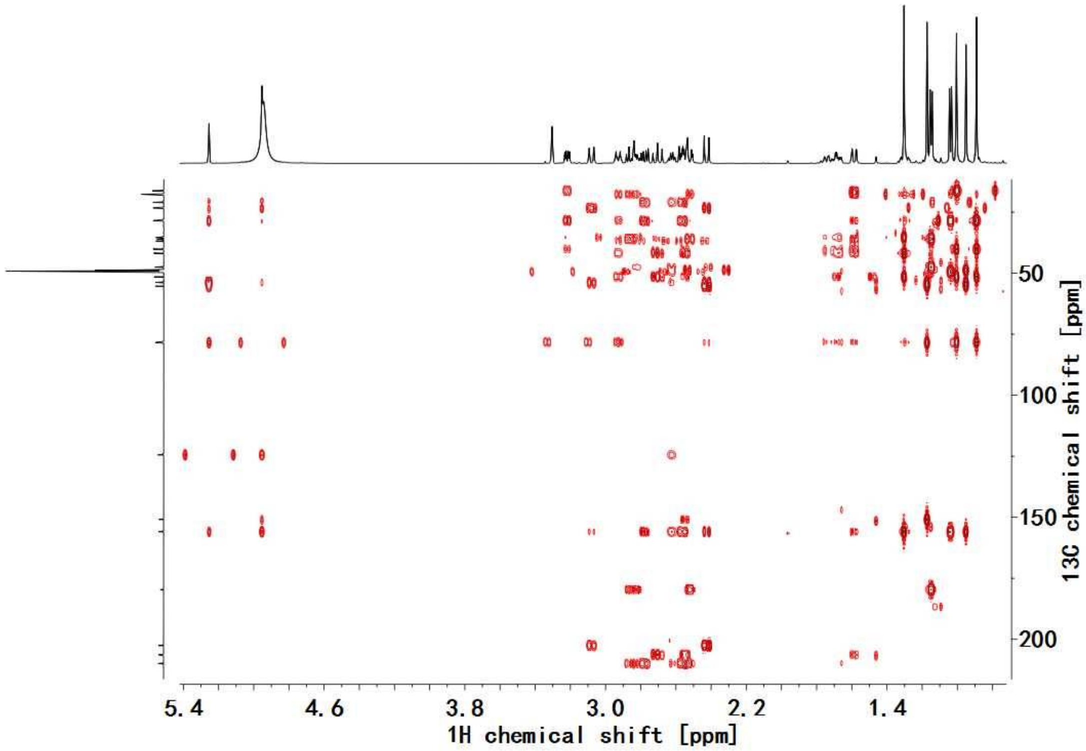

Figure S45. HMBC (600 MHz, CD_3OD) spectrum of **6**

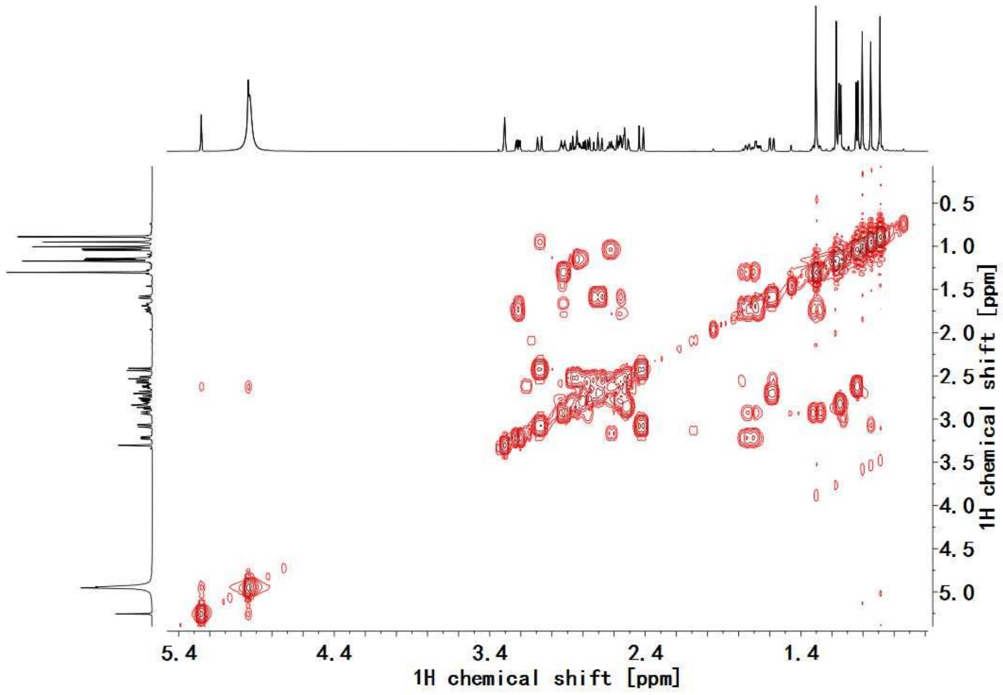

Figure S46. ^{1}H-^{1}H COSY (600 MHz, CD_3OD) spectrum of **6**

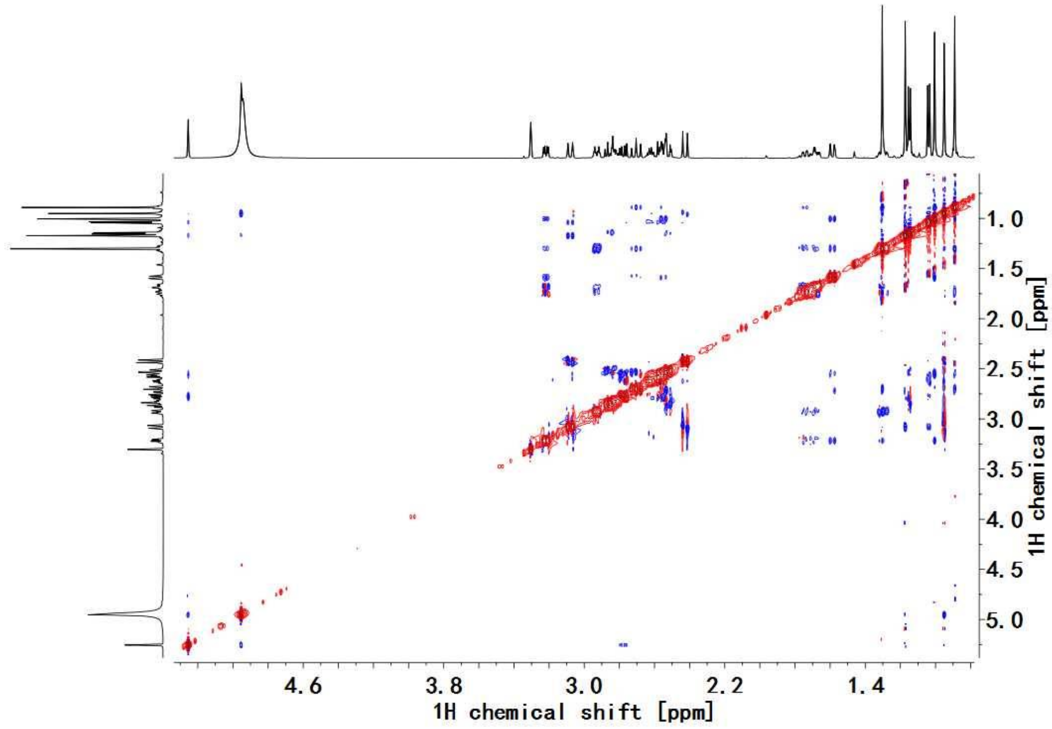

Figure S47. ROESY (600 MHz, CD_3OD) spectrum of **6**

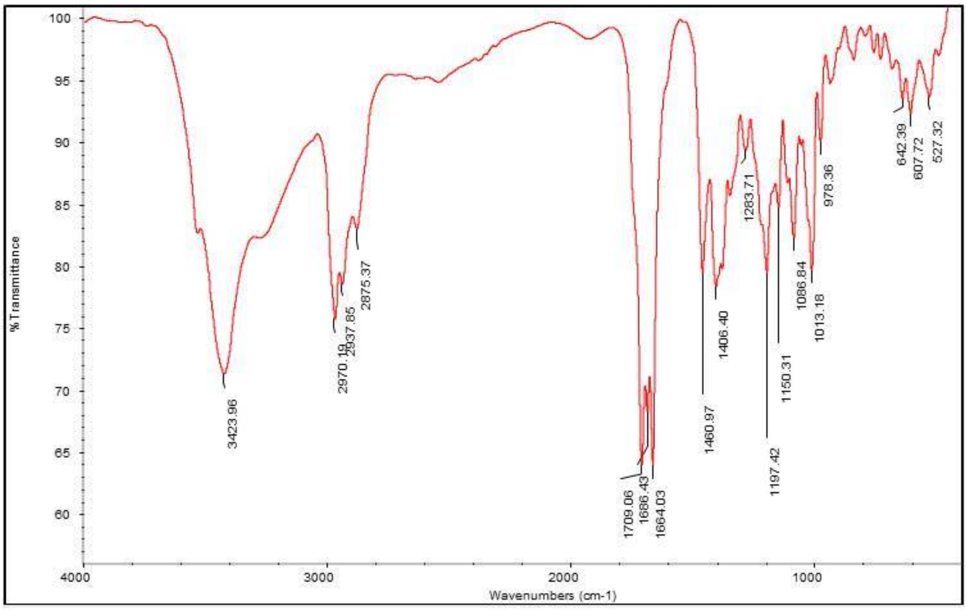

Figure S48. IR spectrum of **6**

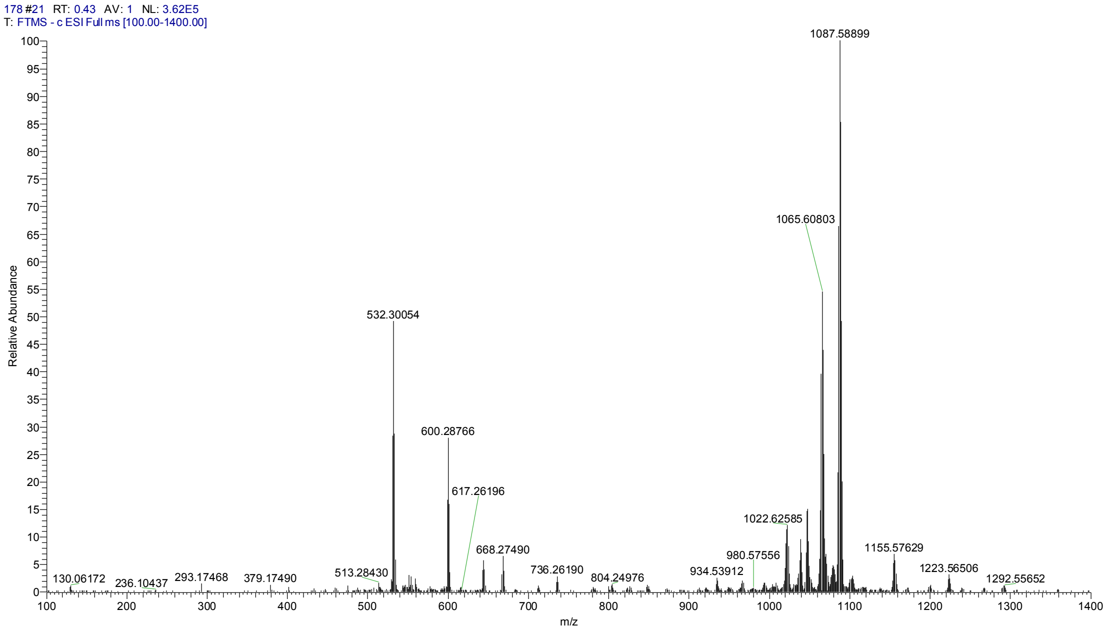

Figure S49. HRESIMS spectrum of **7**

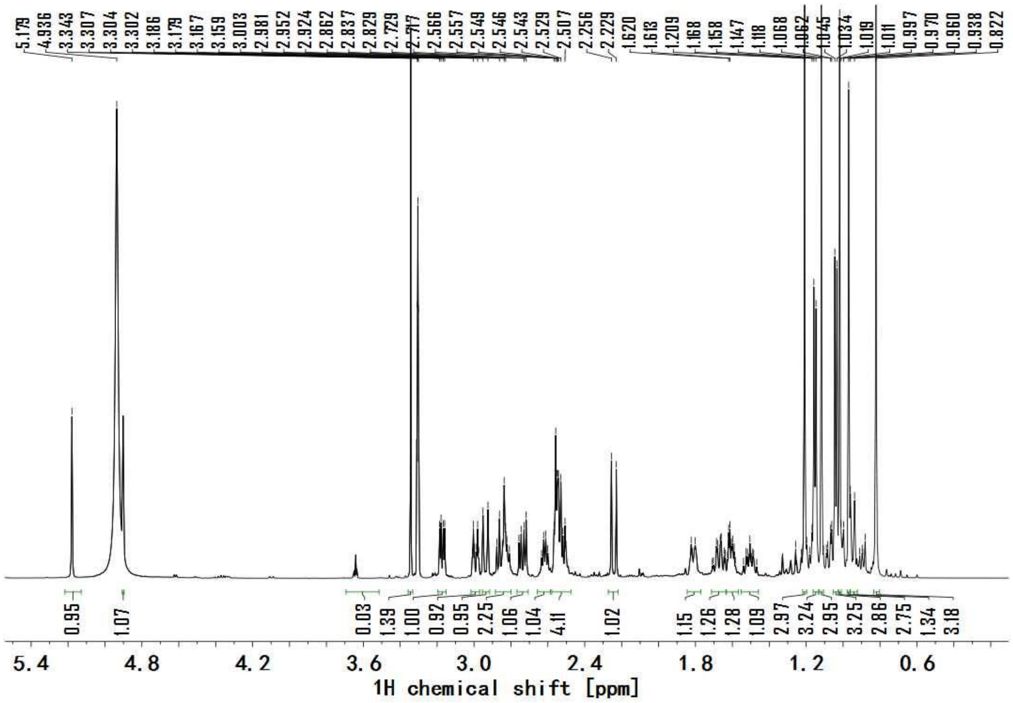

Figure S50. ^{1}H NMR (600 MHz, CD_3OD) spectrum of **7**

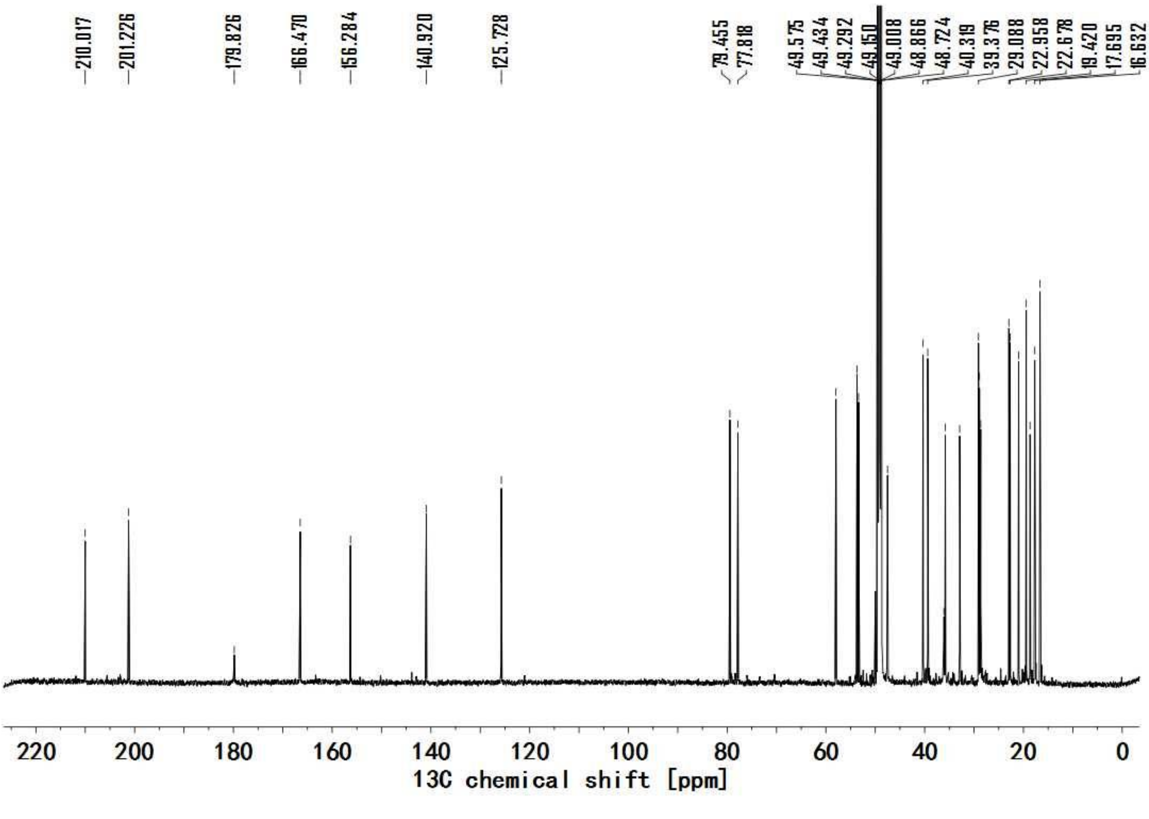

Figure S51. ^{13}C NMR (150 MHz, CD_3OD) spectrum of **7**

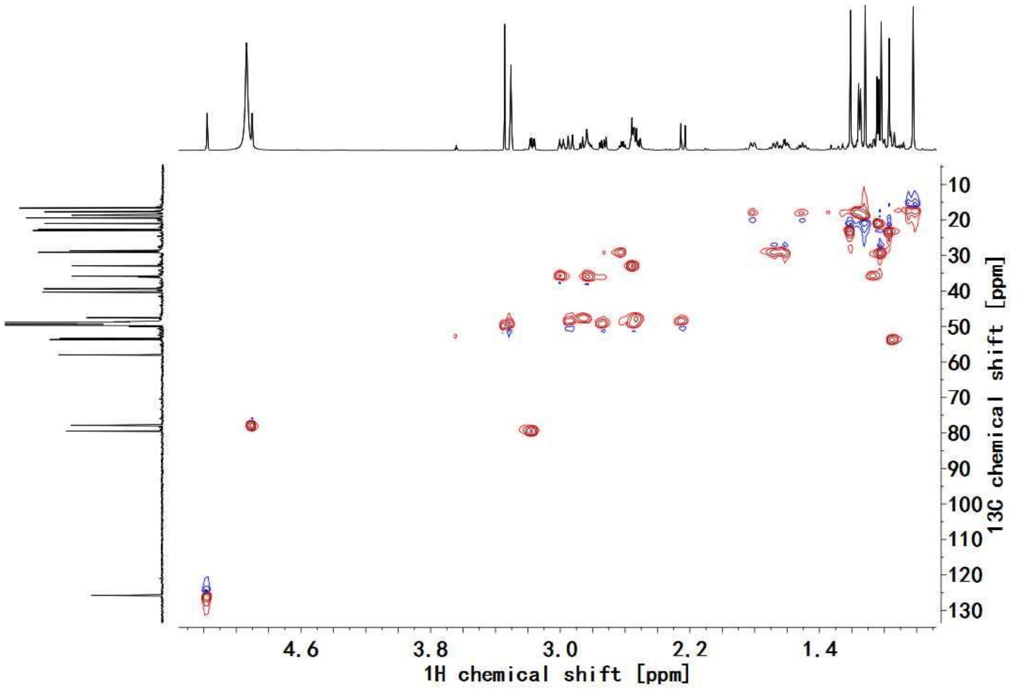

Figure S52. HSQC (600 MHz, CD_3OD) spectrum of **7**

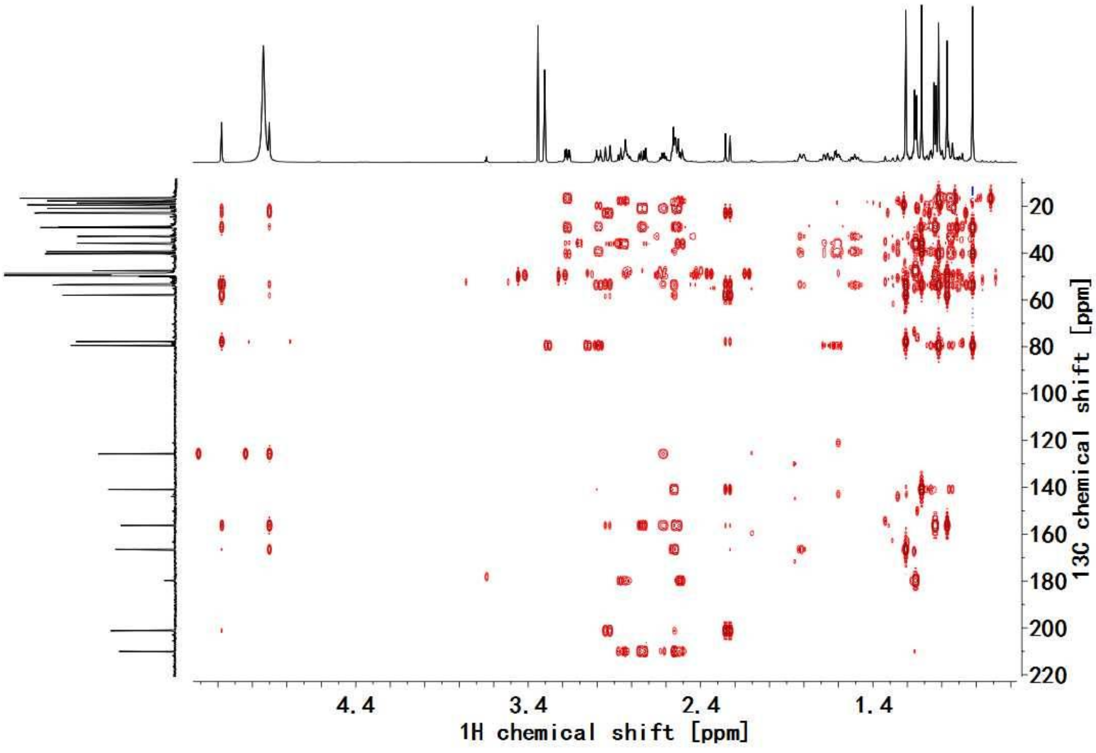

Figure S53. HMBC (600 MHz, CD_3OD) spectrum of **7**

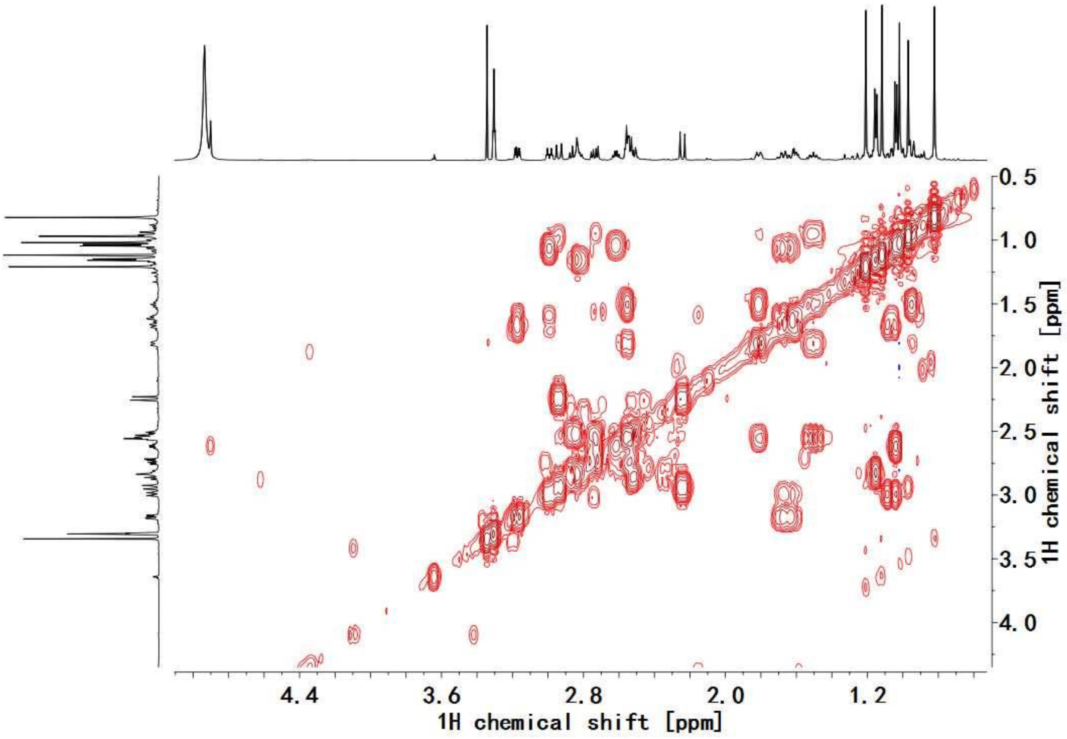

Figure S54. ^1H-^1H COSY (600 MHz, CD_3OD) spectrum of **7**

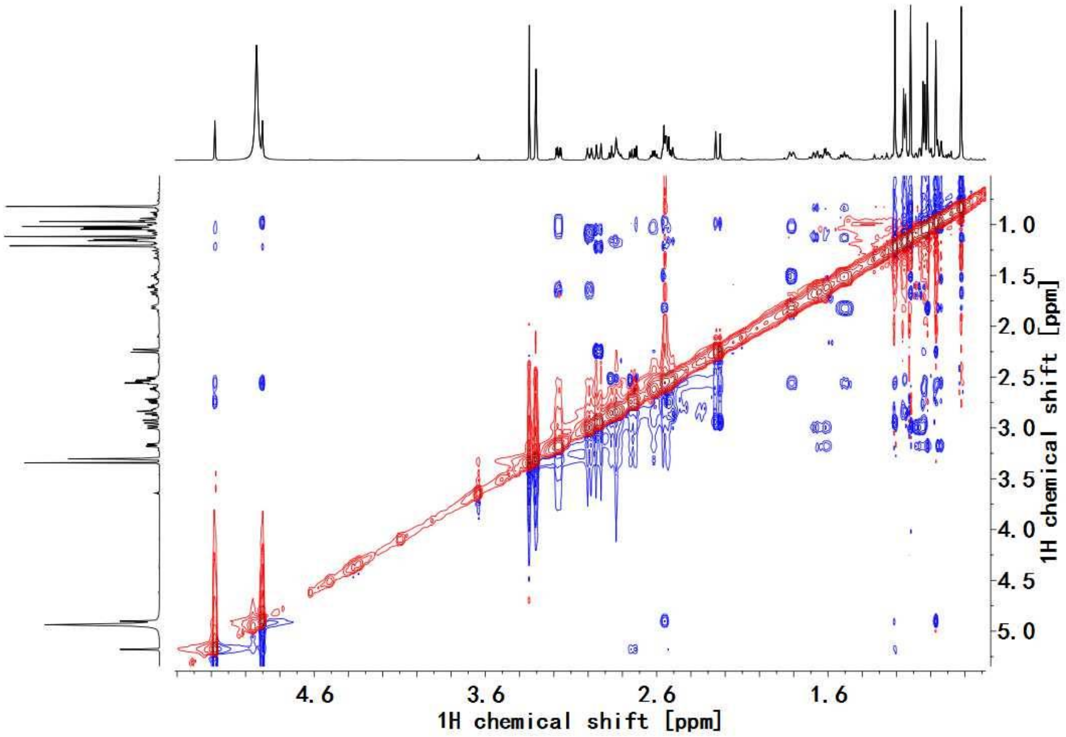

Figure S55. ROESY (600 MHz, CD_3OD) spectrum **7**

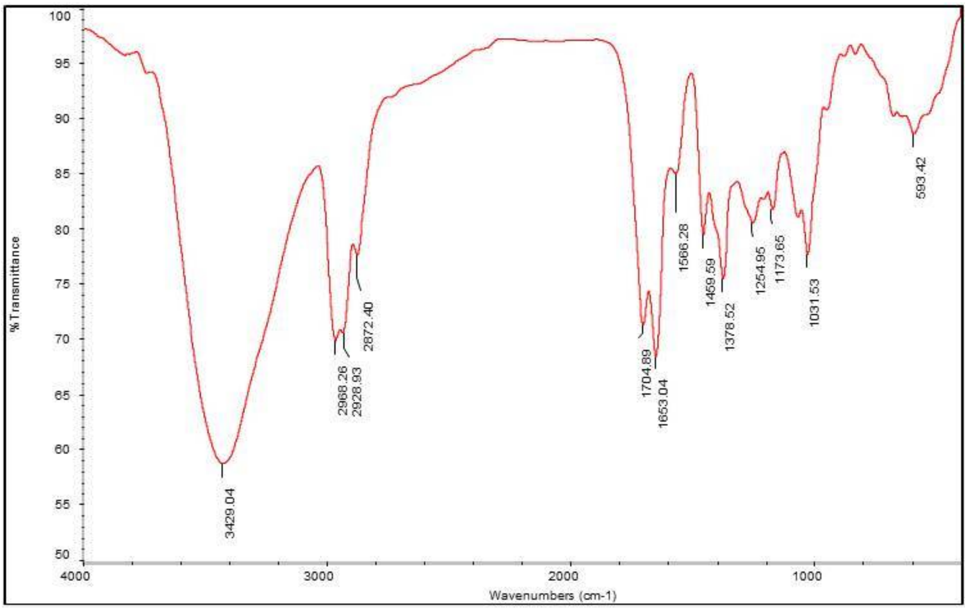

Figure S56. IR spectrum of **7**

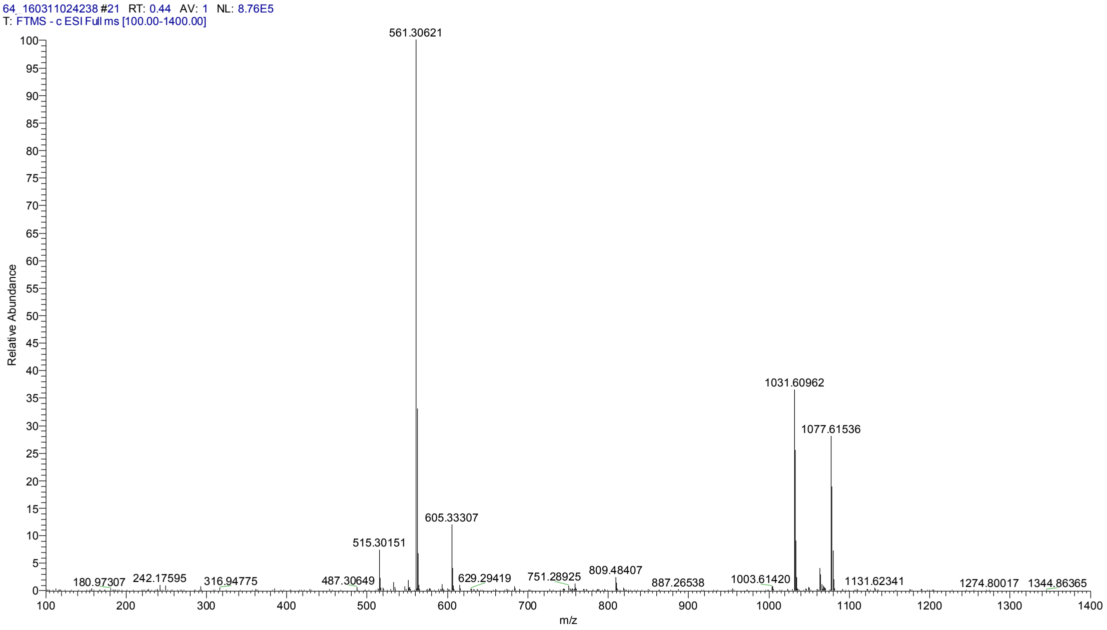

Figure S57. HRESIMS spectrum of **8**

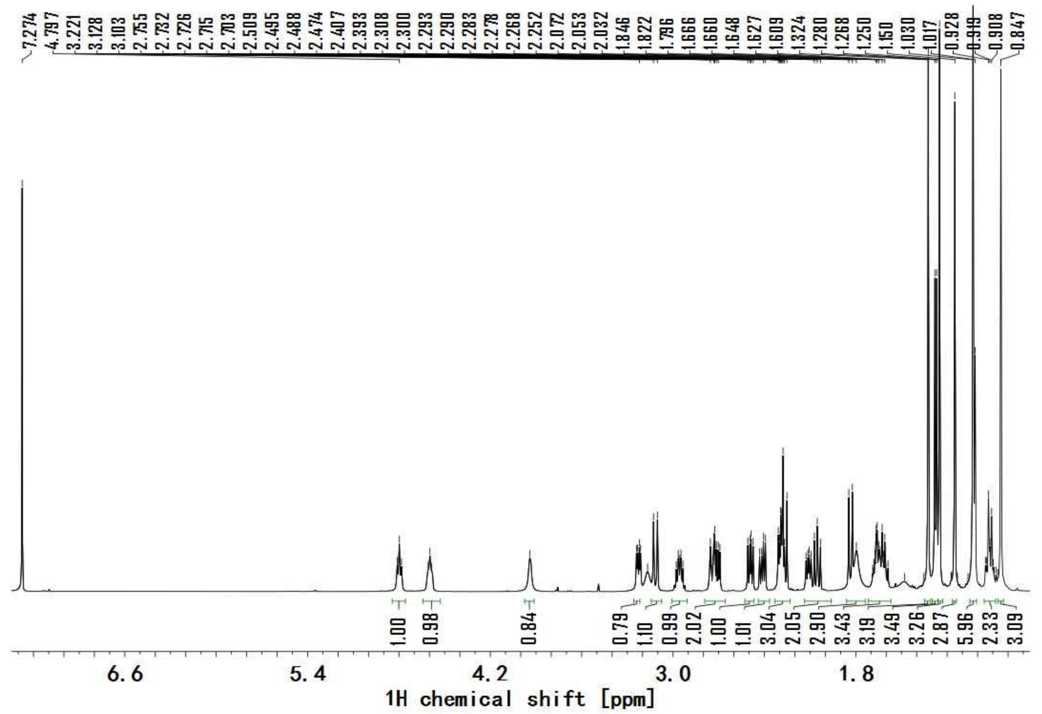

Figure S58. ^{1}H NMR (600 MHz, $CDCl_3$) Spectrum of **8**

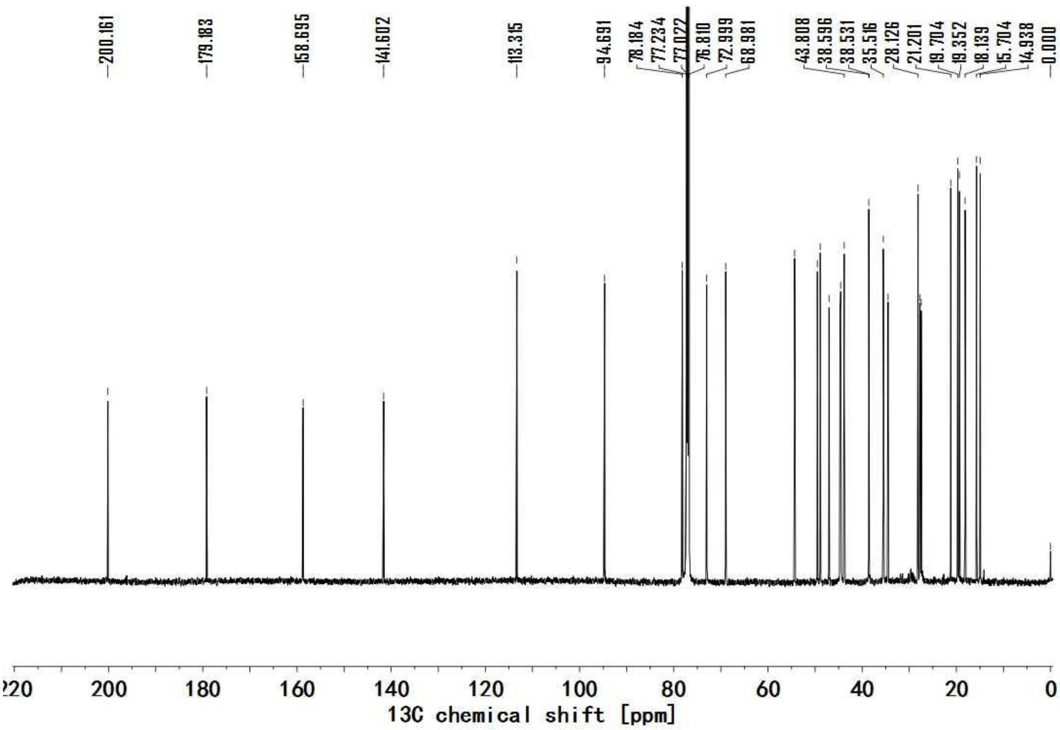

Figure S59. ^{13}C NMR (150 MHz, $CDCl_3$) spectrum of **8**

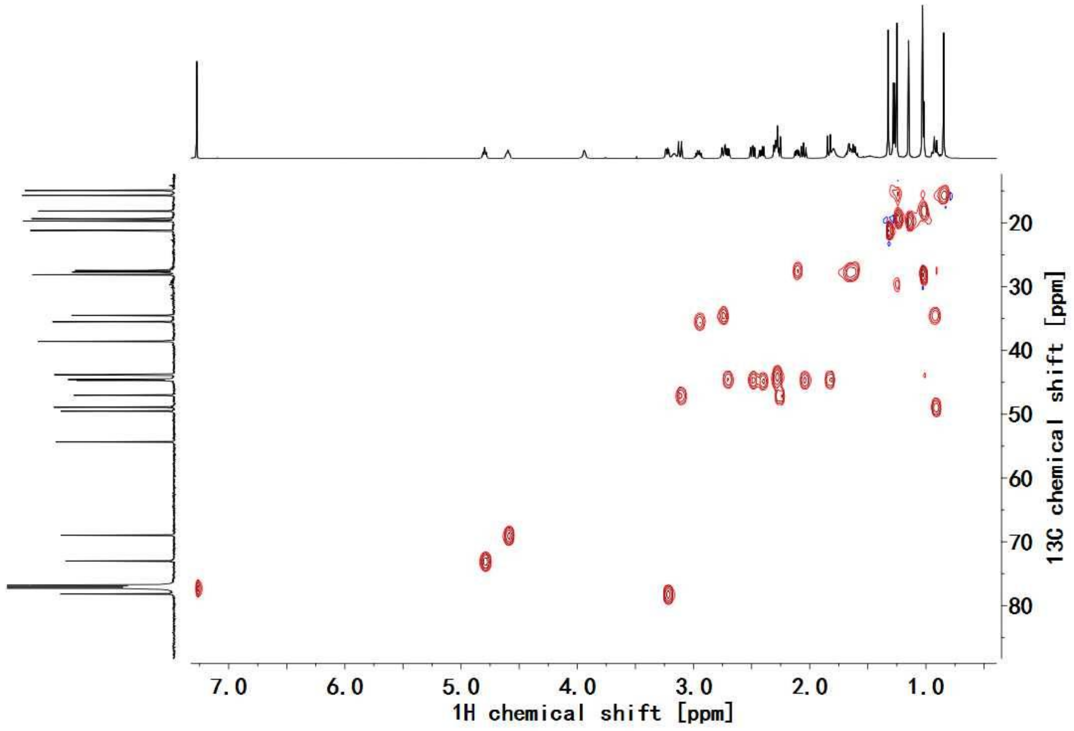

Figure S60. HSQC (600 MHz, $CDCl_3$) spectrum of **8**

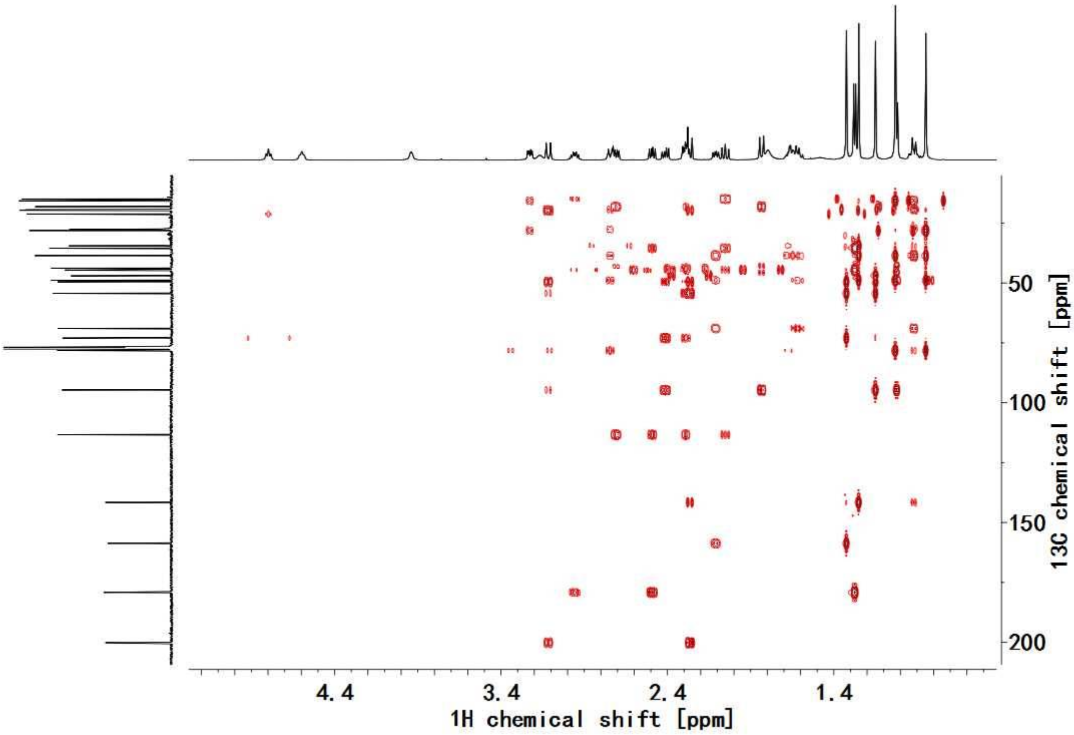

Figure S61. HMBC (600 MHz, $CDCl_3$) spectrum of **8**

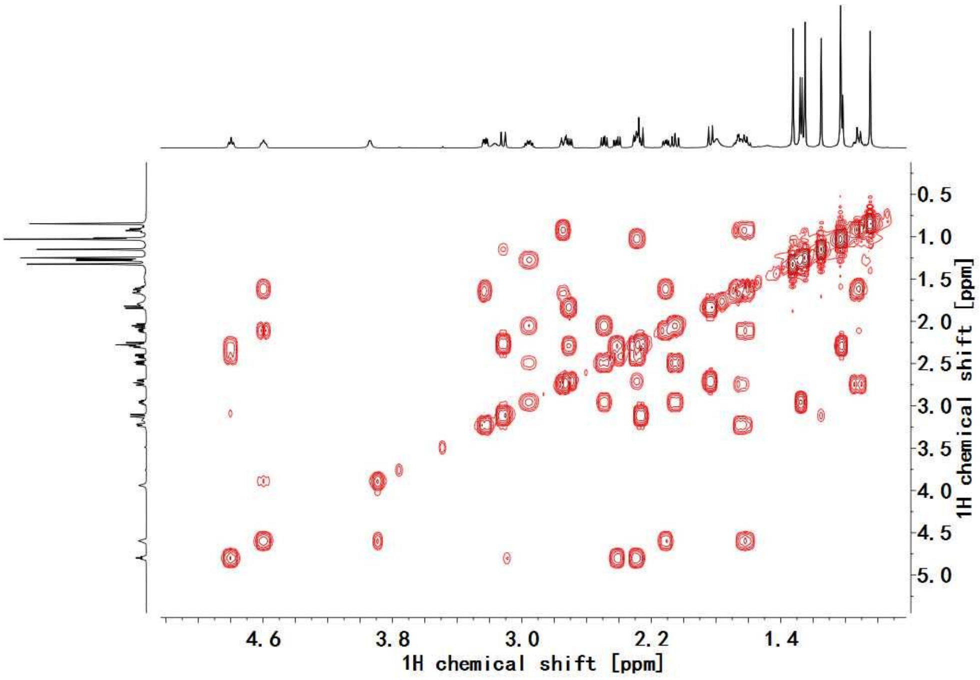

Figure S62. ^{1}H-^{1}H COSY (600 MHz, $CDCl_3$) spectrum of **8**

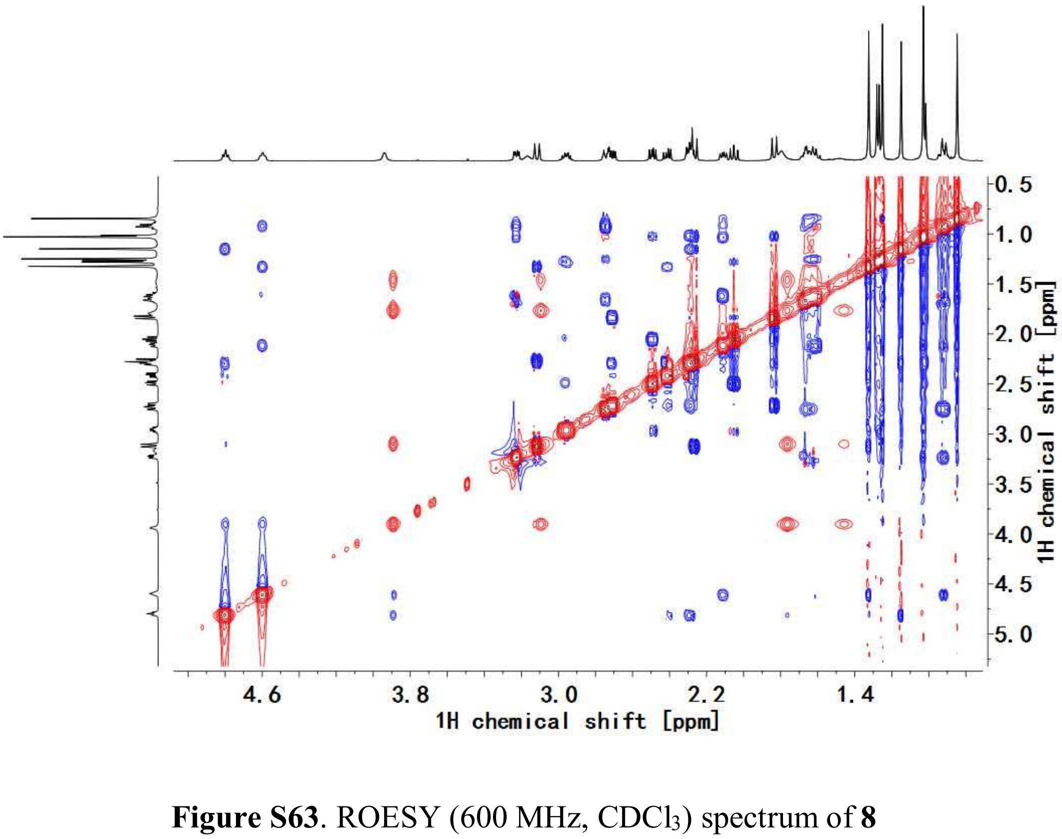

Figure S63. ROESY (600 MHz, $CDCl_3$) spectrum of **8**

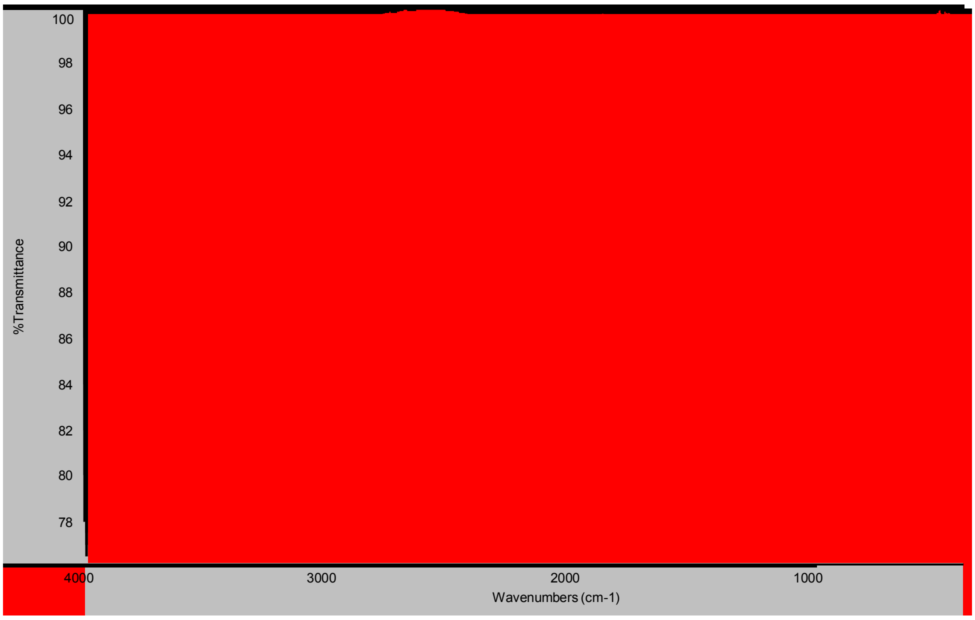

Figure S64. IR spectrum of **8**
